# Potential effects on the signaling network mediated by overexpression of the vitronectin gene in Hu sheep ruminal epithelial cells using multi-omics analysis

**DOI:** 10.3389/fgene.2026.1719513

**Published:** 2026-03-12

**Authors:** Bingqian Zhong, Luyu Ma, Hua Ni, Eli Subinur, Aiwen Zhu, Wei Yan, Yutao Wang

**Affiliations:** 1 Key Laboratory of Biological Resources and Ecology of Pamirs Plateau in Xinjiang Uygur Autonomous Region, College of Life and Geographic Sciences, Kashi University, Kashi, China; 2 School of Animal Science and Technology, Jiangsu Agri-animal Husbandry Vocational College, Taizhou, China

**Keywords:** ADM, Hu sheep, metabolome, rumen epithelial cells, transcriptome, VTN

## Abstract

Vitronectin (VTN) is a multifunctional extracellular matrix protein involved in cell adhesion, migration, and signal transduction. In this study, we constructed and transfected a *VTN* overexpression vector in Hu sheep ruminal epithelial cells (RECs) and performed a multi-omics analysis integrating transcriptomics and metabolomics. Compared with controls, 495 differentially expressed genes (DEGs) were identified (241 upregulated and 254 downregulated), primarily enriched in adhesion/mechanotransduction pathways such as ECM–receptor interaction and focal adhesion, as well as amino acid transport and membrane-related complexes; in contrast, translational preparatory processes including the spliceosome and aminoacyl-tRNA biosynthesis were suppressed. Metabolomics identified 103 differential metabolites (53 upregulated and 50 downregulated), prominently involving glycerophospholipid metabolism, nucleotide sugar biosynthesis, GPI-anchor biosynthesis, autophagy, and retrograde endocannabinoid signaling, indicating reinforced membrane lipid remodeling and membrane protein targeting. Multi-omics integration indicates that *VTN*, by remodeling ECM and membrane lipids, is associated with enhanced integrin–focal adhesion signaling and mechanotransduction, optimizes mitochondrial ATP production and energy utilization, and directs a programmed reconfiguration of lipid metabolism; concurrently, endocannabinoid-related pathways and “neurotransmission-like” signals such as NA–GABA were upregulated, providing an inhibitory/buffering tone against inflammation and environmental stress. Overall, *VTN* establishes a multilayered “adhesion–metabolism–repair” regulatory network that promotes rapid renewal and injury repair of RECs, offering a mechanistic basis and potential molecular targets for enhancing rumen function and production performance in ruminants.

## Background

The rumen epithelium is a critical site for nutrient absorption and metabolism in ruminants, directly influencing animal performance. As the interface between the ruminal environment and the organism, the tissue is not only responsible for absorption but also plays crucial metabolic and immunoregulatory roles (Pokhrel and Jiang, 2024).

Extracellular matrix proteins are fundamental in tissue development and functional maintenance. Among these, VTN is a multifunctional glycoprotein, mainly synthesized by hepatocytes and endothelial cells, and occurs in high concentrations in the blood (Seiffert et al., 1991).

Interactions of VTN with integrin receptors drive key processes such as cell adhesion, migration, and signaling (Preissner, 2011), including vascular barrier maintenance (Wu et al., 2001; Ayloo et al., 2022). The tissue-specific expression of *VTN* imparts unique impacts on immune and metabolic functions of RECs. Current research indicates that VTN interacts with integrin αvβ3 and αvβ5 to activate downstream FAK pathways, modulating inflammatory responses and metabolism (Feldinghabermann and Cheresh, 1993; Upton et al., 2008; Keasey et al., 2018). In immune regulation, VTN binds β2 integrins on neutrophils, coordinating aggregation and adhesion (Zuchtriegel et al., 2020), and is highly expressed in M2 macrophages, influencing activation and polarization (Peng et al., 2023; Zhang et al., 2024),which may be an important mechanism for its immunomodulatory role in different tissues. Additionally, VTN can activate JNK signaling via integrin αvβ6, regulating autophagy and energy balance (Huang et al., 1998; Gao et al., 2024). In the rumen, differential VTN expression may modulate insulin signaling and glucose metabolism through the cAMP/PKA/CREB pathway, supporting metabolic homeostasis (Ju, 2024; Ju et al., 2025), *VTN* may also regulate material transport and mitochondrial function (Wu et al., 2001; Visavadiya et al., 2020; Kariya and Nishita, 2025), further expanding our understanding of tissue-specific metabolic regulation.

During tissue injury, VTN-rich blood supports epithelial repair by promoting cell migration, granulation tissue formation, and scar remodeling (Jang et al., 2000). It also supports stem cell proliferation and microenvironment maintenance (Upton et al., 2008). Although *VTN* knockout mice appear largely normal, they exhibit impaired wound healing and delayed coagulation (Zheng et al., 1995), underscoring *VTN*’s importance in early tissue repair. VTN forms multiprotein complexes with other bioactive molecules, mediating cell behavior and morphogenesis (Preissner and Seiffert, 1998). In livestock, notably pigs, *VTN* genetic variations correlate with feed efficiency, with high-efficiency animals exhibiting higher serum VTN (Grubbs et al., 2016; Yan et al., 2019). Therefore, this study aimed to explore *VTN* overexpression effects on Hu sheep REC transcriptome and metabolome, uncovering its regulatory mechanisms and offering targets for optimizing rumen function.

## Materials and methods

### Cell culture and experimental design

Sheep ruminal epithelial cells (iCell-0038a) were purchased from Shanghai Saibaikang Biotechnology Co., Ltd. (Shanghai, China). These cells are an established cell line derived from Hu sheep ruminal tissue and were used in this study according to the supplier’s protocols. Experiments were conducted using cells at passage 6 (P6) to ensure physiological stability, as early passages (P1–P3) may retain immature phenotypes while late passages (P8+) exhibit senescence. Cells were cultured in DMEM/F-12 (Gibco, CA, United States) supplemented with 10% fetal bovine serum (FBS; Beyotime, Shanghai, China) and 0.25% penicillin-streptomycin (Solarbio, Beijing, China) at 37 °C in a humidified atmosphere containing 5% CO_2_.

The study employed a *VTN* overexpression group and an untreated control group, with three biological replicates per group for both transcriptomic and metabolomic analyses. For experiments, cells were seeded into 24-well plates (400 µL/well) and cultured until reaching 70%–80% confluence.

### Construction of the *VTN* overexpression vector

Total RNA was extracted from RECs using an RNA extraction kit (Tiangen, Beijing, China) and reverse transcribed. The coding sequence (CDS) of the ovine *VTN* gene (GenBank: XM_027975239.2) was amplified via a two-step PCR protocol. Initial amplification was performed using primers F1 (5′-TCA​GGC​ATC​AAA​GCA​GAG​ACC-3′) and R1 (5′-TGA​GCT​GGA​AGG​AGG​ATG​C-3′) with the following cycling conditions: 95 °C for 1 min; 30 cycles of 95 °C for 10 s, 52 °C for 15 s, and 72 °C for 2 min; followed by a final extension at 72 °C for 2.5 min.

A second amplification introduced restriction sites using primers F2 (5′-agt​CTC​GAG​CAT​GAC​ATC​CCT​AAG​GCC​CCT​TC-3’; *Xho* I site underlined) and R2 (5′-cgt​AGA​TCT​CTA​TGC​ATG​GCC​AGG​GAC​TG-3′; *Bgl* II site underlined) with an annealing temperature of 57 °C. The PCR product was gel-purified, cloned into the pGME-T vector, and subsequently subcloned into the pCAGGS-MCS mammalian expression vector via *Xho* I and *Bgl* II double digestion and T4 DNA ligation. The recombinant plasmid (pCAGGS-VTN) was validated by restriction enzyme digestion and sequencing.

### Cell transfection and sample collection

Transfection was performed using EndoFectin™ Max (GeneCopoeia, Guangzhou, China) according to the manufacturer’s protocol. Briefly, 1 µg of plasmid DNA and the transfection reagent were diluted in Opti-MEM™ I Reduced Serum Medium (Invitrogen, NY, United States), mixed to form complexes, and incubated for 10 min at room temperature. The complexes were added dropwise to the cells and incubated for 32 h. Following incubation, samples were harvested for multi-omics analysis. For transcriptomics, cells were lysed directly with TRNzol reagent (TransGen, Beijing, China) for 1 h and stored in cryovials. For metabolomics, the culture medium was removed, and cells were washed twice with PBS. Cells were detached via trypsinization (5 min, 37 °C), neutralized with complete medium, and centrifuged at 500 rpm for 5 min. The supernatants were discarded, and cell pellets were flash-frozen in cryovials at −80 °C. All samples were transported on dry ice to Sangon Biotech (Shanghai, China) for sequencing and analysis.

### Transcriptome analysis and reverse transcription quantitative PCR validation

#### Transcriptome QC and differential enrichment analysis

Cell samples were placed in a dry ice tank and sent to Shanghai Bioengineering Company for transcriptome sequencing analysis. RNA sequencing was conducted on the Illumina HiSeq platform with 150 bp paired-end reads. Raw sequencing data were assessed for quality using FastQC (v0.11.2). Quality control was then performed using Trimmatic (v0.36) to remove adapter sequences, low-quality bases, and reads shorter than 35 nt. Clean reads were mapped to the reference genome Hu sheep (T2T-sheep1.0. Ovis_aries) using HISAT2 (v2.1.0) with default parameters. StringTie (v1.3.3b) was used to quantify gene expression levels, and transcript abundance was calculated as transcripts per million (TPM).

Differential expression analysis was conducted using DESeq2 (v1.12.4), and genes with |log2FC| >1 and *P* value <0.05 were considered significantly differentially expressed. Gene ontology (GO) enrichment analysis was performed using topGO, while pathway enrichment analysis was conducted using clusterProfiler based on the Kyoto Encyclopedia of Genes and Genomes (KEGG) database. The significance threshold for enrichment analysis was set at a *P* value <0.05.

#### GSEA enrichment

Gene set enrichment analysis (GSEA) was performed using KEGG pathways from the MSigDB C2 collection (CP:KEGG_LEGACY). Human-sheep ortholog mapping was established using Ensembl Compara v114, and KEGG pathway gene sets were converted to sheep Ensembl gene IDs using the *Ovis aries* Rambouillet genome annotation (Ensembl v114). The transcriptomic TPM matrix was normalized, deduplicated, and log2-transformed (log2 (TPM+1)). Samples were stratified into high and low *VTN* expression groups based on the median expression level. Gene-wise log2 fold-change (log2FC) values were calculated for the high versus low expression contrast. Preranked GSEA was performed using sheep KEGG gene sets as the reference, and enrichment scores (ES/NES) and *P* values were computed. Results were visualized and filtered for metabolism-related pathways.

#### RT-PCR validation

To verify the reproducibility of the gene expression data, total RNA was extracted from *VTN* gene overexpression-treated cells, and eight DEGs, including *VIM*, *COX3*, *PRDX1*, *DDX5*, *MMp2*, *WARS*, *PLOD2*, and *SDC1* gene expression, were selected for reverse transcription quantitative PCR (RT-qPCR) analysis. The primer information for these DEGs is provided in Supplementary Table S1.

### LC-MS/MS metabolic spectroscopy determination

#### Sample processing and analysis

Samples were removed from the −80 °C freezer and thawed on ice. 500 μL of internal standard extract (methanol:water = 4:1, *V*/*V*) was added to the cell samples and vortexed for 3 min. The samples were then placed in liquid nitrogen for 5 min and on dry ice for 5 min, after which they were thawed on ice and vortexed for 2 min. This freeze-thaw cycle was repeated three times. The samples were centrifuged at 12,000 rpm for 10 min at 4 °C. 300 μL of the supernatant was collected and placed at −20 °C for 30 min. Then, the samples were centrifuged again at 12,000 rpm for 3 min at 4 °C. 200 μL of the supernatant was taken for LC-MS analysis. All samples were analyzed using two LC/MS methods. One sample was eluted using the positive ion mode through a T3 column (Waters ACQUITY Premier HSS T3 Column 1.8 μm, 2.1 mm * 100 mm) with 0.1% formic acid in water as mobile phase A and 0.1% formic acid in acetonitrile as mobile phase B. Gradient elution was performed as follows: 0–2 min, 5%–20%; 2–5 min, to 60%; 5–6 min, to 99%; 6–7.5 min, maintained at 99%; 7.5–7.6 min, reduced to 5%; 7.6–10 min, maintained at 5%. The analytical conditions were: column temperature 40 °C, flow rate 0.4 mL/min, and injection volume 4 μL. The other sample was analyzed using the negative ion mode, with the same elution gradient as that used for the positive ion mode.

#### Data collection

Data were collected in Information Dependent Acquisition (IDA) mode using Analyst TF 1.7.1 software. Raw MS data were processed using ProteoWizard to convert to mzXML format. Peak detection, alignment, and retention time correction were conducted using XCMS software. Peaks missing in more than 50% of the samples in each group were excluded, and KNN interpolation was applied to the remaining missing values. Peak areas were normalized using the SVR method.

#### Differential metabolite screening

Metabolite identification involved searches through internal standard libraries, integrated public databases, prediction libraries, and the metDNA method. Quality control filtering retained metabolites in QC samples with identification scores above 0.5 and CV values below 0.3. Data from both positive and negative ion modes were combined, selecting metabolites with the highest qualification levels and lowest CV values for further analysis. Metabolomics data were analyzed using multivariate statistical methods. Data were first log-transformed and normalized, then analyzed using principal component analysis (PCA) and orthogonal partial least squares discriminant analysis (OPLS-DA) in R software. Significantly different metabolites were identified using a variable importance in projection (VIP) score greater than 1 in the OPLS-DA model and statistical significance (*P* value < 0.05) in the Student t-test. Significantly different metabolites were annotated using the KEGG database.

### Integrated cross-species and multi-omics analysis

#### Phylogenetic profiling and chromosomal localization

To elucidate the evolutionary history of *VTN* and *ADM*, amino acid sequences were retrieved from the Ensembl and NCBI databases across diverse species, including *Homo sapiens*, *Mus musculus*, *Rattus norvegicus*, *Sus scrofa*, *Bos taurus*, *Capra hircus*, *Gallus gallus*, *Danio rerio*, and multiple *O. aries* breeds (Hu, White Dorper, Polled Dorset, Kermani, East Friesian, Romanov, Texel, and Qiaoke). Sequence alignment was performed using Clustal Omega, and the JTT + G substitution model was selected as the best-fit model based on the corrected Akaike Information Criterion (AICc). Phylogenetic trees were constructed using the Maximum Likelihood (ML) method (refined from an initial Neighbor-Joining tree) with 500 bootstrap replicates to assess node stability. Trees were visualized using the ggtree (v3.14.0) in R. Additionally, a joint alignment was conducted to delineate lineage relationships between the two gene families.

For chromosomal mapping, genomic coordinates were extracted from the *O. aries* reference genome (Ensembl v114; Oar_v3.1/Rambouillet). Analysis was restricted to primary chromosomes (1–26, X, and MT), and coordinates were validated against Ensembl BioMart to ensure consistency. The genomic distribution of *VTN* and *ADM* loci, integrated with differential expression profiles (log_2_FC), was visualized using the circlize R package.

#### Cross-dataset transcriptomic integration

To investigate expression conservation and functional networks, publicly available transcriptomic datasets were retrieved from the NCBI Gene Expression Omnibus (GEO). These included developmental time-series data for sheep and cattle stomach compartments. Specifically, abomasal and ruminal samples exhibiting high *ADM* expression were selected from Texel sheep (Yang et al., 2024) (10 d and 15 d; GSE227043, GSE200295) and Yak (Liu et al., 2023) (20 d and 15 m; GSE222396). Comparative datasets also included mouse liver and intestinal profiles (Bloks et al., 2025) (GSE110710) and feed efficiency studies in Charolais and Holstein cattle under divergent dietary regimes (Higgins et al., 2019) (GSE111464). For the latter, samples stratified by high versus low *VTN*/*ADM* expression were analyzed (Charolais zero-grazing samples were excluded due to insufficient sample size).

Differential expression analysis was performed using *DESeq2*, with significance defined as |log2FC| >1. These external datasets were intersected with the differentially expressed genes (DEGs) identified in *VTN*-overexpressing RECs from this study. Intersections of unique DEGs were visualized using *UpSetR*, and functional enrichment of shared gene sets was conducted using *clusterProfiler* based on the KEGG database.

### Single-cell analysis of bovine RECs

#### Single-cell integration and preprocessing

ScRNA-seq data from newborn calf and adult cattle rumen epithelium were obtained from NCBI (GSE183285 and GSE175652). Gene sets were intersected, and unique barcode prefixes (“Adult_,” “Newborn_”) were assigned prior to merging. The integrated dataset underwent normalization (NormalizeData), identification of highly variable genes (VST method), scaling (ScaleData), and Principal Component Analysis (PCA). Informative PCs were determined using JackStraw resampling and elbow plots. Batch effects were corrected in PCA space using *Harmony*, followed by UMAP dimensionality reduction, Shared Nearest Neighbor (SNN) graph construction, and clustering (Louvain/Leiden algorithms) at a resolution of 1.0.

#### Cell-type annotation

Cluster markers were identified using FindAllMarkers. A specificity score was calculated as:

$$\text{specificity}=\text{avg}\_\log2\text{FC}\times \left(\text{pct}.1-\text{pct}.2\right)$$

Clusters were ordered based on hierarchical clustering of Spearman correlations derived from average expression profiles. Annotation was guided by canonical epithelial markers (Basal: *KRT5*, *KRT14*, *ITGA6*; Spinous: *KRT6A*, *KRT10*, *S100A8/A9*; Granular: *CLDN1*, *CLDN4*; Proliferating: *MKI67*, *TOP2A*, *UBE2C*) and non-epithelial markers (endothelial, immune, fibroblast, smooth muscle). Clusters were manually assigned biological labels (e.g., BC1–3, SC1–2, cg-like SC, GC1–3, MC) based on marker expression patterns and the correlation tree. Cellular composition was quantified and visualized alongside UMAP projections.

#### ADM-focused subpopulation analysis

Given the absence of detectable *VTN* expression in the scRNA-seq control dataset, analysis focused on *ADM*. A subset of metabolically active epithelial subpopulations (BC1–3, cg-like SC, SC1–2, GC1–3) was extracted. Differential expression analysis was performed using FindMarkers to compare High vs. Low *ADM* expression groups and Adult vs. Newborn groups. Results were converted to Entrez IDs via bitr and subjected to KEGG pathway enrichment using enrichKEGG. Visualization included circular plots for the top 10 significant pathways and bubble plots for shared metabolism-related pathways.

#### Trajectory and pseudotime analysis

Pseudotime trajectories were constructed using Monocle3 (v1.4.26). To visualize *ADM*-associated dynamics, a composite panel integrating density, expression trends, and cellular composition was generated. Genes correlated with *ADM* (Pearson correlation, detected in ≥5% of cells) were identified. Pseudotime was binned into equal-frequency intervals for adult and newborn groups. Mean log1p expression was calculated for the top 30 positively and negatively correlated genes per bin, z-score normalized, and hierarchically clustered. The final visualization assembled overlapping density plots, LOESS-smoothed *ADM* expression trends, and heatmaps of correlated gene modules, revealing temporal patterns relative to epithelial differentiation status.

#### Cross-species reference similarity mapping

Combining human–sheep–cattle ortholog mappings (Ensembl Compara) with GTF annotations, we normalized the *VTN* experiment TPM table to generate a expression matrix for reference similarity analysis with single-cell clusters. For the nine epithelial subpopulations, we computed AverageExpression and performed row-wise z-score normalization:

$$Z_{gene}=\frac{X_{gene}-\mu_{gene}}{\sigma_{gene}}$$

where *Xgene* represents the original expression value, *µgene* is the mean expression across all samples, and *σgene* is the standard deviation of expression values for that gene. For the reference matrix, we averaged by condition (CONTROL, VTN), computed Pearson correlations between each cluster and both reference means using shared genes (*ADM*-Focused Group Analysis’s Shared metabolism related pathways genes):

$$r=\frac{\sum_{i=1}^{n}\left(Z_{X_{i}}-\bar{Z_{X}}\right)\left(Z_{Y_{i}}-\bar{Z_{Y}}\right)}{\sqrt{\sum_{i=1}^{n}{\left(Z_{X_{i}}-\bar{Z_{X}}\right)}^{2}\sqrt{\sum_{i=1}^{n}{\left(Z_{Y_{i}}-\bar{Z_{Y}}\right)}^{2}}}}$$

For Z-score normalized data, this simplifies to:

$$r=\frac{1}{n-1}\sum_{i=1}^{n}Z_{X_{i}}\cdot Z_{Y_{i}}$$

We then calculated the difference between correlations with each reference group:

$$\Delta r=r_{\text{VTN}}-r_{\text{Control}}$$

where positive values indicate cluster expression patterns more similar to the VTN-treated profile. We exported the results table and visualized differential correlations using a heatmap.

#### Data analysis

Quantitative PCR (qPCR) was performed using the 2^^−ΔΔCt^ method to calculate relative gene expression. *β-actin* was used as the internal reference gene, and all reactions were performed in triplicate. The formula

$$\Delta \Delta C_{t}={\left(\Delta C_{t,\text{target}}-\Delta C_{t,\beta ‐\text{actin}}\right)}_{\text{experimental}}-{\left(\Delta C_{t,\text{target}}-\Delta C_{t,\beta ‐\text{actin}}\right)}_{\text{control}}$$

was used to normalize and quantify gene expression, and visualized using graphpad prism 10.4.0. Other analyses were performed and visualized using the Base Package of the R programming language.

## Results

### Transcriptomic remodeling of RECs upon *VTN* overexpression

#### Global transcriptional shifts and *VTN* overexpression efficiency

**FIGURE 1:**
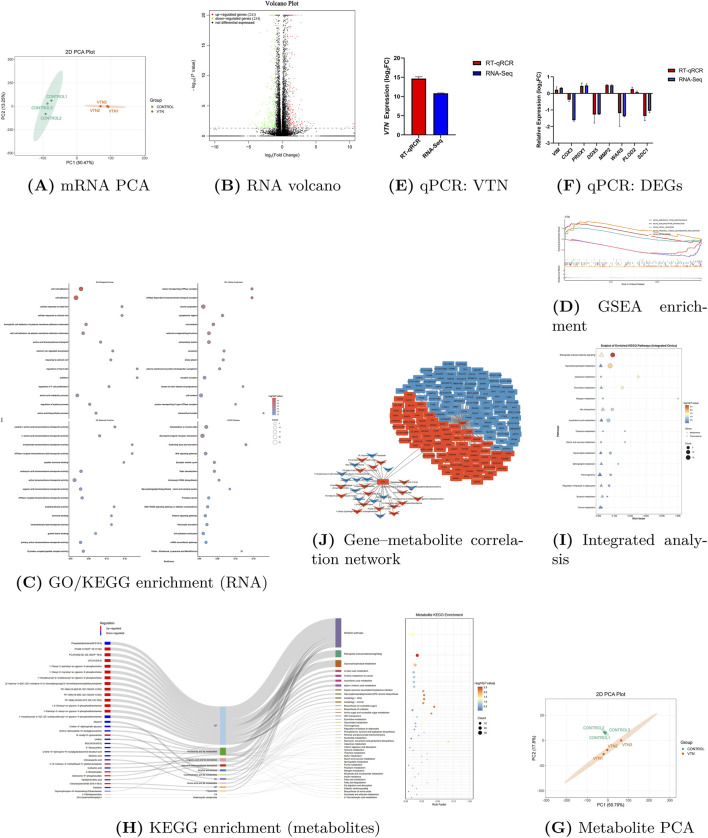
**(A–F)** Transcriptome: PCA, volcano, GO/KEGG enrichment, GSEA, and qPCR validations. **(G–J)** Metabolome and integrated analyses: metabolite PCA, KEGG enrichment, integrated Sankey/joint enrichment, and the gene–metabolite correlation network.

High-throughput RNA sequencing of *VTN*-overexpressing RECs generated 75.5 Gb of clean data (approx. 14.07 Gb per sample) with a Q30 base percentage exceeding 94.67%, ensuring high-quality coverage. Principal Coordinates Analysis (PCoA) revealed distinct separation between the *VTN* and control groups, confirming consistent transcriptional reprogramming (Figure 1A). We identified 495 differentially expressed genes (DEGs) (|log2FC| > 1, *P* value <0.05), comprising 241 upregulated and 254 downregulated genes (Figure 1B). As expected, *VTN* was the most significantly upregulated gene (log2FC > 24), validating the overexpression model (Supplementary Table S1). Other top upregulated genes included *ADM* (4.67-fold), *SPP1* (2.44-fold), *CCN1* (2.10-fold), and *ARL6IP1* (2.02-fold), whereas *COX3*, *PSAT1*, *DDX5*, *WARS1*, and *FUS* were significantly downregulated despite remaining highly expressed. Notably, *VTN* overexpression altered chromosomal read coverage (increasing in most, decreasing in CP162245.1 and CP162267.1) and influenced post-transcriptional processing; specifically, the significantly upregulated *ADM* gene exhibited a reduction in alternative splicing events compared to controls (Table 1).

**TABLE 1:** *ADM* gene alternative splicing.

Gene ID	Gene name	Group	Postion	Alternative 5′ or 3′end splicing	Alternative splicing sites
R6Z07_014347	*ADM*	VTN	CP162254.1 [-] 62926939-62927888	0	​
R6Z07_014347	*ADM*	CONTROL	CP162254.1 [-] 62926939-62927888	2	CP162254.1 [-] 62927791-62928256 CP162254.1 [-] 62927791-62927909

#### Functional enrichment: the adhesion–structure–transport nexus

Functional annotation of DEGs (397 GO, 158 KEGG) revealed that *VTN* overexpression drives a systemic alteration of epithelial architecture. Gene Ontology (GO) analysis (Figure 1C) highlighted a primary impact on Biological Processes (BP) related to cell–cell adhesion (GO:0098609), cellular response to calcium ions (GO:0071277), and amino acid transmembrane transport (GO:0003333), suggesting a role in reinforcing epithelial integrity and communication. Cellular Component (CC) enrichment confirmed *VTN*’s localization and function, involving extracellular matrix (ECM) organization, plasma membrane complexes, and vesicular transport systems. Molecular Function (MF) analysis pointed to modulation of transmembrane transporter activity, peptide hormone/growth factor binding, and cell adhesion molecule binding.

KEGG pathway analysis further corroborated these structural shifts, identifying enrichment in Neuroactive ligand–receptor interactions, ECM–receptor interaction, Cell adhesion molecules, and the Wnt signaling pathway. Collectively, these results suggest that *VTN* overexpression remodels the RECs architecture and cell–cell interactions, potentially influencing barrier function and nutrient absorption capacity.

#### GSEA and validation

Gene Set Enrichment Analysis (GSEA) provided directional insight (Figure 1D): the Spliceosome and Aminoacyl-tRNA biosynthesis pathways were significantly negatively enriched, indicating a broad downregulation of post-transcriptional processing and translational machinery. In contrast, ECM–receptor interaction and Focal adhesion pathways were significantly positively enriched, consistent with *VTN*’s role in enhancing integrin-mediated adhesion signaling. RT-qPCR validation of *VTN* and eight randomly selected DEGs showed expression patterns consistent with RNA-seq data (Figures 1E,F), confirming the reliability of the transcriptomic profiling.

### Metabolomic reprogramming: lipid-centric membrane remodeling

#### Differential metabolite profiling

Untargeted metabolomics identified 1733 metabolites. Multivariate analysis (PCA and OPLS-DA) demonstrated robust separation between groups (R2Y > 0.99, Q2 > 0.9; Figure 1G). We identified 103 differential metabolites (VIP >1, *P* value < 0.05), with 53 upregulated and 50 downregulated. Classification via HMDB revealed a predominance of lipids and nucleotide derivatives (Supplementary Table S1).

Top upregulated metabolites included membrane lipids such as Phosphatidylethanolamine (PE), Phosphatidylcholine (PC), ether lipids, and Galactosylceramide (GalCer). Conversely, downregulated metabolites included Glycerophospho-N-Arachidonoyl Ethanolamine, Threitol, and reductive/detoxification markers. This signature—marked by the elevation of structural phospholipids (PC, PE) and reduction of arachidonic acid-related species—suggests a *VTN*–integrin axis-driven enhancement of membrane construction and adhesion signaling, coupled with a decrease in the pro-inflammatory lipid pool. (Note: Early-phase lipid upregulation may be partially influenced by the liposome transfection reagent, though the specific remodeling of ether lipids and GalCer points to biological regulation).

#### Metabolic pathway enrichment

Differential metabolites were enriched in Retrograde endocannabinoid signaling, Biosynthesis of nucleotide sugars, Autophagy, GPI-anchor biosynthesis, and Amino sugar/nucleotide sugar metabolism (Figure 1H). These pathways collectively indicate a lipid-derived signaling shift supporting membrane turnover (autophagy), improved protein targeting (GPI anchoring), and enhanced glycosylation capacity (nucleotide sugars) necessary for ECM and cell-surface protein maturation.

### Integrated transcriptome–metabolome analysis

#### Convergent pathways and the NA-GABA hypothesis

Integrated analysis identified 15 commonly enriched pathways (Figure 1I), including Glycerophospholipid metabolism, ABC transporters, and Retrograde endocannabinoid signaling. Notably, lipid-related pathways showed stronger enrichment in the metabolome than the transcriptome, indicating that lipid remodeling is the primary functional readout of *VTN* overexpression. Within the endocannabinoid pathway, transcriptomic changes (*COX3*, *GNG7*, *CNR1*) aligned with metabolomic shifts to suggest that the downstream metabolite NA-GABA may serve as a key signaling mediator.

#### Gene–metabolite correlation network

A Spearman correlation network (|ρ| > 0.8, *P* value < 0.05) visualizing *VTN* and NA-GABA interactions revealed a distinct topology (Figure 1J). *VTN* acted as a central hub positively correlated with numerous phosphatidylcholines and glycerol intermediates. Simultaneously, a specific gene module showed coordinated correlations with NA-GABA. Additionally, components of ABC transporters (e.g., *ABCG1*) co-occurred with metabolites like N-acetyl-D-glucosamine and uridine, while sphingolipid metabolism showed increased ceramide concurrent with reduced galactosyltransferase expression. This network underscores a “VTN–Lipid–Neuroactive” axis, where *VTN* drives lipid-based membrane remodeling while NA-GABA potentially buffers signaling via neuro-lipid coupling.

### Integrated cross-species and evolutionary analysis

#### Phylogenetic and chromosomal conservation of *VTN* and *ADM*

**FIGURE 2:**
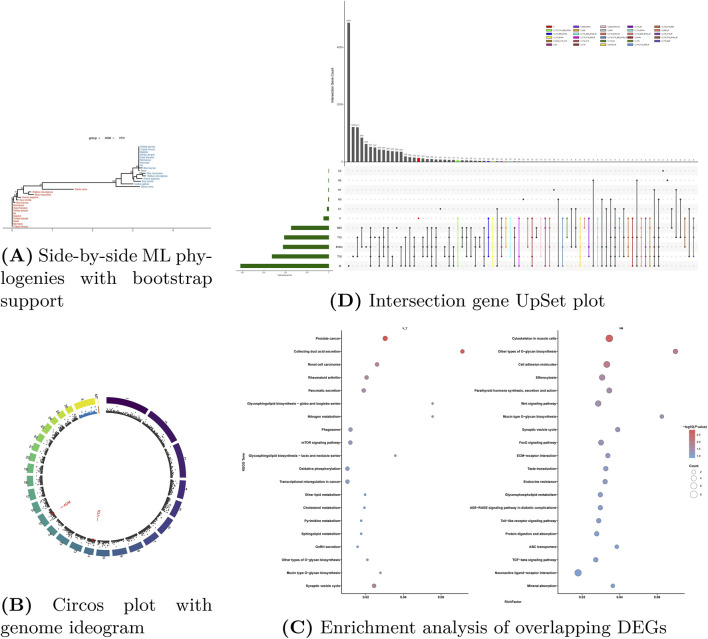
Multi-dimensional analysis of *VTN* and *ADM* genes. **(A)** ML phylogenies with bootstrap support values. **(B)** Circos plot showing genomic locations **(C)**. Enrichment analysis of DEGs comparing V vs. M (left) and V vs. T10/T15/B20/B15m (right). **(D)** Intersection gene UpSet plot across groups C2, H2, H1, H0, C1, V, B20, T15, B15m, T10, and M.

Phylogenetic analysis confirmed that *VTN* and *ADM* belong to distinct paralogous families (Figure 2A). Both genes displayed a “species-first” clustering pattern: ruminants (cattle, sheep, goats) formed a coherent, high-support clade (bootstrap 98–100), distinct from rodents and other mammals. This strong conservation within ruminants supports the translational relevance of the ovine model. Chromosomal mapping localized *VTN* to the mid-lower region of ovine chromosome 11–12 and *ADM* to the mid-anterior of chromosome 15–16 (Figure 2B), with no structural anomalies.

### Intersection enrichment of *VTN*/*ADM*-linked gene sets across species and tissues

#### Cross-tissue and cross-species gene intersection

Intersection of our DEG data with public datasets (sheep/cattle stomach development, mouse gut/liver, cattle feed efficiency) identified conserved expression modules (Figures 2C,D; Table 2).

**TABLE 2:** Combined analysis of *VTN*/*ADM* gene expression patterns across different tissue groups and species.

Group	Gene set	Gene expression pattern
V_H0_H1_H2_C1	*VTN*/ADM feed efficiency-related genes in Liver: *DLL1, CFD, CDKN1C, CAPS, ARHGEF16, SAP25, TNNT1*	Upregulated: *CFD, CDKN1C, CAPS, ARHGEF16, SAP25, DLL1;* Downregulated: *TNNT1*
V_M_T10_T15_ B20_B15m	Common intersection genes in liver and gastrointestinal Tract: *ARHGEF16, VWA5B2, UPP1, TSPAN13, TSPAN1, TMPRSS2,* *SULT2B1, SLC22A15, SH3RF2, RHOV, RAP1GAP2, PLCH2,* *PHYHD1, MCF2L, LIPG,* *KCNN3, INSRR, GRAMD1B, GCA, GALNT5, FRZB, ERN2* *CPVL, CLCA2, CELSR2, CAPN6, ATP9A, ARNT2, ADAMTS15*	Differential gene expression patterns in sheep/cattle rumen compared with abomasum Upregulated: *UPP1, SULT2B1, RHOV, PLCH2, KCNN3, CLCA2, CELSR2, LIPG;* Downregulated: *SLC22A15, PHYHD1, FRZB, CAPN6, ARHGEF16, VWA5B2, TSPAN1, TSPAN13, TMPRSSS2, MCF2L, INSRR,* *RAP1GAP2, GCA, GRAMD1B, GALNT5, ATP9A, ARNT2, ADMTS15, SH3RF, ERN2, CPVL* Downregulated: *UPP1, SULT2B1, RHOV, PLCH2, KCNN3, CLCA2, CELSR2, INSRR, GRAMD1B, ARHGEF16, VWA5B2, TSPAN13, TSPAN1, TMPRSSS2, RAP1GAP2,* *MCF2L, GCA, GALNT5, ERN2, CPVL,* *ATP9A, ARNT2, ADMTS15*

Feed Efficiency-Related Genes: A common set (e.g., *CFD, CDKN1C, CAPS, DLL1*) was upregulated in high-feed-efficiency liver samples, linking *VTN* networks to metabolic performance.

Tissue-Specific Signatures: Comparison across liver and gastrointestinal tracts identified a shared core of genes involved in cholesterol metabolism and O-glycosylation. However, expression patterns diverged between tissues (e.g., *VTN* higher in liver, *ADM* higher in rumen), suggesting a division of labor where *VTN* orchestrates tissue-specific structural and secretory functions essential for nutrient absorption.

### Single-cell resolution of the ADM axis

#### Cellular landscape of the bovine rumen epithelium

**FIGURE 3:**
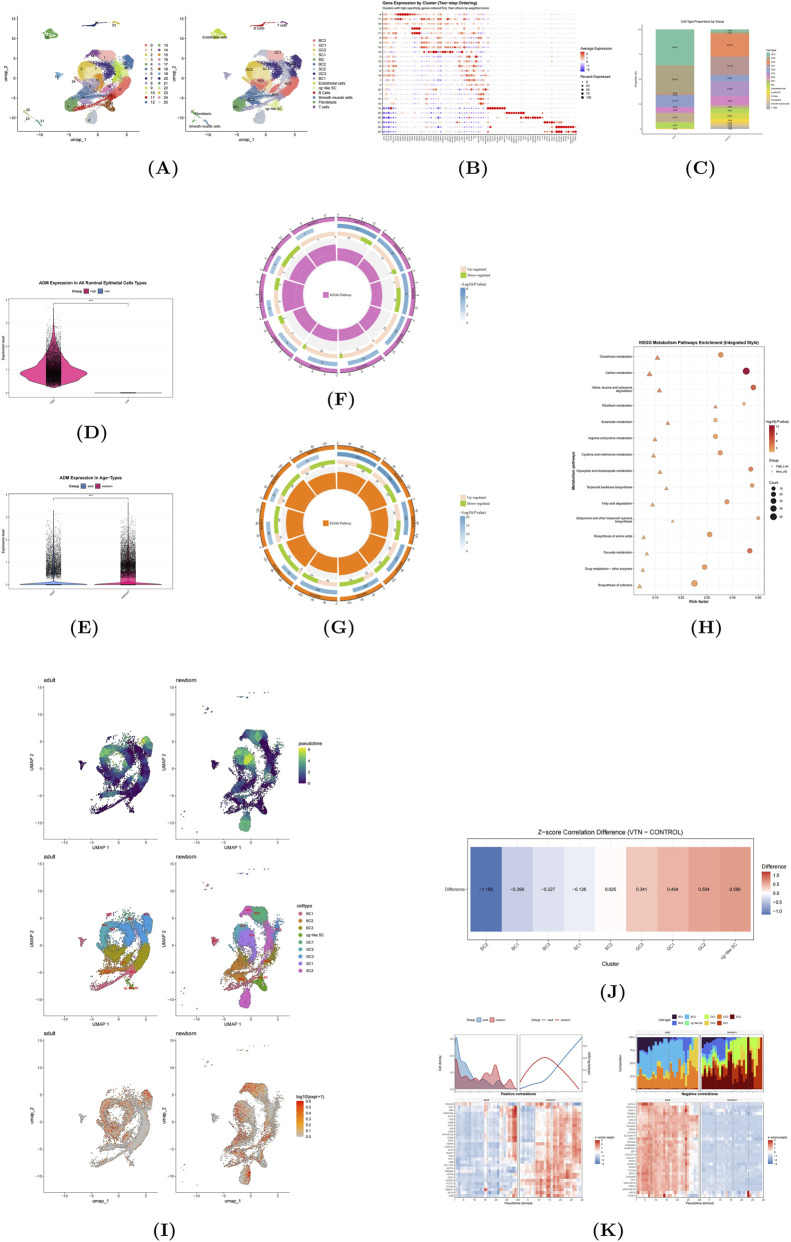
Comprehensive single-cell RNA-seq analysis. **(A)** UMAP projection of cell clusters. **(B)** Dot-plot heatmap of marker gene expression. **(C)** Cell-type composition by group. **(D)** ADM expression (High vs. Low). **(E)** ADM expression (Adult vs. Newborn). **(F,G)** KEGG enrichment patterns in two contrasts. **(H)** Shared KEGG metabolism. pathway enrichment. **(I)** Pseudotime comparative analysis. **(J)** Directional convergence to VTN overexpression. **(K)** Pseudotime-resolved density, ADM trends, cell-type composition, and gene correlations.

Integration of newborn and adult bovine rumen scRNA-seq data produced a high-resolution atlas (Figure 3A). We annotated distinct epithelial strata: Basal Cells (BC1-3) (*KRT5/14*), Spinous Cells (SC1-2, cg-like SC) (*KRT10/S100A8*), Granular Cells (GC1-3) (*CLDN1/4*), and Mitotic Cells (MC) (*MKI67*), alongside non-epithelial populations (Figure 3B).

Age-Dependent Composition: Adult tissue was dominated by homeostatic basal cells (BC3), whereas newborn tissue exhibited a higher proportion of proliferative (MC) and differentiating layers (SC/GC), reflecting active growth and remodeling (Figure 3C).

#### ADM as a developmental and metabolic tuner

To dissect the functional relevance of *ADM*, we stratified cells based on expression intensity and donor age. This revealed a robust separation between “High” and “Low” *ADM* groups (Figure 3D). Furthermore, we identified a pronounced age bias, where *ADM* was significantly upregulated in newborns relative to adults (Figure 3E), a pattern consistent with the distinct proliferative and metabolic requirements of neonatal epithelium.

#### Functional enrichment of ADM-high populations

KEGG analysis of *ADM*-associated signatures revealed distinct functional axes (Figures 3F,G):

Differentiation and Stress Axis: Top pathways included Cornified envelope formation, Apoptosis, and Foxo signaling, linking *ADM* to epithelial maturation and stress responses.

Metabolic Axis: A robust “mitochondrial–energy” signature (Oxidative phosphorylation, Carbon metabolism) was enriched in *ADM*-high and newborn cells. Bubble plots (Figure 3H) confirmed a shared “baseline metabolic network” (Carbon flux, BCAA degradation, Glutathione) driven primarily by age (Newborn > Adult) but fine-tuned by *ADM* levels. This places *ADM* as a modulator that amplifies energy and antioxidant capacity during rapid growth.

#### Convergence of *VTN* overexpression with single-cell states

To map *in vitro* findings to *in vivo* states, we performed a similarity analysis (Figure 3J). *VTN*-overexpressing transcriptomes showed the strongest positive correlation with Granular (GC) and cg-like Spinous subpopulations (cg-like SC). Conversely, they diverged from Basal (BC) profiles. This suggests that *VTN* drives epithelial cells toward a more differentiated, metabolically active state resembling the granular layer, rather than maintaining a basal progenitor phenotype.

#### Pseudotime trajectory and the “ADM–differentiation” switch

Monocle3 pseudotime analysis reconstructed the differentiation trajectory (Figure 3I). Newborn cells occupied a compact, smoother trajectory enriched in early/mid-stage precursors, while adult cells were dispersed in late-stage branches.

A composite temporal analysis (Figure 3K) revealed a critical switch:

Early Pseudotime (Newborn-dominant): *ADM* peaks alongside proliferative and stress-response genes (*TAGLN2, GADD45A*), supporting progenitor maintenance and metabolic activation.

Late Pseudotime (Adult-dominant): As differentiation proceeds, *ADM* declines, inversely correlating with keratinization and lipid-maturation markers (*GSTA1, PSAT1*).

This spatiotemporal pattern indicates that *ADM* functions as a “tuner,” supporting early-stage metabolic activation and progenitor maintenance, before receding to allow terminal differentiation and barrier maturation.

## Discussion

The rumen acts as a dynamic fermentation chamber, continuously receiving substrate input and expelling fermentation products (Palmonari et al., 2024). This environment sustains a complex symbiosis: rumen microbes provide volatile fatty acids (VFA) and microbial proteins to the host, while the host delivers a stable microenvironment and nutrients to the microbes (Newbold and Ramos-Morales, 2020; Liu et al., 2021). This reciprocal relationship optimizes fiber utilization, which underpins ruminant nutrient metabolism (Mccann et al., 2014; Gruninger et al., 2019). RECs provide a critical structural and functional barrier between the digestive tract and systemic circulation. Through coordinated networks of tight, adherens, and gap junctions, RECs ensure the integrity of the rumen lining, supporting nutrient absorption and metabolism while preventing harmful intrusion (Steele et al., 2011; Ma et al., 2021). As REC function hinges on adhesion-centric ECM signaling, VTN emerges as a pertinent target. Although absent from single-cell and tissue transcriptomes, RT-qPCR detection and successful cloning confirm its expression, supporting a focused evaluation of its role in REC adhesion and signaling.

*VTN* overexpression reprograms RECs within the dynamic, microbe–host symbiosis of the rumen by prioritizing adhesion/ECM signaling and de-emphasizing splicing/translation: transcriptomics shows positive enrichment of ECM–receptor and focal adhesion with negative enrichment of spliceosome and aminoacyl-tRNA biosynthesis, while metabolomics supplies material corroboration—PC/PE/ether lipids and GalCer rise to furnish a lipid foundation for integrin clustering and membrane microdomain stability, arachidonic-acid–related LPE and reduction/detox markers fall, and metabolome-specific enrichments in autophagy and GPI-anchor biosynthesis (plus boosted nucleotide-sugar supply) reveal post-transcriptional/substrate-level control that complements RNA-level programs; temporal and technical factors (e.g., transient liposomal lipid loading) may inflate early PC/PE signals, whereas sustained ECM/FA upregulation likely reflects a VTN–integrin–driven steady-state shift; these layers indicate a move toward homeostatic construction—membrane and matrix assembly, lower inflammatory tone, and reduced bulk protein-biosynthesis load—achieved through parallel, partially separable nucleic-acid regulation and lipid remodeling, with *ADM* emerging as a lipid-responsive modulator: evidence across datasets indicates that isolated *VTN* upregulation does not consistently elevate *ADM* mRNA, whereas lipid-rich conditions may associate with increased *ADM* expression, supporting a metabolically tuned role whereby *ADM* links metabolic status to gene-regulatory outcomes and may fine-tune epithelial differentiation; although the precise lipid influence on *ADM* transcription/splicing remains unresolved—yet plausible given the down-shift of spliceosome/translation—this warrants targeted validation and illustrates nucleic-acid–lipid functional complementarity that helps RECs balance structure, metabolism, and barrier homeostasis in a fluctuating rumen environment.

### Matrix Remodeling

**FIGURE 4:**
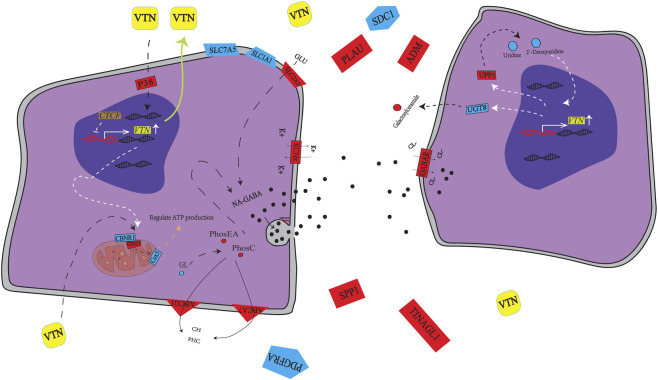
*VTN* gene overexpression affects some molecular changes in the pathway. Red and yellow indicate upregulation of genes or metabolites; blue indicatesdownregulation of genes and metabolites. Abbreviations: *SDC1*, Syndecan 1; *PLAU*, Plasminogen activator, urokinase; *ADM*, Adrenomedullin; *SLC7A5*, Solute carrier family 7 member 5; NA-GABA, N-arachidonoyl-gamma-aminobutyric acid; *CNR1*, Cannabinoid receptor 1; *UPP1*, Uridine Phosphorylase 1; GLU, glucose; *SPP1*, Secreted Phosphoprotein 1; *CTCF*, CCCTC-binding factor; *GABAR*, GABA receptor; *UGT8*, UDP glycosyltransferase 8; *TINAGL1*, Tubulointerstitial nephritis antigen like 1; *KCN*x, Potassium voltage-gated channel subfamily;*PGDFRA* = Platelet Derived Growth Factor Receptor Alpha, etc.

Changes were observed in cell-matrix interaction genes (Figure 4). *SDC1* (a key cell adhesion receptor) and *PDGFRA* (a receptor for platelet-derived growth factors) were downregulated, while *PLAU* (Urokinase-type plasminogen activator) was upregulated, suggesting reduced cell adhesion and altered growth factor signaling dynamics. *SDC1* is known for mediating cell–cell communication, exosome formation, and cytoprotection under stress, often regulated through VTN interaction and signaling (Morgan et al., 2007; Kumar et al., 2015; Lee et al., 2023). *VTN* and *SDC1*, along with others such as *TINAGL1* and *SPP1* (Osteopontin), are collectively involved in ECM structuring, tissue remodeling, and the regulation of both integrin and growth factor pathways. The upregulation of *SPP1* and *TINAGL1* supports enhanced tissue remodeling and migration processes, while their interplay with *VTN* points to functional synergy in maintaining and renewing the epithelial barrier (Sato et al., 2022; Lee et al., 2024). Overexpression of the *VTN* gene led to a reduction in the selective shear site of the *ADM* gene’s mRNA, potentially stimulating *ADM* gene expression directly. Adrenomedullin (*ADM*), a multifunctional peptide hormone from the Calcitonin Gene-Related Peptide (CGRP) family, is widely expressed throughout the gastrointestinal tract and acts as a gastrointestinal hormone regulating various physiological processes such as gastric emptying, gastric acid release, insulin secretion, defecation, and intestinal barrier function. It also enhances vascular and lymphatic regeneration and function, mucosal epithelial repair, and modulates the microbiome composition by reducing harmful flora (e.g., Enterobacteriaceae) and increasing beneficial flora (e.g., *Lactobacillus* and Bifidobacterium) (Fischer et al., 2020; Martínez-Herrero and Martínez, 2022). The upregulation of *ADM* suggests that *VTN* overexpression may directly or indirectly promote tissue repair, barrier fortification, and local immune regulation.

### Cellular metabolism and adaptive reprogramming

At the level of cellular metabolism and adaptive reprogramming, *VTN* overexpression induces a coordinated shift in epithelial cells centered on adhesion and microenvironmental adaptation: bulk transcriptomics show upregulation of substrate uptake and anabolic entry points (*SLC2A3* promoting glucose uptake; *UPP1* promoting uridine salvage into nucleotide synthesis), accompanied by differential expression of genes involved in mitochondrial respiration and nutrient metabolism (*COX3* indicating ETC-IV; PSAT1 involved in amino acid metabolism; *INSIG1/ANGPTL8* regulating lipid metabolism). Together, these changes support a bias toward glycolysis and anabolic metabolism, consistent with adaptation to a potentially hypoxic, shear- and acid-stressed epithelial surface. Physiologically, this suggests enhanced glycolytic flux, a relative decrease in OXPHOS, and retuning of lipid metabolism, indicating a rapid renewal/proliferation program under continuous environmental challenge and repair demands. GSEA further shows significant activation of ECM–receptor interaction and focal adhesion, implying strengthened integrin–FAK/Src–RhoA signaling, increased adhesion/spreading/migration, cytoskeletal remodeling, and augmented mechanotransduction; meanwhile, the spliceosome and aminoacyl-tRNA biosynthesis are significantly suppressed, with a downtrend in the proteasome, reflecting a systemic “downshift” in post-transcriptional processing and translational priming. This matches a “resource reallocation” model: reducing the energy and substrate burden of protein production/turnover and redirecting capacity toward ECM-mediated mechanical adaptation. At the metabolic pathway level, shifts are moderate—glycolysis/gluconeogenesis shows nominal upregulation, whereas TCA/OXPHOS/lipid metabolism tend toward downregulation.

Single-cell integration with an *ADM* focus shows: *ADM* is significantly higher in newborn than in adult and peaks in the early pseudotime of newborn cells, co-regulating with proliferation and metabolic activation modules before receding; in the adult mid-to-late pseudotime, *ADM* rises only modestly and is negatively coupled with differentiation/keratinization and lipid-metabolic maturation, revealing an inverse relationship between *ADM* and terminal differentiation. Cross-dataset evidence also suggests *ADM* expression more consistently tracks lipid-rich/metabolically active contexts rather than a uniform, direct transcriptional response to *VTN*. In terms of cell-type sensitivity, GC and cg-like SC move closest to the “*VTN* signature” after *VTN* treatment, consistent with their metabolically/antioxidant-program-active phenotypes and dynamic *ADM* behavior; by contrast, BC2 remains closer to the control state, suggesting potential suppression or state diversion under *VTN*.

This yields a concise working model: (1) enhanced ECM engagement via integrin–FAK/Src–RhoA drives increased adhesion and mechanosignaling; (2) a “downshift” of splicing/translation/protein turnover reallocates resources; (3) a mild glycolytic bias with tuned lipid metabolism enables rapid renewal at a stress-exposed surface; (4) *ADM* is upregulated in early/immature states to promote glycolysis and antioxidation and declines as the keratinized barrier matures; (5) at the cell-type level, *VTN* preferentially reprograms GC and cg-like SC toward metabolically active, ECM-engaged states while shifting BC2 away from its control baseline, thereby coupling “adhesion mechanics—metabolic state—lineage progression.”

### Inflammation, stress response, and neuroendocrine integration

*GNG7* and *MAPK13* genes are significantly upregulated. GNG7 is a γ subunit of heterotrimeric G proteins involved in G protein-coupled receptor (GPCR) signal transduction (Kankanamge et al., 2022). When GPCRs are activated, G protein subunits can trigger multiple downstream signaling pathways, including MAPK cascades. The concurrent upregulation of *GNG7* and *MAPK13* may represent mutually enhancing pathway activation, where G protein signaling ultimately leads to activation of the p38 MAPK family (including *MAPK13*) through the RAS-RAF-MEK pathway or alternative routes (Lu et al., 2019). *MAPK13* plays a crucial role in inflammatory responses by regulating the production of pro-inflammatory cytokines and chemokines (Yang et al., 2014; Wang et al., 2024). When enhanced together with GNG7 signaling, this may result in a stronger inflammatory response. The both genes may jointly participate in NF-κB pathway and inflammasome activation, which are core mechanisms in various inflammatory diseases (Liu D. et al., 2022). This co-upregulation may influence the recruitment and function of macrophages, neutrophils, and T cells (Prame Kumar et al., 2018), enhancing both innate and adaptive immune responses. In breast epithelial cancer cells, VTN binding to integrins regulates downstream gene expression through the NF-κB pathway (Reuning, 2011).Similarly, *VTN* gene overexpression in RECs also enhances the regulation of genes related to the immune microenvironment, such as *CFD*, *TNFSF9*, *TREM1*, and *GCA*, which is Similar with *VTN*’s role in regulating ECM changes and IL6 inflammatory factor function (Keasey et al., 2018).

Multi-omics integration and Figure 1J network define an upstream, lipid-centric state that calibrates inflammation under *VTN* overexpression. Strengthening of the VTN–ECM–integrin axis elevates adhesion/mechanosensing and cytoskeletal remodeling (ECM–receptor interaction, focal adhesion), which cross-talks with GPCR/p38 signaling to prime NF-κB/inflammasome activity. The network positions *VTN* at a lipid-metabolite hub tightly linked to phosphatidylcholines, lysophosphatidylcholines, and glycerol intermediates, matching metabolomic increases in PC/PE/ether lipids and GalCer and decreases in arachidonate-linked LPE. An opposing NA-GABA–centered gene cluster (with CNR1 and GABA receptors) indicates reinforced coupling between metabolic state and inhibitory neuro-lipid signaling. ABC transporters (e.g., *ABCG1*) co-occur with glycerol and nucleotide-sugar metabolites, supporting coordinated lipid handling and glycosylation capacity, while trends in sphingolipid and pyrimidine metabolism (ceramide↑/*CGT*↓; uridine↓/uridine phosphorylase↑) are consistent. Together, enrichment of retrograde endocannabinoid and glycerophospholipid metabolism plus the NA-GABA module provides inhibitory/buffering tone that balances inflammation-competent signaling with membrane construction and adhesion-platform reinforcement.

The VTN–integrin–FAK/Src–RhoA axis drives adhesion-first adaptation, elevating GPCR/GNG7 throughput and engaging MAPK13/p38 with potential NF-κB/inflammasome priming; a “downshift” of spliceosome/aa-tRNA/proteasome reallocates resources; the metabolic state is shaped by a VTN-centered PC/LPC/glycerol intermediate hub plus an NA-GABA gene cluster, with ABC transport and GPI anchoring reinforcing membrane trafficking/localization; *ADM* rises during high carbon flux and antioxidant demand and falls with keratinized maturation. The net phenotype is “mobilizable yet controlled” inflammation: pro-inflammatory lipid reservoirs are reduced and inhibitory lipid signals are introduced, while the *GNG7/MAPK13* axis preserves cytokine/chemokine response potential to optimize barrier maintenance and environmental adaptation.

### Metabolic pathways—endocannabinoid system and autophagy

Under *VTN* overexpression, differential metabolites are enriched for autophagy (ko04136), biosynthesis of nucleotide sugars (ko01250), amino sugar and nucleotide sugar metabolism (ko00520), ABC transporters (ko02010), and retrograde endocannabinoid signaling (ko04723), indicating a lipid-centric rewiring that boosts membrane turnover and glycosylation capacity while enhancing cargo trafficking. Autophagy enrichment reflects stress adaptation and PE-dependent membrane remodeling (Mizushima and Komatsu, 2011). sphingolipid and pyrimidine shifts—ceramide↑ with reduced galactosyltransferase, and uridine/deoxyuridine↓ with *UPP1*↑—support apoptosis/stress signaling and uridine-salvage resetting linked to proliferation/energy balance (Lane and Fan, 2015; Hannun and Obeid, 2018).

Multi-omics and the correlation network (Figure 4) place *VTN* at a hub tightly connected to PC/PE/ether lipids and GalCer with reduced arachidonate-linked LPE, while an opposing NA-GABA cluster with CNR1/GABA receptors indicates strengthened inhibitory neuro-lipid coupling; ABC transporters (e.g., *ABCG1*) co-vary with glycerol and nucleotide sugars, aligning lipid export with glycosylation capacity (George, 2023). In the gastrointestinal context, the endocannabinoid system (CB1/CB2) modulates motility, inflammation, secretion, and barrier function (Izzo et al., 2001; Wright et al., 2008; Sharkey and Wiley, 2016; Cuddihey et al., 2022; Camilleri and Zheng, 2023; Aloisio Caruso et al., 2025), and can synergize with VTN signaling to remodel MMP activity and metabolic control (Date et al., 2021; Mun et al., 2022; Jeon et al., 2024; Kim et al., 2024; Lee et al., 2024; Vervaeke and Lamkanfi, 2025). Glycerol feeds PC/PE synthesis, providing substrates for signaling lipids such as N-arachidonoyl-GABA (NA-GABA) while excess lipids are cleared via ABCA7/ABCG1 to maintain membrane homeostasis (Morales et al., 2008; Kotlyarov and Kotlyarova, 2022). NA-GABA, an N-acyl amino acid within the endocannabinoidome, integrates lipid and neurotransmitter signaling—arachidonate moieties modulate K+ channels and the GABA headgroup regulates Cl− flux via GABA receptors—supporting p38 MAPK/ion-channel–linked stress buffering (Henneberry et al., 2002; Meves, 2008; Gibellini and Smith, 2010; Sigel et al., 2011; Burstein, 2014; Battista et al., 2019; Ghit et al., 2021; Yang et al., 2021), with transcript cues such as *CNR1*↑ and *UNC13A*-linked GABA release reinforcing this inhibitory tone (Liu et al., 2019; Mullins et al., 2022). *VTN* orchestrates an endocannabinoid-centered, ABC-supported program in which autophagy and nucleotide-sugar biosynthesis sustain membrane renewal and glycosylation, complementing a transcriptional downshift of splicing/translation to favor barrier maintenance and adaptive remodeling; early PC/PE/ether lipid surges should be interpreted with possible contributions from lipid transfection reagents in mind.

### Tissue crosstalk and ruminant adaptation

The *VTN* gene has relatively low expression in the ruminal and abomasal epithelium of ruminants, while its expression is highest in the liver. The *ADM* gene, however, has higher expression in the ruminal and abomasal epithelium compared to the liver. This reflects the tissue specificity of these two genes. However, the high expression of the *VTN* gene in the liver did not cause a significant change in *ADM* gene expression levels, the results showing significant differences from normal tissue. By comparing with downloaded transcriptomic datasets of the rumen, abomasum, small intestine, and liver to reduce the specificity of this experiment, reflecting the core functional networks regulated by the *VTN*, while exploring the network of *VTN* and *ADM* affecting the actual tissue feed efficiency of ruminants, may provide target genes related to feed efficiency.

Feed efficiency is a comprehensive indicator influenced by multiple factors. Although many related genes have been screened through various omics methods, these genes do not uniformly affect feed efficiency and have their unique regulatory functions. Cultivating breeds that meet diversified needs through personalized gene selection is another option. Liver tissue with high expression of *VTN* and *ADM* genes may influence the gene expression network of high feed efficiency ruminants, where *DLL1*, *CFD*, *CDKN1C*, *CAPS*, *ARHGEF16*, and *SAP25* are commonly upregulated, while *TNNT1* is downregulated. These genes may serve as a specific network of high feed efficiency individuals with high expression of two genes as the axis.

The common intersection genes between the liver and gastrointestinal tract may reflect the core molecular regulatory mechanisms of *VTN* and *ADM* genes in different tissues. Individuals with high expression of *VTN* and *ADM* form a highly coordinated gene network in the digestive tract tissues, exhibiting the advantage of lipid metabolism through differential gene expression regulation.

Among the 29 genes screened, lipid metabolism related genes accounted for the majority. Except for *INSRR* (downregulated), *GRAMD1B* (downregulated) and *LIPG* (upregulated), the expression patterns of the other four lipid metabolism related genes *PLCH2*, *SULT2B1*, *CELSR2* and *FRZB* were different. The first three were upregulated in the rumen, while *FRZB* was only upregulated in the liver. This expression pattern of lipid metabolism related genes revealed the metabolic synergy mechanism among ruminant tissues. The synergistic changes of *INSRR*, *GRAMD1B* and *LIPG* in the two tissues represent the shared basic lipid metabolism pathway, while the other four genes reflect tissue-specific functional differences. This expression pattern reflects the division and cooperation of two key organizations: the rumen is responsible for the initial lipid digestion and processing of microbial fermentation products, while the liver plays a central regulatory role for lipid reprocessing and distribution (Bionaz and Loor, 2012). The synchronous downregulation of *INSRR* and *GRAMD1B* suggests that the insulin signaling pathway and cholesterol sensing function may be attenuated (Naito et al., 2019),the upregulation of *LIPG* enhanced the phospholipid hydrolysis ability of HDL. This expression combination may point to a metabolic state transition: reducing the insulin-dependent lipid synthesis pathway while enhancing the utilization of lipids in circulating lipoproteins, which may be a specific adaptive response in ruminants (Bionaz et al., 2012).

*VTN* and *ADM* genes may form a core molecular regulatory network in different tissues, showing the advantage of lipid metabolism by coordinating the differential expression of these genes. This coordinated regulation mode of lipid metabolism among tissues not only reflects the adaptive evolution of ruminants to herbivorous diet, but also provides a molecular basis for understanding how ruminants adjust lipid metabolism in different physiological states.

### Implications for livestock production

Continuous rumen temperature offers a practical proxy for core heat load and whole-rumen energetic coordination across environments (Vesterdorf et al., 2022), while the rumen epithelium must adapt to fluctuating microbial, thermal, and acid stresses whose community shifts under heat can propagate via blood/lymph to affect performance (Liu et al., 2021; Liu et al., 2022 Y.; Solomon et al., 2022; Huang et al., 2024); cross-species evidence also links *VTN* to feed efficiency, with higher serum VTN in low-RFI pigs (Grubbs et al., 2016), implicating pathways of energy use, immune tone, and hemostasis; This integrated *VTN*-*ADM* framework may offer potential applications across four areas: (1) Monitoring: composite barrier-metabolism indices that could integrate serum/epithelial *VTN* levels, *ADM*-responsive transcripts/metabolites, and rumen temperature; (2) Nutrition: targeted nutritional strategies with PC/PE precursors, butyrate, antioxidants, and microbiome modulation to potentially support VTN-mediated adhesion and ADM-linked redox balance; (3) Health management: possible modulation of endocannabinoid tone via CNR1 pathways when VTN-driven adhesion and MAPK13/GNG7 signaling increase; (4) Breeding and precision selection: multi-omics biomarker panels that could incorporate *VTN*, *ADM*, CTCF-*VTN* regulatory markers, and network genes (e.g., *DLL1*, *CFD*, *CDKN1C*, *CAPS*, *ARHGEF16*, *SAP25*) as candidate phenomic anchors for resilience and feed efficiency. These findings suggest previously unappreciated regulatory layers in rumen epithelial physiology and warrant further investigation to validate potential targets for optimizing animal health and production efficiency.

## Conclusion

This study systematically explored the potential mechanism by which *VTN* gene overexpression drives multicomponent metabolic reprogramming, ultimately remodeling the state of rumen epithelial cells. Specifically, *VTN* overexpression enhances cell adhesion by activating the ECM-receptor interaction and focal adhesion pathways, while concurrently downregulating pathways such as the spliceosome to redistribute cellular resources. Metabolomic analyses further reveal that it triggers lipid reprogramming centered on glycerophospholipid and nucleotide sugar metabolism, working in concert with endocannabinoid signaling (e.g., NA-GABA) to regulate inflammation and homeostasis. Single-cell resolution analysis identifies *ADM* as a metabolic-developmental tuner, highly expressed in early stages to support proliferation and antioxidation, and declining later to facilitate barrier maturation; its expression may be affected by *VTN*, together forming an “adhesion-metabolism-repair” axis. Cross-tissue analysis suggested a lipid metabolic network coordinated by *VTN/ADM* in the liver and gastrointestinal tract, providing a new perspective for understanding nutrient utilization synergy. This research provides a theoretical foundation and biomarkers for genetically and nutritionally targeting improvements in livestock feed efficiency and stress resilience.

## Data Availability

The datasets presented in this study can be found in online repositories. The names of the repository/repositories and accession number(s) can be found in the article/Supplementary Material.
