# Supplementary material for: Potential effects on the signaling network mediated by overexpression of the vitronectin gene in Hu sheep ruminal epithelial cells using multi-omics analysis
Source: Front Genet. 2026 Mar 12;17:1719513. doi: 10.3389/fgene.2026.1719513 (PMC13016589; doi:10.3389/fgene.2026.1719513)
Supplement: Supplementary file 1 [file Supplementaryfile1.docx]

#### CDS region sequence of *VTN* gene

ATGACATCCCTAAGGCCCCTTCTGATGCTGGCCCTGCTGGCGTGGGTTGTTGTGGCTGACCAAGAGTCCTGCAAGGGCCGCTGCACCGAGGGCTTCAACGCCACCAGGAAGTGTCAGTGTGACGAGCTCTGTTCTTACTACCAGAGCTGCTGTGCCGACTTCATGGCCGAGTGCAAGCCCCAAGTGACTCGTGGGGATGTGTTCCATCTGCCAGAAGATGAGTACGGGACCTATGACTACGGCGAGGTGCAGACGGTCAACCGCAGCGTGGAAGCACAGCCCGAGAGCCCCACCCTGGCCCCTGTTCTGCAGGCCGAGATTCCTGTCCAGGCACCGGTTCTCAACCCTGAGAAAGAGGCCCCATCACCTGGGCGGGGAGACTCAGACCCTGGGCTGGGGACCAGTGACCTAGGGACCTCTGAGTCCCCAGCAGAGGAGGAAACGTGCAGTGGGAAACCCTTTGATGCCTTCACCGACCTCAAGAATGGTTCCCTCTTTGCCTTCCGAGGGCTGTACTGCTATGAGCTGGATGAGAAGGCAGTGAGGCCTGGATACCCCAAACTCATCCGAGATGTCTGGGGCATTGAGGGGCCCATTGATGCCGCCTTCACCCGGTTCAACTGTCAGGGAAAGACGTACCTCTTCAAGGGTAGTCAGTACTGGCGCTTTGAGGATGGTGTCCTGGAACCTGACTTCCCCCGAAACATTTCGGATGGCTTCAAGGGTATTCCGGATGACGTGGACGCAGCCTTGGCCCTCCCCGCTCACAGCTACAACGGCAGGGAGCGAGTCTACTTCTTCAAGGGCAACCACTACTGGGAATATGTGTTCCAGCAGCAGCCCAGTCAAGAGGAGTGTGAAGGCAGCTCCCAGCCGGCCGCATTTAAACACTTCGCCCTGATGCAGCGGGACAACTGGGAGGACATCTTCCGACTTCTCTTCTGGGGCGGTTCCTTTGGTGGTGCTGGACAGCCCCAGCTCATCAGCCGCGACTGGTTTGGTCTGCCGGGAAAACTGGATGCGGCCATGGCCGGCCACATCTACATCTCAGGCTCAGCTCCCAGCTCCCCCCGGGCCAAGATGACTAAGTCTGCGCGGCGCCATCGCAAACGTTACCGCTCTCTCCGAAGCCGTGGCCGAGGCCGCGGCCGCGGCCGCAGCCAGAACCCCTACCGGCGATTTCGGTCCACCTGGCTGTCCTGGTTCTCCAGCGAGGAGCTGGGCCTGGGAGCCGACAACTATGATAACTACGAGATGGACTGGCTTGTGCCTGCAACCTGTGAGCCCATCCAGAGTGTCTACTTCTTCTCAGAAGACAAGTACTACCGAGTGAACCTTCGCACGTGGCGGGTGGACTCTGTGATCCCTCCCTACCCACGCTCCATTGCCCAGTACTGGCTGGGCTGCCCAGTCCCTGGCCATGCATAG

#### Primer information

| **Gene** | **Primer（5'-3'）** |
| --- | --- |
| *β-ACTIN* | F:catgttcgagactttcaacactcc |
|  | R:cttcatgaggtagtctgtcaggtc |
| *VTN* | F:ccacatctacatctcaggctcag  R:gccagtccatctcgtagttatcat |
| *COX3* | F:cttcacaggtggctcagtga |
|  | R:cactccatggaccacggaat |
| *DDX5* | F:gtcgatccaggggtagagga |
|  | R:tccaaagctcccattggtgt |
| *MMP2* | F:gccccaaggagagctgtaac |
|  | R:ggctttcgggggaagaagtt |
| *PLOD2* | F:cttcacaggtggctcagtga |
|  | R:cactccatggaccacggaat |
| *PRDX1* | F:ccccacggagatcattgctt |
|  | R:tgccaggtgacagaagtgag |
| *SDC1* | F:cacccacccagtaagaccac |
|  | R:caccttctcagtggcaggag |
| *VIM* | F:ggagcctcatgatgtcttcgg |
|  | R:ggagcagcagaacaagatcct |
| *WARS1* | F:tgccacaggggaggattaca |
|  | R:agcttgtcgtagtcaatgcct |

#### Reads distribution and OPLS-DA


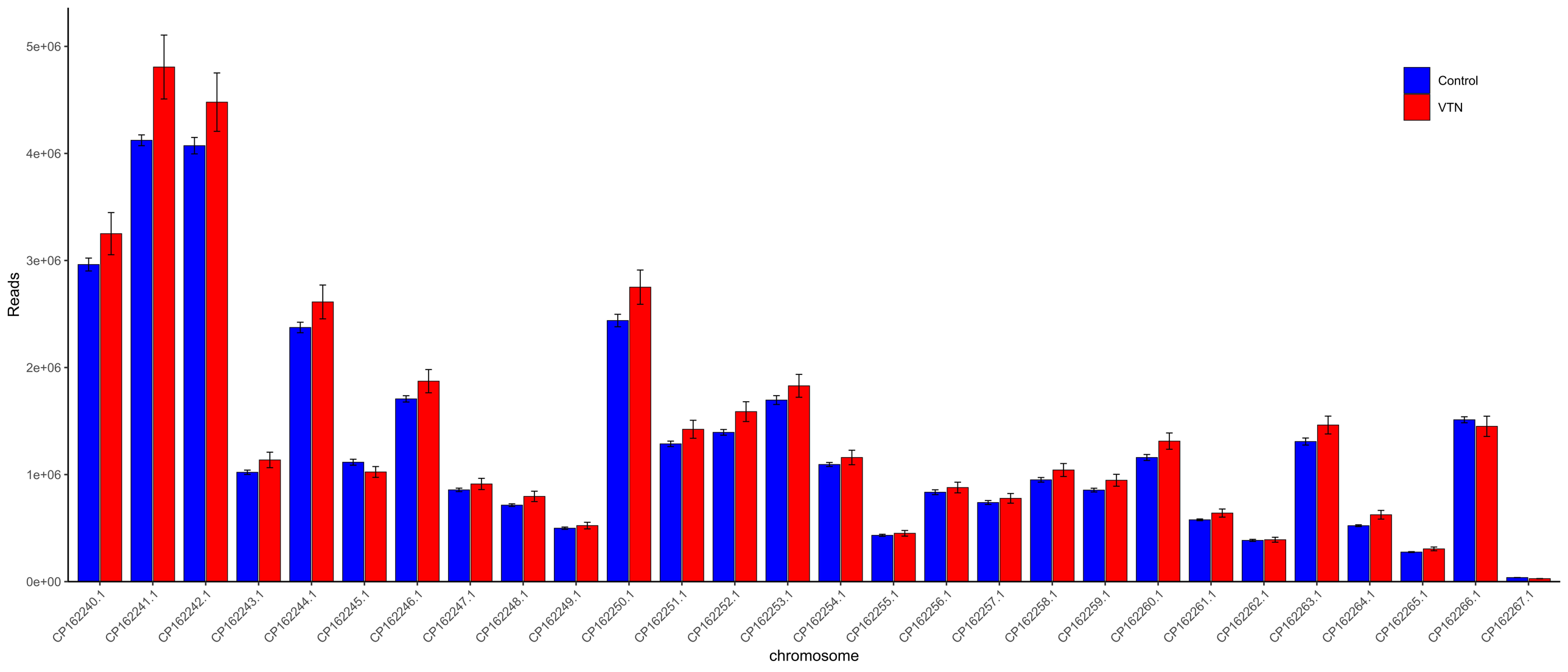


Reads distribution


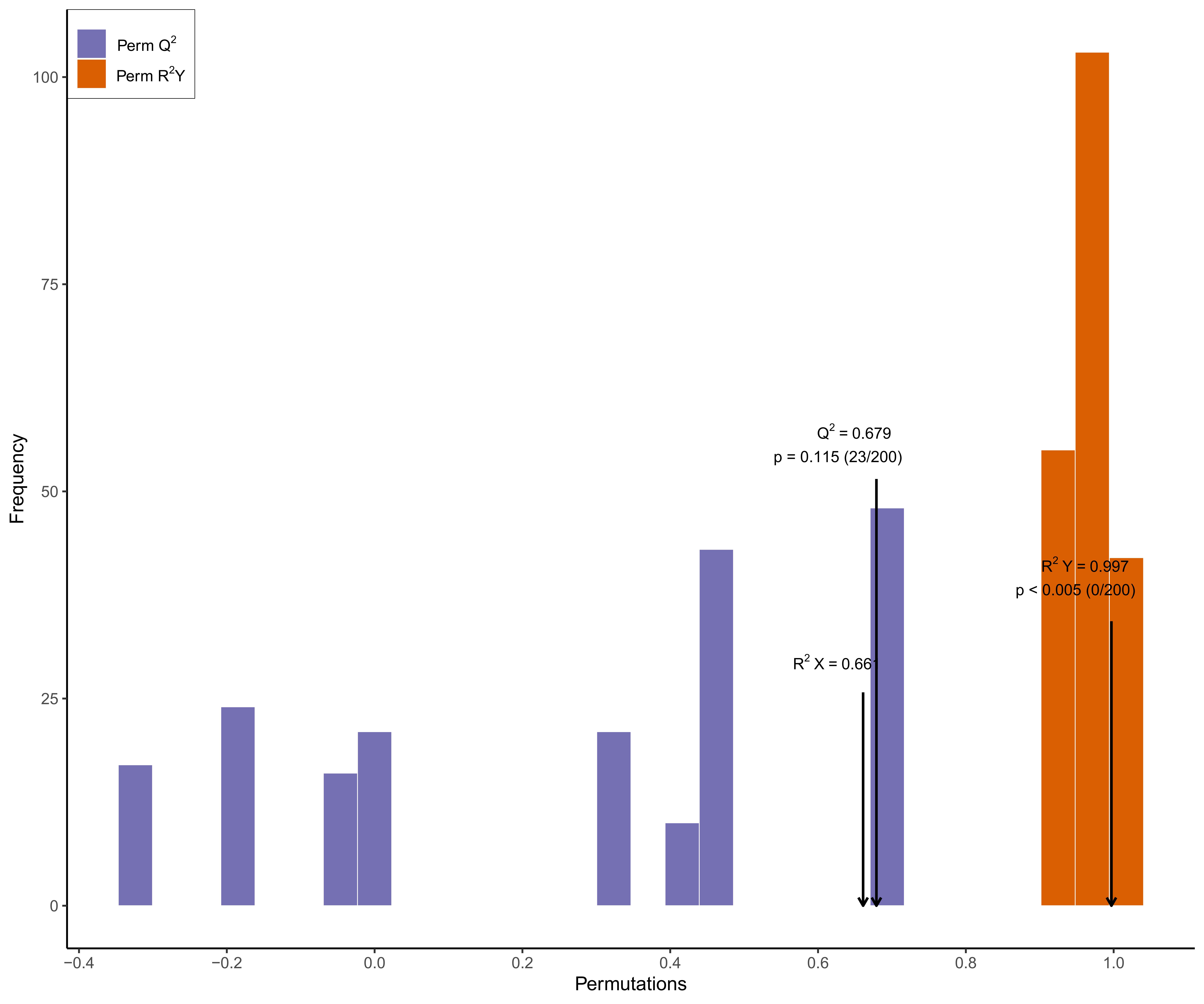


OPLS-DA Model Validation Plot

#### List of differentially expressed genes with *VTN* overexpression

| **Gene id** | **Gene_name** | **Gene_location** | **V_TPM** | **C_TPM** | **log2FoldChange** | **pValue** | **qValue** |
| --- | --- | --- | --- | --- | --- | --- | --- |
| R6Z07_020357 | COX3 | CP162266.1[-]61087409-61087795 | 2368.36084 | 7239.207031 | -1.611942771 | 0 | 0 |
| R6Z07_010525 | VTN | CP162250.1[-]20973303-20976045 | 1822.975464 | 0.0001 | 24.11979181 | 1.14E-52 | 1.63E-50 |
| R6Z07_005319 | SLC2A3 | CP162242.1[-]210253250-210331412 | 642.7976277 | 313.3596397 | 1.03654523 | 1.60E-38 | 1.34E-36 |
| R6Z07_000494 | CCN1 | CP162240.1[+]63351560-63353593 | 635.1422933 | 307.0271707 | 1.048713507 | 5.93E-120 | 4.47E-117 |
| R6Z07_014347 | ADM | CP162254.1[-]62926939-62927888 | 571.04483 | 113.005516 | 2.337210814 | 5.39E-50 | 6.88E-48 |
| R6Z07_007618 | SPP1 | CP162245.1[-]46936795-46963855 | 518.4589437 | 222.6653033 | 1.219352979 | 4.15E-194 | 8.93E-191 |
| R6Z07_002468 | PSAT1 | CP162241.1[-]59091019-59117488 | 435.719472 | 972.9795733 | -1.15900993 | 2.12E-219 | 1.07E-215 |
| R6Z07_018978 | ARL6IP1 | CP162263.1[-]22803328-22809233 | 298.6296083 | 148.5262197 | 1.007639576 | 1.12E-115 | 8.02E-113 |
| R6Z07_002444 | CA9 | CP162241.1[-]53320561-53335052 | 296.0353293 | 122.714152 | 1.270467722 | 1.40E-180 | 2.63E-177 |
| R6Z07_007236 | SH3RF2 | CP162244.1[+]59994389-60141464 | 241.6631267 | 112.8766177 | 1.098250698 | 3.26E-115 | 2.23E-112 |
| R6Z07_016013 | EGLN3 | CP162257.1[-]46758820-46786388 | 202.8868613 | 76.15985367 | 1.413572829 | 6.48E-82 | 2.27E-79 |
| R6Z07_011208 | DDX5 | CP162250.1[-]49637627-49643573 | 170.7940827 | 407.9845277 | -1.256256449 | 2.43E-215 | 9.16E-212 |
| R6Z07_005656 | UPP1 | CP162243.1[-]16816852-16843436 | 137.086558 | 60.84971367 | 1.171764735 | 4.33E-121 | 3.63E-118 |
| R6Z07_016170 | WARS1 | CP162257.1[-]67497549-67517969 | 129.4678547 | 335.632945 | -1.374290396 | 2.17E-201 | 6.53E-198 |
| R6Z07_002191 | CKS2 | CP162241.1[+]24147637-24152114 | 126.804845 | 59.64344567 | 1.088174358 | 5.08E-26 | 2.39E-24 |
| R6Z07_018737 | LOC101108797 | CP162263.1[+]6609844-6614002 | 117.6816253 | 47.61075967 | 1.305529524 | 1.16E-35 | 8.63E-34 |
| R6Z07_019142 | FUS | CP162263.1[+]33635527-33645316 | 111.1822003 | 279.9615277 | -1.332302747 | 3.82E-109 | 2.40E-106 |
| R6Z07_019516 | CDK1 | CP162264.1[+]25362340-25371774 | 109.3513513 | 50.96258567 | 1.101460667 | 4.57E-55 | 7.33E-53 |
| R6Z07_014348 | R6Z07_014348 | CP162254.1[-]62928031-62938347 | 107.2939323 | 15.28633967 | 2.811253593 | 6.58E-133 | 7.08E-130 |
| R6Z07_003985 | SDC1 | CP162242.1[-]28128178-28150170 | 105.378408 | 217.7391713 | -1.047021683 | 7.01E-65 | 1.45E-62 |
| R6Z07_005178 | BHLHE41 | CP162242.1[+]192601575-192604291 | 100.25561 | 46.21522533 | 1.117242845 | 8.70E-66 | 1.90E-63 |
| R6Z07_006659 | CDKN2D | CP162244.1[-]17826583-17828896 | 96.713496 | 40.46075433 | 1.257194009 | 2.63E-57 | 4.45E-55 |
| R6Z07_005701 | ASNS | CP162243.1[-]24231904-24324070 | 89.84080733 | 186.804479 | -1.05608625 | 1.10E-94 | 4.48E-92 |
| R6Z07_006730 | HNRNPM | CP162244.1[+]19806586-19846174 | 86.87529767 | 235.5417127 | -1.438964652 | 1.57E-199 | 3.94E-196 |
| R6Z07_013716 | DBP | CP162253.1[-]57757601-57763163 | 70.224162 | 24.16268033 | 1.539187004 | 6.34E-64 | 1.26E-61 |
| R6Z07_003913 | PTGS1 | CP162242.1[-]13541626-13565406 | 65.82079833 | 140.612432 | -1.095108723 | 3.01E-79 | 9.65E-77 |
| R6Z07_003417 | TINAGL1 | CP162241.1[-]240232785-240286337 | 63.48423 | 23.56639867 | 1.429666951 | 2.25E-175 | 3.76E-172 |
| R6Z07_007249 | LOC101108654 | CP162244.1[+]62267214-62271264 | 60.00459267 | 23.82722267 | 1.332462128 | 6.41E-26 | 2.97E-24 |
| R6Z07_020552 | R6Z07_020552 | CP162266.1[-]84049490-84096305 | 59.141505 | 20.09816967 | 1.557106835 | 6.91E-06 | 4.54E-05 |
| R6Z07_013029 | SLC7A5 | CP162253.1[-]15210914-15245218 | 54.34372833 | 140.713267 | -1.372572906 | 7.37E-108 | 4.44E-105 |
| R6Z07_011992 | DHX9 | CP162251.1[+]77495747-77535785 | 49.08317167 | 104.7024077 | -1.092994236 | 6.35E-121 | 5.03E-118 |
| R6Z07_006961 | MXD3 | CP162244.1[-]41545654-41596893 | 48.33380133 | 23.82566833 | 1.02051578 | 9.23E-25 | 3.91E-23 |
| R6Z07_009230 | SGK1 | CP162247.1[-]64974038-65082843 | 46.46689367 | 102.1525753 | -1.136450467 | 4.92E-59 | 8.52E-57 |
| R6Z07_013776 | ATF5 | CP162253.1[+]58734092-58737852 | 46.36708333 | 106.8284837 | -1.20412348 | 2.81E-44 | 2.95E-42 |
| R6Z07_020557 | LOC121818459 | CP162266.1[-]84837224-84853653 | 43.949711 | 15.140336 | 1.537456456 | 1.13E-18 | 3.24E-17 |
| R6Z07_007154 | EGR1 | CP162244.1[+]52581578-52583902 | 42.440469 | 143.4141797 | -1.756675169 | 1.28E-15 | 2.78E-14 |
| R6Z07_002228 | IARS1 | CP162241.1[+]29051264-29128267 | 41.78157833 | 98.269948 | -1.2338833 | 8.52E-81 | 2.79E-78 |
| R6Z07_021189 | ANGPTL8 | CP162244.1[+]18386733-18388343 | 40.66552733 | 13.39131133 | 1.60250908 | 6.92E-30 | 4.06E-28 |
| R6Z07_001109 | ADAMTS4 | CP162240.1[-]115116856-115123833 | 40.19683067 | 15.85239167 | 1.342381238 | 1.84E-73 | 4.77E-71 |
| R6Z07_009702 | PENK | CP162248.1[-]42047482-42051910 | 40.071285 | 142.4549257 | -1.829864827 | 1.63E-172 | 2.23E-169 |
| R6Z07_006752 | TNFSF9 | CP162244.1[-]20828657-20831161 | 39.88363133 | 17.55985633 | 1.183515729 | 1.96E-26 | 9.46E-25 |
| R6Z07_007944 | MSX1 | CP162245.1[-]114737736-114741043 | 39.49956633 | 15.280554 | 1.370139964 | 1.61E-32 | 1.05E-30 |
| R6Z07_020929 | LOC101116543 | CP162267.1[-]1572448-1599767 | 38.983757 | 118.822423 | -1.607862073 | 8.18E-256 | 6.16E-252 |
| R6Z07_019605 | PLAU | CP162264.1[+]38569720-38574357 | 35.64580167 | 16.84678333 | 1.081259013 | 4.00E-40 | 3.57E-38 |
| R6Z07_015976 | ISLR2 | CP162257.1[-]36801335-36808827 | 33.73781367 | 70.78803267 | -1.069138996 | 3.62E-48 | 4.40E-46 |
| R6Z07_005730 | TSPAN13 | CP162243.1[+]34593707-34630944 | 31.58476533 | 13.46818967 | 1.22967291 | 1.27E-18 | 3.61E-17 |
| R6Z07_003389 | YARS1 | CP162241.1[+]239177613-239208890 | 31.04946833 | 65.370543 | -1.074072116 | 6.25E-64 | 1.26E-61 |
| R6Z07_016924 | TREM1 | CP162259.1[-]21799806-21813718 | 30.70905233 | 66.53376633 | -1.115422712 | 3.11E-23 | 1.19E-21 |
| R6Z07_002307 | CDCA2 | CP162241.1[-]40089910-40129931 | 29.40412067 | 14.324713 | 1.037512113 | 3.50E-64 | 7.13E-62 |
| R6Z07_014961 | SELENOP | CP162255.1[+]34303011-34325313 | 26.93371067 | 12.51991967 | 1.105187699 | 1.41E-13 | 2.47E-12 |
| R6Z07_010592 | SERPINF1 | CP162250.1[+]23939787-23958949 | 26.40346567 | 77.57630433 | -1.554888741 | 1.72E-97 | 7.87E-95 |
| R6Z07_010182 | PSPC1 | CP162249.1[+]45966181-46040166 | 25.96312333 | 54.21202467 | -1.06214894 | 2.28E-26 | 1.09E-24 |
| R6Z07_012493 | GINS1 | CP162252.1[+]60661398-60674374 | 25.90807533 | 53.975843 | -1.058912026 | 1.26E-17 | 3.27E-16 |
| R6Z07_013585 | ZNF428 | CP162253.1[-]54366838-54376292 | 25.77754833 | 53.54125867 | -1.054535997 | 5.79E-19 | 1.69E-17 |
| R6Z07_000112 | SFPQ | CP162240.1[-]10617442-10684442 | 24.48376933 | 62.99157233 | -1.363333141 | 2.40E-61 | 4.52E-59 |
| R6Z07_015972 | CCDC33 | CP162257.1[-]36592601-36617208 | 24.15184667 | 9.447459333 | 1.354135194 | 4.46E-30 | 2.65E-28 |
| R6Z07_018471 | GALNT1 | CP162262.1[-]38349973-38416763 | 23.83240367 | 54.16815033 | -1.184520363 | 3.63E-31 | 2.32E-29 |
| R6Z07_007740 | PDGFRA | CP162245.1[+]80147875-80195173 | 23.54787 | 49.67803433 | -1.077011522 | 7.05E-68 | 1.63E-65 |
| R6Z07_020559 | LOC101112543 | CP162266.1[+]85232839-85274607 | 22.225026 | 9.171879 | 1.276895879 | 4.28E-09 | 4.59E-08 |
| R6Z07_006336 | INSIG1 | CP162243.1[+]127285239-127318400 | 21.75304233 | 9.679877 | 1.168156567 | 1.50E-13 | 2.64E-12 |
| R6Z07_006925 | SNCAIP | CP162244.1[-]34101522-34183154 | 21.40009767 | 42.91921633 | -1.004006353 | 1.09E-45 | 1.20E-43 |
| R6Z07_002534 | VLDLR | CP162241.1[+]71492471-71537598 | 21.072476 | 9.799196667 | 1.104624452 | 2.10E-47 | 2.47E-45 |
| R6Z07_020292 | CCNB3 | CP162266.1[-]56448521-56493558 | 20.20557467 | 10.00289733 | 1.014335447 | 2.30E-44 | 2.44E-42 |
| R6Z07_004873 | CPSF6 | CP162242.1[-]153841613-153862590 | 19.67087467 | 40.86687367 | -1.054870772 | 2.36E-27 | 1.21E-25 |
| R6Z07_000141 | RSPO1 | CP162240.1[-]12927558-12942225 | 19.516564 | 9.009935667 | 1.115110371 | 8.48E-15 | 1.69E-13 |
| R6Z07_014584 | CSTF3 | CP162254.1[-]83452560-83519352 | 19.124951 | 38.47682933 | -1.008533868 | 8.24E-36 | 6.15E-34 |
| R6Z07_001321 | FILIP1L | CP162240.1[-]173900062-174013894 | 18.540613 | 38.083383 | -1.038472697 | 5.48E-46 | 6.08E-44 |
| R6Z07_006061 | AASS | CP162243.1[-]96551675-96663724 | 18.326182 | 9.157276333 | 1.000915789 | 8.00E-34 | 5.45E-32 |
| R6Z07_015684 | DGCR8 | CP162256.1[+]79923630-79935441 | 18.19438567 | 41.78111767 | -1.199357746 | 9.32E-09 | 9.54E-08 |
| R6Z07_013652 | LOC101120688 | CP162253.1[-]56039206-56041665 | 17.73101333 | 2.575683 | 2.783248038 | 5.20E-21 | 1.73E-19 |
| R6Z07_016640 | CSPG5 | CP162258.1[+]54164746-54176623 | 17.54060233 | 7.568871 | 1.212548266 | 4.29E-20 | 1.35E-18 |
| R6Z07_003554 | WNT4 | CP162241.1[+]248774942-248801329 | 17.476866 | 36.78196333 | -1.073551989 | 1.45E-20 | 4.74E-19 |
| R6Z07_000437 | CTH | CP162240.1[+]46688233-46710606 | 16.81676467 | 36.325994 | -1.111102099 | 4.15E-21 | 1.38E-19 |
| R6Z07_002958 | GCA | CP162241.1[-]151548615-151563671 | 16.73836 | 7.645300333 | 1.1305131 | 6.17E-10 | 7.40E-09 |
| R6Z07_019234 | LOC101117299 | CP162263.1[+]40843480-40865756 | 16.326907 | 8.032058667 | 1.023409798 | 8.03E-30 | 4.69E-28 |
| R6Z07_007047 | CFD | CP162244.1[+]46174681-46176838 | 16.31145033 | 5.857012333 | 1.477648228 | 5.72E-16 | 1.29E-14 |
| R6Z07_006097 | GARIN1A | CP162243.1[+]102738485-102789175 | 15.95236033 | 6.879501667 | 1.213393934 | 2.20E-21 | 7.55E-20 |
| R6Z07_000913 | LOC101103771 | CP162240.1[-]106148973-106149708 | 15.5492 | 6.338331667 | 1.294665297 | 1.73E-05 | 0.00010624 |
| R6Z07_011286 | NDUFAF8 | CP162250.1[-]52306673-52308335 | 15.30623167 | 31.27397367 | -1.030843399 | 0.000160492 | 0.00080092 |
| R6Z07_010899 | DLX3 | CP162250.1[+]37592369-37595813 | 15.25898667 | 32.50664967 | -1.091075713 | 1.38E-14 | 2.69E-13 |
| R6Z07_004945 | INHBC | CP162242.1[-]165320895-165337401 | 14.78728933 | 39.70413967 | -1.424931819 | 9.34E-74 | 2.47E-71 |
| R6Z07_002540 | SLC1A1 | CP162241.1[+]73371833-73444593 | 12.673453 | 38.26858633 | -1.594350954 | 3.19E-46 | 3.59E-44 |
| R6Z07_006856 | GNG7 | CP162244.1[+]23890382-23893967 | 12.10012333 | 5.555476667 | 1.123039146 | 0.002845686 | 0.010246919 |
| R6Z07_016875 | MAPK13 | CP162259.1[+]16790650-16799077 | 12.00557833 | 4.589354 | 1.387341904 | 1.84E-21 | 6.38E-20 |
| R6Z07_000188 | CTPS1 | CP162240.1[+]16003551-16035662 | 11.783077 | 23.99136433 | -1.025798873 | 3.11E-18 | 8.67E-17 |
| R6Z07_016089 | GPR68 | CP162257.1[-]58434118-58472748 | 11.71730867 | 25.32364267 | -1.111843706 | 3.88E-21 | 1.30E-19 |
| R6Z07_005558 | SULT4A1 | CP162242.1[-]225509111-225535499 | 11.52278167 | 5.428329333 | 1.085908878 | 1.30E-09 | 1.49E-08 |
| R6Z07_005228 | MGP | CP162242.1[+]203864834-203868100 | 11.010969 | 36.813657 | -1.741299635 | 1.40E-11 | 2.05E-10 |
| R6Z07_021145 | FBLN7 | CP162242.1[+]106484030-106517584 | 11.00533533 | 4.025449333 | 1.450981369 | 1.73E-15 | 3.68E-14 |
| R6Z07_017604 | ADAMTS15 | CP162260.1[+]37334139-37355901 | 10.929925 | 31.401131 | -1.522533021 | 9.88E-71 | 2.40E-68 |
| R6Z07_008465 | CHAC1 | CP162246.1[+]42982876-42984408 | 10.66590433 | 34.40365333 | -1.68955548 | 6.39E-26 | 2.97E-24 |
| R6Z07_008540 | R6Z07_008540 | CP162246.1[+]51833729-51841109 | 10.626074 | 4.572768 | 1.216469034 | 1.64E-22 | 5.98E-21 |
| R6Z07_018802 | FAHD1 | CP162263.1[+]7768778-7769443 | 10.410844 | 21.655318 | -1.056634326 | 2.71E-07 | 2.24E-06 |
| R6Z07_000106 | GJB3 | CP162240.1[+]10328402-10329214 | 10.340457 | 22.21521733 | -1.103248308 | 1.28E-10 | 1.68E-09 |
| R6Z07_010636 | MYBBP1A | CP162250.1[-]26091168-26105752 | 9.865509667 | 21.811898 | -1.144649824 | 9.39E-41 | 8.68E-39 |
| R6Z07_004395 | FAM136A | CP162242.1[-]92581414-92584580 | 9.753703667 | 22.53893933 | -1.208397578 | 2.28E-07 | 1.91E-06 |
| R6Z07_000611 | R6Z07_000611 | CP162240.1[+]86978992-86995578 | 9.740808667 | 3.940773667 | 1.305562655 | 9.54E-07 | 7.16E-06 |
| R6Z07_014894 | DEPDC1B | CP162255.1[+]20799946-20946725 | 9.481322333 | 4.707350667 | 1.010172953 | 1.87E-16 | 4.43E-15 |
| R6Z07_018629 | ZBTB7C | CP162262.1[-]64666061-64773655 | 9.466496333 | 22.02648833 | -1.218337035 | 1.38E-29 | 7.82E-28 |
| R6Z07_012846 | NCOA5 | CP162252.1[-]82475755-82493560 | 9.341494333 | 23.15281733 | -1.3094625 | 6.44E-29 | 3.54E-27 |
| R6Z07_012205 | R6Z07_012205 | CP162252.1[-]24099012-24118980 | 9.235234667 | 4.266751 | 1.114010701 | 0.000248322 | 0.001189579 |
| R6Z07_017303 | CAP2 | CP162259.1[-]46009784-46196666 | 9.160896 | 19.42758567 | -1.084546007 | 4.26E-17 | 1.06E-15 |
| R6Z07_000320 | RAB3B | CP162240.1[-]26890700-26978909 | 8.95447 | 3.070910333 | 1.543941656 | 9.24E-09 | 9.47E-08 |
| R6Z07_011032 | KRT14 | CP162250.1[-]42758855-42762896 | 8.851967667 | 3.663664333 | 1.272710852 | 5.77E-15 | 1.17E-13 |
| R6Z07_018096 | SFRP5 | CP162261.1[-]20707987-20712111 | 8.792962333 | 2.463985 | 1.835355815 | 1.66E-15 | 3.56E-14 |
| R6Z07_019433 | TUBB6 | CP162264.1[-]11498836-11500174 | 8.661058 | 20.41284933 | -1.236862401 | 6.18E-15 | 1.25E-13 |
| R6Z07_002591 | MLLT3 | CP162241.1[-]89318357-89592975 | 8.620708 | 4.306662 | 1.001236259 | 2.41E-09 | 2.67E-08 |
| R6Z07_013721 | FGF21 | CP162253.1[+]57861267-57862715 | 8.495581333 | 19.96152867 | -1.232437631 | 4.17E-10 | 5.12E-09 |
| R6Z07_017797 | FOSL1 | CP162260.1[-]45430853-45436535 | 8.448114333 | 17.71557833 | -1.068317299 | 8.67E-07 | 6.57E-06 |
| R6Z07_004990 | PYM1 | CP162242.1[+]166734305-166750979 | 8.382478667 | 19.42284667 | -1.21230585 | 1.16E-08 | 1.17E-07 |
| R6Z07_001559 | ST6GAL1 | CP162240.1[-]209922952-209952939 | 8.351737333 | 22.65538733 | -1.439705912 | 9.84E-24 | 3.89E-22 |
| R6Z07_009754 | R6Z07_009754 | CP162248.1[+]51745847-51850740 | 8.191396667 | 17.67194233 | -1.109279253 | 3.70E-07 | 2.98E-06 |
| R6Z07_004669 | HOXC8 | CP162242.1[-]135586250-135588357 | 8.162963333 | 18.80624433 | -1.204046885 | 3.05E-10 | 3.79E-09 |
| R6Z07_015700 | NDN | CP162257.1[-]1790375-1791444 | 8.050591 | 18.25023333 | -1.180748308 | 2.34E-11 | 3.34E-10 |
| R6Z07_013732 | LHB | CP162253.1[-]58046669-58047637 | 7.974379 | 3.432475333 | 1.216122821 | 6.62E-05 | 0.00036026 |
| R6Z07_018590 | IMPA2 | CP162262.1[+]59903740-59924888 | 7.92114 | 2.808581333 | 1.495866494 | 1.10E-09 | 1.28E-08 |
| R6Z07_006646 | ICAM5 | CP162244.1[+]17583312-17592621 | 7.742635667 | 3.593810333 | 1.107310486 | 1.84E-24 | 7.68E-23 |
| R6Z07_003469 | MAP3K6 | CP162241.1[+]243997644-244010818 | 7.494206 | 16.67888533 | -1.154175335 | 7.13E-32 | 4.59E-30 |
| R6Z07_017266 | DCDC2 | CP162259.1[+]39270826-39408126 | 7.459390333 | 14.936067 | -1.001670677 | 3.74E-09 | 4.02E-08 |
| R6Z07_018021 | IFIT2 | CP162261.1[+]12817945-12824144 | 7.372448 | 16.895843 | -1.196452687 | 8.95E-15 | 1.78E-13 |
| R6Z07_017255 | RIPOR2 | CP162259.1[+]38732573-38947346 | 7.136357333 | 3.501452667 | 1.027234271 | 7.40E-16 | 1.66E-14 |
| R6Z07_018281 | DMBT1 | CP162261.1[+]43941503-44007631 | 7.010929333 | 1.999596667 | 1.809896666 | 6.78E-27 | 3.38E-25 |
| R6Z07_019270 | SAP25 | CP162263.1[+]42343828-42345308 | 6.821446 | 3.017569 | 1.176690832 | 2.02E-07 | 1.71E-06 |
| R6Z07_005737 | HDAC9 | CP162243.1[+]36029679-37010012 | 6.775331 | 1.658620333 | 2.030307745 | 7.49E-38 | 6.04E-36 |
| R6Z07_014488 | PDE2A | CP162254.1[-]71716142-71843405 | 6.652438 | 2.461603333 | 1.434284857 | 6.41E-26 | 2.97E-24 |
| R6Z07_005693 | PDK4 | CP162243.1[-]21996625-22036659 | 6.629595 | 3.059882667 | 1.115444406 | 6.87E-07 | 5.31E-06 |
| R6Z07_000583 | LOC101113636 | CP162240.1[+]78746221-78767002 | 6.428190667 | 2.945974333 | 1.12566786 | 2.22E-11 | 3.18E-10 |
| R6Z07_016328 | LOC101113538 | CP162258.1[+]14694624-14700797 | 6.380188667 | 2.897582667 | 1.138749265 | 0.000123565 | 0.000634504 |
| R6Z07_010585 | SLC43A2 | CP162250.1[-]23794962-23833566 | 6.372002667 | 12.76784433 | -1.002696192 | 9.03E-14 | 1.62E-12 |
| R6Z07_013743 | CD37 | CP162253.1[+]58290309-58295230 | 6.278290333 | 3.081031667 | 1.026958236 | 1.77E-07 | 1.51E-06 |
| R6Z07_003424 | SDC3 | CP162241.1[+]240745075-240779612 | 6.276499333 | 12.98840733 | -1.049192498 | 1.88E-08 | 1.84E-07 |
| R6Z07_015330 | ATP6V0A2 | CP162256.1[-]59113349-59153366 | 6.252058667 | 13.46739533 | -1.107067632 | 7.54E-18 | 2.03E-16 |
| R6Z07_001486 | TFRC | CP162240.1[-]200398660-200442981 | 6.249111 | 17.16043033 | -1.457362861 | 3.56E-35 | 2.60E-33 |
| R6Z07_003032 | CXCR4 | CP162241.1[+]178840551-178843606 | 6.242651 | 1.821881 | 1.776730084 | 2.07E-11 | 2.97E-10 |
| R6Z07_011193 | FTSJ3 | CP162250.1[-]49017278-49024157 | 6.092992 | 13.34083867 | -1.130626613 | 4.16E-18 | 1.14E-16 |
| R6Z07_013708 | GRWD1 | CP162253.1[+]57610630-57622157 | 6.060176 | 12.374738 | -1.029966382 | 3.13E-09 | 3.42E-08 |
| R6Z07_006638 | PPAN | CP162244.1[+]17420813-17424600 | 5.977623667 | 12.29271533 | -1.04015965 | 2.24E-08 | 2.17E-07 |
| R6Z07_015880 | ARNT2 | CP162257.1[+]28671252-28857106 | 5.946384667 | 13.16719267 | -1.146863088 | 1.20E-16 | 2.92E-15 |
| R6Z07_005689 | PON1 | CP162243.1[-]21671978-21704918 | 5.929836333 | 2.874001333 | 1.044931555 | 7.75E-06 | 5.04E-05 |
| R6Z07_013011 | C14H16orf74 | CP162253.1[-]13605612-13626955 | 5.923756 | 13.111055 | -1.146199657 | 0.00465805 | 0.015643823 |
| R6Z07_003705 | R6Z07_003705 | CP162242.1[-]1172656-1176069 | 5.850424333 | 11.711132 | -1.001267361 | 0.000512482 | 0.0022709 |
| R6Z07_017975 | IFITM5 | CP162260.1[+]52503956-52505001 | 5.822926667 | 13.68863867 | -1.233162623 | 6.17E-05 | 0.000338292 |
| R6Z07_018844 | PRSS33 | CP162263.1[+]8611233-8631961 | 5.812186 | 2.064974333 | 1.492957023 | 2.52E-13 | 4.31E-12 |
| R6Z07_003071 | TMEM185B | CP162241.1[-]189601336-189606263 | 5.733968 | 12.62235733 | -1.138375613 | 7.30E-11 | 9.94E-10 |
| R6Z07_018452 | SERPINB2 | CP162262.1[-]29313489-29325704 | 5.716658 | 11.68396333 | -1.031285847 | 1.34E-07 | 1.16E-06 |
| R6Z07_009875 | R6Z07_009875 | CP162248.1[-]82667903-82668817 | 5.696107667 | 1.500211667 | 1.924810349 | 0.00038983 | 0.001781913 |
| R6Z07_018481 | DTNA | CP162262.1[-]39101330-39245859 | 5.644990667 | 2.774845333 | 1.024563836 | 3.69E-10 | 4.56E-09 |
| R6Z07_015050 | ANKRD33B | CP162255.1[-]65141155-65236374 | 5.509548 | 2.636745333 | 1.063175729 | 2.05E-07 | 1.73E-06 |
| R6Z07_012150 | PAK5 | CP162252.1[-]9608397-9722577 | 5.474170667 | 2.124811667 | 1.365305441 | 9.65E-16 | 2.12E-14 |
| R6Z07_014963 | GHR | CP162255.1[-]34423258-34720578 | 5.372683333 | 11.57921367 | -1.107822572 | 4.24E-14 | 7.82E-13 |
| R6Z07_009037 | DCBLD1 | CP162247.1[-]25653506-25732579 | 5.324177667 | 11.22527867 | -1.076120641 | 1.77E-16 | 4.19E-15 |
| R6Z07_003376 | ZNF362 | CP162241.1[-]238719940-238738935 | 5.288748333 | 11.493323 | -1.119797746 | 3.10E-08 | 2.95E-07 |
| R6Z07_011888 | ISG15 | CP162251.1[-]64539920-64540839 | 4.968524667 | 16.585309 | -1.739016459 | 2.89E-08 | 2.76E-07 |
| R6Z07_007998 | CPZ | CP162245.1[+]125144386-125166471 | 4.960556 | 2.088984667 | 1.24769993 | 1.26E-10 | 1.66E-09 |
| R6Z07_002957 | KCNH7 | CP162241.1[+]151006508-151545726 | 4.952771333 | 2.302700333 | 1.104909339 | 6.57E-14 | 1.19E-12 |
| R6Z07_010856 | RASD1 | CP162250.1[+]35768316-35769371 | 4.855368333 | 2.185636667 | 1.151527152 | 3.34E-05 | 0.00019364 |
| R6Z07_003302 | KCNE4 | CP162241.1[+]229435573-229436783 | 4.819971333 | 1.602967667 | 1.588279241 | 1.81E-05 | 0.000110542 |
| R6Z07_019988 | XG | CP162266.1[+]1370561-1392168 | 4.767362 | 10.89480333 | -1.192377071 | 2.82E-05 | 0.000165953 |
| R6Z07_005393 | KCNA5 | CP162242.1[-]215996199-215997995 | 4.750961667 | 2.026154333 | 1.229475497 | 1.95E-10 | 2.51E-09 |
| R6Z07_006633 | COL5A3 | CP162244.1[-]17298219-17343118 | 4.729222 | 1.952061 | 1.27660473 | 1.71E-27 | 8.87E-26 |
| R6Z07_006714 | PRR36 | CP162244.1[-]19383149-19394129 | 4.677385667 | 1.965372333 | 1.250899737 | 2.17E-20 | 6.98E-19 |
| R6Z07_001920 | WDR4 | CP162240.1[-]272909657-272927618 | 4.561995333 | 9.368387667 | -1.038135805 | 7.55E-07 | 5.79E-06 |
| R6Z07_014541 | LRRC32 | CP162254.1[-]75708195-75715069 | 4.496286333 | 1.153824 | 1.962310735 | 2.28E-18 | 6.39E-17 |
| R6Z07_013720 | FUT1 | CP162253.1[-]57857202-57858907 | 4.481781 | 9.669007 | -1.10929558 | 2.81E-07 | 2.31E-06 |
| R6Z07_001501 | NCBP2AS2 | CP162240.1[+]201129221-201133323 | 4.258384 | 9.320323667 | -1.130074005 | 0.002738745 | 0.009897321 |
| R6Z07_007056 | ABCA7 | CP162244.1[+]46303699-46320610 | 4.242416667 | 1.912849 | 1.149163329 | 2.39E-22 | 8.67E-21 |
| R6Z07_001934 | PWP2 | CP162240.1[+]273967160-273982837 | 4.226978 | 8.683787667 | -1.038697846 | 6.32E-15 | 1.27E-13 |
| R6Z07_002216 | WNK2 | CP162241.1[-]28267673-28422675 | 4.198846667 | 1.981338 | 1.083518092 | 2.33E-20 | 7.48E-19 |
| R6Z07_010576 | LIAT1 | CP162250.1[-]23389834-23392808 | 4.195041 | 8.394888667 | -1.000826281 | 0.001002705 | 0.004106212 |
| R6Z07_000793 | NUDT17 | CP162240.1[+]101472807-101474972 | 4.183203667 | 12.236354 | -1.548493605 | 4.12E-11 | 5.74E-10 |
| R6Z07_013582 | LOC101106541 | CP162253.1[+]54322211-54346041 | 4.176842667 | 9.424932667 | -1.17406951 | 0.0001297 | 0.000661051 |
| R6Z07_013789 | MYBPC2 | CP162253.1[+]59050534-59075719 | 4.161764667 | 8.497594 | -1.029859028 | 4.01E-15 | 8.28E-14 |
| R6Z07_003398 | FAM229A | CP162241.1[+]239550095-239550736 | 4.089299333 | 1.550325333 | 1.399282676 | 0.004023574 | 0.013802178 |
| R6Z07_012863 | SULF2 | CP162252.1[-]83893701-83990172 | 3.977389667 | 8.355628 | -1.070926353 | 1.69E-11 | 2.46E-10 |
| R6Z07_003066 | TMEM37 | CP162241.1[+]188892189-188897760 | 3.944776667 | 1.401147 | 1.493335302 | 0.000239742 | 0.001152507 |
| R6Z07_010730 | ATP1B2 | CP162250.1[+]28374189-28378458 | 3.866070667 | 1.575824333 | 1.294761291 | 0.000413107 | 0.001873533 |
| R6Z07_020614 | SLITRK4 | CP162266.1[+]94372971-94398661 | 3.814676 | 7.643799 | -1.002729315 | 3.22E-11 | 4.53E-10 |
| R6Z07_016227 | LOC121817141 | CP162257.1[-]71496539-71498395 | 3.809847333 | 7.627634 | -1.001502432 | 6.94E-08 | 6.26E-07 |
| R6Z07_017909 | CDKN1C | CP162260.1[+]50190281-50205883 | 3.655948333 | 1.540583333 | 1.246768959 | 1.97E-06 | 1.41E-05 |
| R6Z07_001911 | ABCG1 | CP162240.1[+]272309438-272372569 | 3.617848333 | 1.758654333 | 1.040659983 | 1.66E-06 | 1.20E-05 |
| R6Z07_016868 | ARMC12 | CP162259.1[+]16469551-16479052 | 3.560812333 | 1.754370333 | 1.021253082 | 0.000544656 | 0.002398571 |
| R6Z07_021064 | LOC101105704 | CP162240.1[-]108965975-108970004 | 3.541501 | 8.065595333 | -1.187420075 | 1.43E-06 | 1.05E-05 |
| R6Z07_019197 | ELN | CP162263.1[-]39598557-39629979 | 3.532018 | 1.583821667 | 1.157082794 | 3.52E-08 | 3.32E-07 |
| R6Z07_009331 | PPP1R14C | CP162247.1[+]81678245-81763494 | 3.330108 | 9.687897333 | -1.54061461 | 2.65E-05 | 0.000156387 |
| R6Z07_018154 | PITX3 | CP162261.1[-]24603933-24605523 | 3.318641 | 1.575216667 | 1.075042291 | 0.000963667 | 0.003966698 |
| R6Z07_006263 | ZNF783 | CP162243.1[+]122051809-122069247 | 3.305381 | 6.885655 | -1.058777322 | 1.74E-08 | 1.71E-07 |
| R6Z07_003027 | HNMT | CP162241.1[-]176729276-176770626 | 3.237492667 | 7.823911 | -1.273013034 | 7.38E-06 | 4.83E-05 |
| R6Z07_004403 | ATP6V1B1 | CP162242.1[+]93243274-93268551 | 3.084846667 | 6.693631 | -1.11759024 | 2.72E-06 | 1.90E-05 |
| R6Z07_015212 | SLC7A11 | CP162256.1[+]26586292-26670390 | 3.049653667 | 8.288656333 | -1.442492834 | 2.12E-11 | 3.05E-10 |
| R6Z07_008670 | DLAT | CP162246.1[+]71908055-71908483 | 3.020511 | 7.01933 | -1.21654069 | 0.005225865 | 0.017250852 |
| R6Z07_014021 | LOC101110171 | CP162253.1[-]67622366-67647279 | 2.861730667 | 6.129019667 | -1.098768436 | 1.06E-08 | 1.08E-07 |
| R6Z07_017835 | SYT12 | CP162260.1[+]46393338-46425677 | 2.852026 | 6.432251667 | -1.17333672 | 7.67E-06 | 4.99E-05 |
| R6Z07_007821 | GC | CP162245.1[-]97487990-97525449 | 2.819984 | 6.780638667 | -1.26573419 | 4.99E-08 | 4.59E-07 |
| R6Z07_002334 | EGR3 | CP162241.1[+]43404708-43407167 | 2.782159 | 8.547346 | -1.619271652 | 8.76E-12 | 1.31E-10 |
| R6Z07_007180 | SLC4A9 | CP162244.1[+]54306922-54319148 | 2.734016 | 0.591712667 | 2.208053002 | 1.06E-18 | 3.06E-17 |
| R6Z07_012807 | LOC101110259 | CP162252.1[+]81379098-81400651 | 2.685176333 | 0.979721333 | 1.454573472 | 0.002876658 | 0.010346079 |
| R6Z07_015951 | C18H15orf39 | CP162257.1[-]35784276-35793341 | 2.665760667 | 6.001438 | -1.170760965 | 3.08E-11 | 4.35E-10 |
| R6Z07_007911 | CDS1 | CP162245.1[+]109879339-109972245 | 2.660809333 | 1.259819 | 1.07864866 | 0.00017651 | 0.000874769 |
| R6Z07_007710 | GABRA4 | CP162245.1[-]75917812-75994518 | 2.631705667 | 1.294179333 | 1.023960601 | 0.000108972 | 0.000567696 |
| R6Z07_018475 | LOC121817701 | CP162262.1[-]38713753-38724537 | 2.628919667 | 5.347541 | -1.024405581 | 2.24E-07 | 1.88E-06 |
| R6Z07_013359 | SBSN | CP162253.1[-]47775151-47779658 | 2.562138 | 1.082470667 | 1.243020253 | 8.94E-06 | 5.75E-05 |
| R6Z07_009576 | PTP4A3 | CP162248.1[-]20996123-21002224 | 2.532125 | 6.469853667 | -1.353384456 | 0.000624855 | 0.00270519 |
| R6Z07_001017 | INSRR | CP162240.1[-]109189214-109218793 | 2.513127333 | 1.040779333 | 1.271819551 | 6.72E-10 | 8.02E-09 |
| R6Z07_017211 | LOC101106041 | CP162259.1[+]37298669-37308897 | 2.510363667 | 1.066324 | 1.235250513 | 0.000410365 | 0.001863338 |
| R6Z07_017922 | TNNT3 | CP162260.1[-]51055883-51071729 | 2.478865 | 0.352876667 | 2.812443761 | 1.19E-07 | 1.03E-06 |
| R6Z07_007711 | GABRB1 | CP162245.1[+]76035065-76477060 | 2.452981667 | 1.071067667 | 1.195486824 | 0.000238117 | 0.001145058 |
| R6Z07_018887 | SRL | CP162263.1[-]9820067-9854705 | 2.447431 | 0.850386 | 1.525078436 | 4.42E-09 | 4.72E-08 |
| R6Z07_015102 | TPPP | CP162255.1[+]73966512-73985662 | 2.397915 | 1.016662 | 1.2379404 | 0.000458291 | 0.002056166 |
| R6Z07_019708 | ZNF239 | CP162264.1[+]54142523-54143731 | 2.386730667 | 6.100891 | -1.353984182 | 9.85E-08 | 8.68E-07 |
| R6Z07_003109 | TMEFF2 | CP162241.1[-]198427806-198716893 | 2.385265333 | 0.988462333 | 1.270891862 | 0.00033931 | 0.001574392 |
| R6Z07_013653 | HIF3A | CP162253.1[+]56068534-56097379 | 2.379849 | 0.776857333 | 1.615148455 | 6.86E-09 | 7.14E-08 |
| R6Z07_020555 | R6Z07_020555 | CP162266.1[-]84319336-84364311 | 2.379551 | 0.472647 | 2.331854372 | 0.001155882 | 0.004661074 |
| R6Z07_003380 | AZIN2 | CP162241.1[-]238885248-238924202 | 2.346193333 | 1.064463333 | 1.140195645 | 0.000566822 | 0.002482482 |
| R6Z07_000107 | GJA4 | CP162240.1[+]10339558-10341983 | 2.341736 | 0.875581 | 1.419265886 | 2.79E-05 | 0.000164123 |
| R6Z07_003194 | LOC121818518 | CP162241.1[-]213783136-213805826 | 2.340245 | 1.070227667 | 1.128741843 | 0.000216501 | 0.00105016 |
| R6Z07_014776 | SLIT3 | CP162255.1[-]2672871-3399313 | 2.326305667 | 1.075375667 | 1.113199941 | 8.36E-11 | 1.13E-09 |
| R6Z07_016166 | DEGS2 | CP162257.1[-]67349553-67391618 | 2.304632667 | 0.533501667 | 2.110972136 | 1.57E-09 | 1.79E-08 |
| R6Z07_006060 | PTPRZ1 | CP162243.1[+]96337643-96544279 | 2.302181333 | 4.766755667 | -1.050006205 | 9.34E-13 | 1.50E-11 |
| R6Z07_005935 | CPVL | CP162243.1[+]77042964-77149092 | 2.297609333 | 4.898395 | -1.092175601 | 6.98E-05 | 0.000378781 |
| R6Z07_021078 | VWA5B2 | CP162240.1[-]212658813-212670190 | 2.253747667 | 1.037527667 | 1.11917619 | 3.59E-09 | 3.87E-08 |
| R6Z07_017056 | AGER | CP162259.1[+]33045269-33048198 | 2.225423667 | 1.018344 | 1.127855025 | 0.001207716 | 0.004839217 |
| R6Z07_021390 | VWA7 | CP162259.1[+]33393943-33402095 | 2.218759333 | 1.042178333 | 1.090151021 | 2.47E-06 | 1.74E-05 |
| R6Z07_004685 | AMHR2 | CP162242.1[-]136160483-136166282 | 2.207946667 | 1.019793667 | 1.114428041 | 0.000241854 | 0.001160808 |
| R6Z07_004658 | GPR84 | CP162242.1[+]135277742-135278932 | 2.205912667 | 6.873722333 | -1.6397159 | 4.61E-10 | 5.61E-09 |
| R6Z07_009890 | MATN2 | CP162248.1[-]84601411-84750502 | 2.180828 | 7.378671 | -1.758485 | 9.11E-24 | 3.61E-22 |
| R6Z07_020228 | ARHGEF9 | CP162266.1[-]47346427-47673716 | 2.146801333 | 4.583785667 | -1.094350897 | 3.84E-05 | 0.000221081 |
| R6Z07_008110 | F2RL1 | CP162246.1[+]15505668-15537105 | 2.109659 | 4.955353667 | -1.231978203 | 2.29E-06 | 1.62E-05 |
| R6Z07_016070 | LCMT2 | CP162257.1[-]57522951-57525005 | 2.054288333 | 4.119199333 | -1.003725254 | 0.000184009 | 0.000908049 |
| R6Z07_007499 | LOC101116481 | CP162245.1[-]18345066-18379932 | 2.040233 | 4.934947333 | -1.274300767 | 0.000365651 | 0.001680056 |
| R6Z07_017316 | CD83 | CP162259.1[-]48838418-48869571 | 2.036149 | 0.969690667 | 1.070246635 | 0.00621531 | 0.020012794 |
| R6Z07_007909 | NKX6-1 | CP162245.1[-]109767537-109772555 | 2.005291333 | 0.916645667 | 1.129375783 | 0.003514188 | 0.012252894 |
| R6Z07_008795 | R6Z07_008795 | CP162246.1[-]87065518-87068637 | 1.980875 | 0.077538333 | 4.675084309 | 0.002214785 | 0.008204562 |
| R6Z07_009669 | CLXN | CP162248.1[-]38318044-38331728 | 1.968102667 | 0.772765667 | 1.348702578 | 0.002296354 | 0.008477546 |
| R6Z07_013017 | FOXF1 | CP162253.1[+]14251859-14255345 | 1.954662667 | 0.513009667 | 1.929861734 | 0.000153217 | 0.000768175 |
| R6Z07_000191 | FOXO6 | CP162240.1[+]16392188-16412878 | 1.927698667 | 0.654122333 | 1.559247173 | 1.04E-05 | 6.60E-05 |
| R6Z07_000741 | TENT5C | CP162240.1[+]95814953-95844213 | 1.907120333 | 0.657669333 | 1.535961571 | 9.23E-05 | 0.00048688 |
| R6Z07_001398 | ZBTB20 | CP162240.1[-]188803614-188846381 | 1.879733 | 0.575277 | 1.708199057 | 2.66E-08 | 2.55E-07 |
| R6Z07_003059 | LOC101118571 | CP162241.1[+]187607565-187607867 | 1.856708667 | 5.776175 | -1.63736699 | 0.002699456 | 0.009778795 |
| R6Z07_016995 | CLIC5 | CP162259.1[-]25770212-25931563 | 1.843463 | 0.917675 | 1.00636325 | 0.005601907 | 0.018335504 |
| R6Z07_006771 | CAPS | CP162244.1[-]21315000-21315820 | 1.842673333 | 0.568444 | 1.6967102 | 0.008466662 | 0.025974084 |
| R6Z07_005612 | IL17REL | CP162242.1[-]230337312-230358815 | 1.835437333 | 0.774314667 | 1.245131984 | 0.002929638 | 0.010504028 |
| R6Z07_009168 | CNR1 | CP162247.1[+]54387798-54413565 | 1.830102 | 3.932322 | -1.103457403 | 2.85E-05 | 0.000167463 |
| R6Z07_004517 | NEURL3 | CP162242.1[+]104186949-104211057 | 1.797988333 | 0.237184 | 2.922305063 | 6.13E-06 | 4.07E-05 |
| R6Z07_014045 | LOC101115059 | CP162253.1[+]68898289-68909588 | 1.796944667 | 3.827597 | -1.090892955 | 0.014763639 | 0.041335994 |
| R6Z07_000183 | RIMS3 | CP162240.1[-]15667076-15683059 | 1.771229333 | 5.146378 | -1.538806407 | 4.08E-05 | 0.000233659 |
| R6Z07_017658 | SYT7 | CP162260.1[-]41524617-41584075 | 1.758434667 | 0.827651 | 1.087197282 | 0.003624152 | 0.012595494 |
| R6Z07_006414 | CILP2 | CP162244.1[-]8339519-8347956 | 1.753156333 | 0.532157667 | 1.720028997 | 7.21E-10 | 8.56E-09 |
| R6Z07_007935 | LOC101112981 | CP162245.1[-]113453288-113487158 | 1.726902 | 0.324989667 | 2.409720461 | 4.53E-06 | 3.07E-05 |
| R6Z07_014036 | LOC121816000 | CP162253.1[+]68592559-68622316 | 1.706868 | 4.754006 | -1.477792232 | 4.85E-05 | 0.000273298 |
| R6Z07_010831 | MFAP4 | CP162250.1[+]35036935-35038749 | 1.697637667 | 3.578118333 | -1.075672528 | 0.008214368 | 0.025292801 |
| R6Z07_008586 | MYZAP | CP162246.1[-]59052482-59177036 | 1.693726667 | 0.827259 | 1.033790084 | 0.005820656 | 0.018944481 |
| R6Z07_008666 | DUOXA1 | CP162246.1[+]71782457-71791654 | 1.683018 | 0.689347333 | 1.287747621 | 0.001697078 | 0.006502588 |
| R6Z07_014790 | FGF18 | CP162255.1[+]5473676-5508374 | 1.679076333 | 3.722978333 | -1.148789401 | 0.013133982 | 0.037533493 |
| R6Z07_014296 | GRAMD1B | CP162254.1[+]54625357-54814641 | 1.671479667 | 0.492589333 | 1.762668512 | 3.40E-09 | 3.68E-08 |
| R6Z07_004368 | RMDN2 | CP162242.1[-]86378376-86439198 | 1.661056667 | 3.688552667 | -1.150953544 | 0.001011233 | 0.004137762 |
| R6Z07_012277 | FAM171A1 | CP162252.1[-]36435232-36563482 | 1.607728333 | 3.543031 | -1.139960441 | 2.18E-06 | 1.55E-05 |
| R6Z07_000458 | AK5 | CP162240.1[+]54444079-54715036 | 1.596544 | 0.706785667 | 1.175607625 | 0.000693839 | 0.002970554 |
| R6Z07_017601 | ST14 | CP162260.1[+]37129767-37167685 | 1.586350667 | 0.634780667 | 1.321381623 | 4.85E-06 | 3.27E-05 |
| R6Z07_014936 | ISL1 | CP162255.1[-]29995229-30004830 | 1.584064 | 0.712912667 | 1.151833366 | 0.012175518 | 0.035201757 |
| R6Z07_008461 | RHOV | CP162246.1[-]42906608-42908280 | 1.551726333 | 0.436538333 | 1.829693892 | 0.004978158 | 0.016545538 |
| R6Z07_005722 | DGKB | CP162243.1[-]31707755-32517181 | 1.529481333 | 0.724973 | 1.077043328 | 0.000142717 | 0.000720084 |
| R6Z07_019454 | MAP10 | CP162264.1[+]14568995-14571724 | 1.526053333 | 3.591227333 | -1.234671599 | 8.76E-07 | 6.62E-06 |
| R6Z07_007233 | KCTD16 | CP162244.1[+]58080098-58389551 | 1.525072 | 0.627117333 | 1.282070054 | 0.002526721 | 0.009225816 |
| R6Z07_016902 | MDGA1 | CP162259.1[-]18205336-18264716 | 1.516386667 | 0.708418 | 1.097964902 | 0.000249749 | 0.001194892 |
| R6Z07_017189 | ZSCAN12 | CP162259.1[+]36286461-36298241 | 1.495833667 | 3.061688 | -1.033377513 | 0.000384204 | 0.001758861 |
| R6Z07_002233 | LOC101112888 | CP162241.1[+]29433705-29507663 | 1.491380333 | 3.170739 | -1.088170905 | 4.38E-05 | 0.000248513 |
| R6Z07_001941 | LRRC3 | CP162240.1[+]274275088-274285002 | 1.479117667 | 0.640152 | 1.208250417 | 0.006127879 | 0.019756607 |
| R6Z07_015078 | ADAMTS16 | CP162255.1[-]70330615-70516193 | 1.464594333 | 4.197832333 | -1.519143426 | 9.42E-18 | 2.50E-16 |
| R6Z07_008900 | ADCK1 | CP162246.1[+]95413003-95558243 | 1.460998 | 3.097684 | -1.084235777 | 0.004968942 | 0.016527193 |
| R6Z07_007147 | GFRA3 | CP162244.1[-]52425409-52442904 | 1.447007667 | 0.639931 | 1.177084304 | 0.010978761 | 0.032312172 |
| R6Z07_002715 | FAM167A | CP162241.1[-]105942797-105956666 | 1.429613667 | 0.480156667 | 1.574048218 | 0.016189575 | 0.044639849 |
| R6Z07_007430 | ST8SIA4 | CP162244.1[-]102778994-102881426 | 1.412566 | 0.531568 | 1.409992114 | 0.004693254 | 0.015744504 |
| R6Z07_002650 | TP-1 | CP162241.1[+]91060330-91070864 | 1.412455667 | 2.889033333 | -1.032381264 | 0.015922557 | 0.044006875 |
| R6Z07_006996 | TRIM52 | CP162244.1[-]43202259-43209603 | 1.412360333 | 4.257386667 | -1.591859917 | 2.71E-05 | 0.000159929 |
| R6Z07_014433 | HBB | CP162254.1[+]68915148-68961429 | 1.410174667 | 0.169687 | 3.054925921 | 0.004604986 | 0.015500159 |
| R6Z07_002340 | PHYHIP | CP162241.1[+]43826045-43850407 | 1.405485667 | 0.701520333 | 1.002511915 | 0.016224376 | 0.044719439 |
| R6Z07_000192 | EDN2 | CP162240.1[-]16519762-16525001 | 1.393875333 | 0.106676333 | 3.707789486 | 0.00093522 | 0.003860282 |
| R6Z07_011205 | PECAM1 | CP162250.1[-]49484732-49549780 | 1.373579333 | 0.652560333 | 1.073757039 | 0.000670647 | 0.002883554 |
| R6Z07_003736 | ABO | CP162242.1[+]2719682-2775347 | 1.356021333 | 0.359975333 | 1.913409919 | 0.000151478 | 0.000760217 |
| R6Z07_015278 | ZNF84 | CP162256.1[+]51250534-51271098 | 1.336735667 | 2.93274 | -1.133534968 | 2.18E-05 | 0.000130429 |
| R6Z07_009437 | THBS2 | CP162247.1[-]97418664-97476463 | 1.317337667 | 3.986608667 | -1.597536803 | 6.38E-17 | 1.57E-15 |
| R6Z07_007798 | LOC101120331 | CP162245.1[-]95844148-95862932 | 1.293963333 | 0.463227333 | 1.482004448 | 0.014612481 | 0.041019496 |
| R6Z07_016892 | PI16 | CP162259.1[+]17559616-17572255 | 1.25674 | 0.444471333 | 1.49952393 | 0.001689069 | 0.006475194 |
| R6Z07_011310 | RBFOX3 | CP162250.1[+]53621150-53999908 | 1.255435667 | 0.575492667 | 1.125318652 | 0.013450167 | 0.038270106 |
| R6Z07_004074 | CLIP4 | CP162242.1[+]36085670-36142812 | 1.254183667 | 0.510779333 | 1.295976578 | 0.001185324 | 0.004760886 |
| R6Z07_008783 | TMEM229B | CP162246.1[-]85779359-85779862 | 1.248338333 | 3.369989333 | -1.432735029 | 0.011650938 | 0.033954893 |
| R6Z07_021225 | CPNE6 | CP162246.1[-]28357413-28362093 | 1.234232667 | 0.312897333 | 1.979853116 | 3.03E-05 | 0.000177114 |
| R6Z07_007472 | NPY1R | CP162245.1[+]11784418-11785676 | 1.206982667 | 4.426091667 | -1.874628373 | 4.39E-08 | 4.06E-07 |
| R6Z07_001960 | LSS | CP162240.1[-]275770298-275800254 | 1.201391333 | 2.485315667 | -1.048722942 | 0.000346303 | 0.001600431 |
| R6Z07_000272 | TSPAN1 | CP162240.1[+]21283786-21288889 | 1.180541333 | 3.187916667 | -1.433165363 | 0.001626079 | 0.006260798 |
| R6Z07_010885 | EPN3 | CP162250.1[-]37112393-37118111 | 1.172223333 | 2.425146667 | -1.048824541 | 0.002105866 | 0.007841566 |
| R6Z07_007699 | ATP8A1 | CP162245.1[-]71581957-71806218 | 1.152418333 | 0.399231667 | 1.529366454 | 8.10E-07 | 6.17E-06 |
| R6Z07_012135 | TNNT1 | CP162251.1[+]94448614-94459891 | 1.150206333 | 0.466490667 | 1.301972565 | 0.015364761 | 0.042709499 |
| R6Z07_001001 | TSACC | CP162240.1[+]108721901-108727854 | 1.148391667 | 4.489965667 | -1.967089646 | 1.93E-05 | 0.000117367 |
| R6Z07_010498 | ADAP2 | CP162250.1[-]18888541-18916923 | 1.141032333 | 0.261348333 | 2.12629381 | 0.00114649 | 0.004625877 |
| R6Z07_002951 | SLC38A11 | CP162241.1[+]148679656-148743619 | 1.140346667 | 0.536573333 | 1.08762521 | 0.017488576 | 0.047637477 |
| R6Z07_002212 | BARX1 | CP162241.1[+]27705806-27708811 | 1.124589 | 0.153918667 | 2.869157729 | 0.000857127 | 0.003572975 |
| R6Z07_005327 | LOC105612575 | CP162242.1[+]210583854-210624778 | 1.118365 | 0.393646 | 1.506420393 | 1.91E-05 | 0.000116266 |
| R6Z07_010127 | MTUS2 | CP162249.1[-]40737753-40997682 | 1.109367 | 0.461519 | 1.265274768 | 5.40E-06 | 3.61E-05 |
| R6Z07_010092 | DCLK1 | CP162249.1[+]35062623-35409976 | 1.107985333 | 2.278537667 | -1.040169436 | 0.002073725 | 0.007735265 |
| R6Z07_002279 | DNAI1 | CP162241.1[-]37033085-37101656 | 1.104309 | 0.497658 | 1.149917373 | 0.001791153 | 0.006805931 |
| R6Z07_010078 | POSTN | CP162249.1[+]33967336-34000851 | 1.083871 | 4.959284333 | -2.193938882 | 1.55E-19 | 4.69E-18 |
| R6Z07_013625 | KLC3 | CP162253.1[+]55485845-55494520 | 1.027161667 | 0.319710667 | 1.683824484 | 0.001084128 | 0.004402227 |
| R6Z07_019884 | DUSP26 | CP162265.1[-]51012038-51017980 | 1.025692333 | 0.210258667 | 2.286360872 | 0.004910561 | 0.0163696 |
| R6Z07_004467 | TMEM182 | CP162242.1[-]99243749-99310904 | 1.024142667 | 3.131445667 | -1.612412145 | 0.000863411 | 0.003594404 |
| R6Z07_019567 | PALD1 | CP162264.1[+]35476448-35517526 | 1.021812333 | 2.712411 | -1.408445546 | 2.15E-07 | 1.81E-06 |
| R6Z07_021336 | LRRC70 | CP162255.1[-]19106676-19108571 | 1.014617667 | 0.500838333 | 1.018519293 | 0.017636881 | 0.047997778 |
| R6Z07_001359 | DZIP3 | CP162240.1[+]183256725-183363008 | 1.014477667 | 3.289791333 | -1.697258974 | 2.02E-14 | 3.85E-13 |
| R6Z07_015840 | FSD2 | CP162257.1[-]23620237-23641366 | 1.012357667 | 2.817351333 | -1.476620399 | 9.17E-07 | 6.92E-06 |
| R6Z07_018550 | EMILIN2 | CP162262.1[+]54219370-54276714 | 1.009654333 | 2.337606667 | -1.211170743 | 4.25E-06 | 2.90E-05 |
| R6Z07_011453 | SLC16A6 | CP162250.1[+]62363876-62382566 | 1.002295667 | 3.403920667 | -1.763889262 | 1.18E-09 | 1.36E-08 |
| R6Z07_002983 | GALNT5 | CP162241.1[-]156527308-156566317 | 1.000078667 | 0.476284667 | 1.070217479 | 0.001631542 | 0.006280228 |
| R6Z07_002320 | NKX3-1 | CP162241.1[+]42146221-42192404 | 0.996043 | 0.258224333 | 1.947583069 | 0.014562842 | 0.04093354 |
| R6Z07_013180 | CDH16 | CP162253.1[-]36467162-36477573 | 0.976329333 | 2.816876667 | -1.528656617 | 7.59E-08 | 6.81E-07 |
| R6Z07_016096 | FBLN5 | CP162257.1[-]58994452-59081252 | 0.974341667 | 2.594426 | -1.412915719 | 0.00065067 | 0.002803259 |
| R6Z07_011886 | LOC101107037 | CP162251.1[-]64485392-64496966 | 0.955043667 | 0.332186333 | 1.523573978 | 0.001595359 | 0.006159837 |
| R6Z07_009193 | RIPPLY2 | CP162247.1[-]58180928-58185090 | 0.908380333 | 0.058785333 | 3.949768312 | 0.017222444 | 0.04705719 |
| R6Z07_008565 | LOC101102490 | CP162246.1[+]54408493-54410090 | 0.90561 | 2.515236667 | -1.47373236 | 7.76E-05 | 0.000415253 |
| R6Z07_005581 | R6Z07_005581 | CP162242.1[+]227319746-227330788 | 0.890202667 | 0.396786 | 1.165772699 | 0.00547882 | 0.017987341 |
| R6Z07_001735 | ARHGEF26 | CP162240.1[-]243172823-243313690 | 0.878747667 | 0.319442667 | 1.459891933 | 0.000337735 | 0.001568047 |
| R6Z07_020786 | CAPN6 | CP162266.1[+]126250272-126267052 | 0.877238667 | 0.292144333 | 1.586288098 | 0.00180984 | 0.006866543 |
| R6Z07_016169 | SLC25A47 | CP162257.1[+]67487445-67492595 | 0.864674667 | 0.263893333 | 1.712202517 | 0.013308396 | 0.037952735 |
| R6Z07_019148 | ITGAD | CP162263.1[+]33825328-33851802 | 0.854393667 | 0.267982 | 1.672764853 | 6.39E-06 | 4.22E-05 |
| R6Z07_011885 | RNF223 | CP162251.1[+]64475820-64483335 | 0.851524 | 0.237762667 | 1.840524991 | 0.012517737 | 0.036080396 |
| R6Z07_016370 | ZNF35 | CP162258.1[+]18488466-18526704 | 0.851102667 | 2.143323 | -1.332444205 | 0.00497445 | 0.016536863 |
| R6Z07_003245 | CXCR2 | CP162241.1[+]224400650-224415960 | 0.842154667 | 2.854238333 | -1.760948685 | 1.63E-05 | 0.000100434 |
| R6Z07_011104 | PYY | CP162250.1[-]44780369-44793619 | 0.841129667 | 0.0001 | 13.0381125 | 0.001198009 | 0.004806712 |
| R6Z07_012844 | MMP9 | CP162252.1[+]82424936-82432001 | 0.837903667 | 0.270106 | 1.6332587 | 0.001909392 | 0.007207945 |
| R6Z07_002118 | SLC46A2 | CP162241.1[+]10831619-10842625 | 0.815061667 | 0.256733 | 1.666640467 | 0.005404707 | 0.017778889 |
| R6Z07_003067 | SCTR | CP162241.1[-]188900759-188973355 | 0.810911333 | 1.763831667 | -1.121096801 | 0.014411071 | 0.040552334 |
| R6Z07_016790 | BEND6 | CP162259.1[-]9930288-9971149 | 0.809037333 | 2.585357 | -1.676085326 | 0.001699721 | 0.006511061 |
| R6Z07_013904 | LOC114117927 | CP162253.1[-]63924098-63960243 | 0.8051 | 2.204929667 | -1.453492743 | 0.012814472 | 0.036745877 |
| R6Z07_007027 | SHC2 | CP162244.1[-]45867867-45893642 | 0.798066 | 1.795397667 | -1.169723458 | 0.003964058 | 0.013619725 |
| R6Z07_006197 | LOC101109961 | CP162243.1[+]114915396-114945889 | 0.792442 | 0.191717667 | 2.04732206 | 0.015592545 | 0.043238962 |
| R6Z07_004232 | ECRG4 | CP162242.1[+]61114673-61124089 | 0.787442 | 0.042027667 | 4.227762397 | 0.002074845 | 0.007737527 |
| R6Z07_016347 | R6Z07_016347 | CP162258.1[-]16845719-16847975 | 0.785887667 | 0.0001 | 12.9401074 | 0.000920108 | 0.003808339 |
| R6Z07_015250 | R6Z07_015250 | CP162256.1[+]42818696-42827595 | 0.781247667 | 0.0001 | 12.93156426 | 0.001881307 | 0.007112617 |
| R6Z07_010746 | ALOXE3 | CP162250.1[-]28749085-28774849 | 0.777864 | 0.384973667 | 1.014758175 | 0.009984711 | 0.02979395 |
| R6Z07_017210 | LOC101105537 | CP162259.1[-]37255423-37298337 | 0.775675 | 0.225258333 | 1.783871825 | 0.001907486 | 0.007202552 |
| R6Z07_013066 | CENPBD1 | CP162253.1[-]16651040-16652671 | 0.775066333 | 3.305418 | -2.092441033 | 1.82E-10 | 2.36E-09 |
| R6Z07_013488 | LOC101118470 | CP162253.1[+]51282625-51299136 | 0.773315 | 1.566437667 | -1.018359259 | 0.004042546 | 0.013857793 |
| R6Z07_010348 | TMEM255B | CP162249.1[+]99166731-99188714 | 0.773134333 | 0.123898 | 2.641566207 | 0.003311381 | 0.011650928 |
| R6Z07_012901 | ATP9A | CP162252.1[-]87150536-87274576 | 0.772745333 | 1.592004 | -1.042779019 | 0.000616216 | 0.00267163 |
| R6Z07_019036 | ERN2 | CP162263.1[-]27759729-27783698 | 0.769270333 | 0.205500667 | 1.904347598 | 5.08E-05 | 0.000284817 |
| R6Z07_009287 | R6Z07_009287 | CP162247.1[+]74260850-74273001 | 0.768231333 | 0.150380333 | 2.352924903 | 0.014953257 | 0.041773738 |
| R6Z07_013384 | KIRREL2 | CP162253.1[+]48163481-48172426 | 0.768012333 | 0.326490667 | 1.234087729 | 0.002942258 | 0.010541055 |
| R6Z07_011183 | CYB561 | CP162250.1[-]48716252-48731292 | 0.766682 | 0.234835 | 1.706980864 | 0.018333569 | 0.049589506 |
| R6Z07_018639 | LIPG | CP162262.1[+]66144587-66169455 | 0.757324 | 1.668594 | -1.139650409 | 0.005826791 | 0.018960353 |
| R6Z07_000987 | RXFP4 | CP162240.1[+]108382457-108386144 | 0.747988667 | 3.002284 | -2.004972139 | 4.07E-06 | 2.78E-05 |
| R6Z07_008862 | MLH3 | CP162246.1[-]92717376-92742949 | 0.744610333 | 1.787965 | -1.263760953 | 6.77E-07 | 5.25E-06 |
| R6Z07_002832 | FRZB | CP162241.1[+]130962818-130994301 | 0.742077333 | 1.887988 | -1.347208149 | 0.01426489 | 0.040223626 |
| R6Z07_006802 | PLIN4 | CP162244.1[+]22431324-22441451 | 0.733907667 | 2.120238667 | -1.530556199 | 1.58E-09 | 1.80E-08 |
| R6Z07_000337 | PODN | CP162240.1[+]28088751-28142414 | 0.710038333 | 1.440437333 | -1.020538077 | 0.011470954 | 0.033512001 |
| R6Z07_003569 | SH2D5 | CP162241.1[+]249992238-249997660 | 0.690250333 | 2.147243667 | -1.637294331 | 0.000110412 | 0.000574429 |
| R6Z07_006232 | LOC105612738 | CP162243.1[-]116397056-116398323 | 0.676011333 | 2.428248333 | -1.844796633 | 0.018270671 | 0.049437128 |
| R6Z07_002566 | TYRP1 | CP162241.1[+]81742830-81758780 | 0.656657 | 0.170679 | 1.943854423 | 0.00593574 | 0.0192525 |
| R6Z07_003927 | RSAD2 | CP162242.1[+]16766083-16782428 | 0.652038333 | 6.253025333 | -3.261525674 | 2.38E-19 | 7.13E-18 |
| R6Z07_017430 | LOC101122242 | CP162260.1[-]6091515-6092087 | 0.647407667 | 2.62026 | -2.016963618 | 0.002107034 | 0.007843977 |
| R6Z07_011584 | ATP2B4 | CP162251.1[-]20427751-20452560 | 0.642090333 | 0.089014667 | 2.85066131 | 0.002400349 | 0.008820403 |
| R6Z07_020908 | PABPC5 | CP162266.1[-]145233633-145234781 | 0.636650333 | 1.892871 | -1.572002969 | 0.001209811 | 0.004843746 |
| R6Z07_004218 | IL1B | CP162242.1[+]60324265-60336253 | 0.636572667 | 2.151968 | -1.757259508 | 0.000739689 | 0.003138316 |
| R6Z07_013303 | LRP3 | CP162253.1[+]45431031-45442796 | 0.634125 | 1.337932667 | -1.077166352 | 0.006229499 | 0.020054195 |
| R6Z07_001100 | NECTIN4 | CP162240.1[-]115015948-115030458 | 0.607954333 | 1.584906 | -1.382362413 | 0.003341509 | 0.011735007 |
| R6Z07_008130 | HOMER1 | CP162246.1[-]17862485-17991747 | 0.605905667 | 2.287752667 | -1.916765985 | 0.000125029 | 0.000641363 |
| R6Z07_013712 | SULT2B1 | CP162253.1[+]57696873-57732947 | 0.590082667 | 0.038735333 | 3.929195021 | 0.00172973 | 0.00661164 |
| R6Z07_005918 | GHRHR | CP162243.1[-]75380398-75395574 | 0.589994 | 0.160558333 | 1.877602737 | 0.01047843 | 0.031082503 |
| R6Z07_015902 | TARS3 | CP162257.1[+]31184248-31228726 | 0.588255667 | 0.184369667 | 1.673841996 | 0.003422587 | 0.011977864 |
| R6Z07_011284 | R6Z07_011284 | CP162250.1[+]52224884-52229548 | 0.58464 | 0.044511333 | 3.715303917 | 0.014653186 | 0.041095476 |
| R6Z07_014248 | LYRM9 | CP162254.1[-]50009059-50033114 | 0.584003 | 0.0001 | 12.51176006 | 0.00201375 | 0.007539552 |
| R6Z07_000724 | SLC22A15 | CP162240.1[+]94038144-94160424 | 0.572922 | 1.287881 | -1.168588652 | 0.001442131 | 0.005646349 |
| R6Z07_008699 | CCDC198 | CP162246.1[-]76235844-76264766 | 0.566882333 | 2.358227333 | -2.056581587 | 0.000156424 | 0.000782434 |
| R6Z07_010334 | MCF2L | CP162249.1[+]98800649-98887005 | 0.564637333 | 0.248597333 | 1.183513701 | 0.002863848 | 0.01030739 |
| R6Z07_007205 | LOC101123376 | CP162244.1[+]55036288-55043765 | 0.561402667 | 1.144494 | -1.027602078 | 0.002577442 | 0.009390988 |
| R6Z07_012252 | ENKUR | CP162252.1[-]32458019-32489762 | 0.556166667 | 2.344593667 | -2.07574873 | 0.00028439 | 0.001342299 |
| R6Z07_020370 | P2RY4 | CP162266.1[-]64467717-64469299 | 0.553677 | 0.107527333 | 2.364341155 | 0.013377675 | 0.038121439 |
| R6Z07_009925 | SLC26A7 | CP162248.1[-]91486319-91632535 | 0.545190333 | 1.238232333 | -1.18345015 | 0.006467751 | 0.020701749 |
| R6Z07_011833 | ARHGEF16 | CP162251.1[-]62687303-62712541 | 0.524224667 | 1.214000667 | -1.211512069 | 0.009957224 | 0.029741384 |
| R6Z07_004530 | PROM2 | CP162242.1[-]104676026-104690610 | 0.522534667 | 0.20554 | 1.34610757 | 0.017670164 | 0.04806259 |
| R6Z07_015168 | REELD1 | CP162256.1[-]19248256-19264936 | 0.522478667 | 0.148101 | 1.81879075 | 0.017366435 | 0.047373295 |
| R6Z07_005259 | BCL2L14 | CP162242.1[-]206595187-206634318 | 0.507628 | 0.074895 | 2.760830336 | 0.009144759 | 0.027771606 |
| R6Z07_009605 | TMEM71 | CP162248.1[+]27124699-27149581 | 0.493620333 | 0.043770667 | 3.495365561 | 0.006852416 | 0.021761911 |
| R6Z07_001742 | SUCNR1 | CP162240.1[-]245770956-245778224 | 0.49245 | 0.064361667 | 2.93570366 | 0.016173048 | 0.044618777 |
| R6Z07_017305 | STMND1 | CP162259.1[-]46275066-46297203 | 0.481385 | 0.038426333 | 3.647023963 | 0.013808445 | 0.039112244 |
| R6Z07_007471 | NPY5R | CP162245.1[-]11753083-11754423 | 0.470509667 | 1.815291333 | -1.947904834 | 0.000421184 | 0.001905916 |
| R6Z07_010916 | IGF2BP1 | CP162250.1[-]38450868-38490417 | 0.455238 | 0.067688 | 2.749648994 | 0.004617842 | 0.015533024 |
| R6Z07_012119 | LGR6 | CP162251.1[-]93836263-93918252 | 0.450040667 | 0.078151667 | 2.525706827 | 0.000299352 | 0.001405433 |
| R6Z07_008645 | SHC4 | CP162246.1[+]67738047-67869110 | 0.446761333 | 1.535318667 | -1.780961896 | 3.68E-05 | 0.000212387 |
| R6Z07_006068 | IQUB | CP162243.1[-]97810402-97882967 | 0.446308333 | 1.008071667 | -1.175485558 | 0.012575506 | 0.036198429 |
| R6Z07_008346 | LOC101103880 | CP162246.1[-]31974501-32024234 | 0.444383 | 0.0001 | 12.11758791 | 0.003042786 | 0.010829817 |
| R6Z07_003337 | FBXO36 | CP162241.1[+]236295838-236394195 | 0.438218333 | 0.0001 | 12.09743413 | 0.004852132 | 0.016198143 |
| R6Z07_019087 | DOC2A | CP162263.1[+]32633514-32638543 | 0.436250667 | 0.066331667 | 2.717387655 | 0.003504362 | 0.012232789 |
| R6Z07_009220 | VNN1 | CP162247.1[-]63523581-63555371 | 0.429053667 | 0.043111667 | 3.315007871 | 0.001018278 | 0.004164326 |
| R6Z07_006201 | MGAM | CP162243.1[+]115052622-115148579 | 0.424909667 | 0.172538333 | 1.30023924 | 0.000611666 | 0.002654959 |
| R6Z07_011818 | CHD5 | CP162251.1[+]60261112-60325148 | 0.418203667 | 1.300816 | -1.637139292 | 1.30E-09 | 1.50E-08 |
| R6Z07_021433 | LOC132657144 | CP162264.1[+]36354155-36662042 | 0.418032333 | 0.124209 | 1.750844821 | 0.00020807 | 0.001014493 |
| R6Z07_006472 | UNC13A | CP162244.1[+]9965302-10029864 | 0.415953667 | 0.1906 | 1.125874715 | 0.00508488 | 0.016840802 |
| R6Z07_007559 | LOC101106685 | CP162245.1[+]31711340-31793966 | 0.415314667 | 1.060697 | -1.352735867 | 0.010668326 | 0.03155261 |
| R6Z07_021362 | RD3L | CP162257.1[-]70829291-70830256 | 0.414817333 | 0.0001 | 12.01826046 | 0.007440253 | 0.023309389 |
| R6Z07_015878 | LOC132659361 | CP162257.1[-]28557138-28557464 | 0.409322333 | 4.224767333 | -3.367562601 | 1.22E-05 | 7.68E-05 |
| R6Z07_000184 | R6Z07_000184 | CP162240.1[-]15683532-15707844 | 0.404690333 | 1.679591333 | -2.053219956 | 0.001467633 | 0.00572833 |
| R6Z07_012456 | SIGLEC1 | CP162252.1[+]58561459-58578094 | 0.402465 | 0.067888 | 2.567634837 | 8.11E-07 | 6.18E-06 |
| R6Z07_009447 | DLL1 | CP162247.1[-]98161417-98171071 | 0.402228667 | 0.86132 | -1.098533426 | 0.011143847 | 0.032737573 |
| R6Z07_007938 | CRMP1 | CP162245.1[+]113810347-113886637 | 0.384448 | 1.167662667 | -1.602763166 | 0.000328055 | 0.001527342 |
| R6Z07_004514 | FER1L5 | CP162242.1[-]104047002-104100665 | 0.381715667 | 0.122495333 | 1.639771612 | 5.97E-05 | 0.000328838 |
| R6Z07_007044 | AZU1 | CP162244.1[+]46149858-46152655 | 0.374103333 | 0.0001 | 11.86922111 | 0.004084062 | 0.013974673 |
| R6Z07_018908 | SEC14L5 | CP162263.1[+]10440387-10477179 | 0.369701667 | 0.046028667 | 3.00575699 | 0.001887666 | 0.007129501 |
| R6Z07_019027 | SCNN1G | CP162263.1[+]27276552-27304708 | 0.368710333 | 0.099814667 | 1.885164125 | 0.008845807 | 0.027010727 |
| R6Z07_020355 | PLA2G5 | CP162266.1[+]59705620-59706099 | 0.362640333 | 1.621617667 | -2.160822414 | 0.012380676 | 0.035726349 |
| R6Z07_012147 | PLCB4 | CP162252.1[+]9229287-9536706 | 0.361851 | 0.830855667 | -1.19920212 | 0.003102646 | 0.011006466 |
| R6Z07_009001 | RSPO3 | CP162247.1[-]16425422-16518231 | 0.359966667 | 1.532906667 | -2.090334636 | 0.006085837 | 0.019650498 |
| R6Z07_005329 | LOC106990118 | CP162242.1[+]210660783-210709023 | 0.355296667 | 0.106716 | 1.735247657 | 0.010320733 | 0.030663017 |
| R6Z07_005816 | SLC26A5 | CP162243.1[-]54524924-54561461 | 0.333648 | 0.078130333 | 2.094372181 | 0.007064415 | 0.022322246 |
| R6Z07_007093 | GDF9 | CP162244.1[-]47197175-47199661 | 0.331218333 | 0.956192 | -1.529517805 | 0.016578497 | 0.04554552 |
| R6Z07_017826 | CCDC87 | CP162260.1[-]46027571-46039065 | 0.327367667 | 1.138882333 | -1.798634956 | 6.03E-05 | 0.00033158 |
| R6Z07_014396 | R6Z07_014396 | CP162254.1[-]66807360-66808286 | 0.326338333 | 1.211300333 | -1.892116247 | 0.017085026 | 0.046758037 |
| R6Z07_008213 | HCN4 | CP162246.1[-]27452707-27494389 | 0.323620667 | 1.192412667 | -1.881507958 | 3.14E-07 | 2.56E-06 |
| R6Z07_006741 | LOC105609440 | CP162244.1[-]20031690-20033931 | 0.316975667 | 1.328482 | -2.067334682 | 0.005923642 | 0.019219435 |
| R6Z07_019924 | ANK1 | CP162265.1[-]58542591-58779849 | 0.312204 | 0.076030333 | 2.037842 | 0.000101557 | 0.00053238 |
| R6Z07_017847 | CARNS1 | CP162260.1[+]46690158-46710177 | 0.311591667 | 0.694218 | -1.155732123 | 0.004161373 | 0.014203726 |
| R6Z07_008240 | NRL | CP162246.1[+]28352415-28354941 | 0.304859667 | 1.974069333 | -2.694955463 | 0.001040201 | 0.004244763 |
| R6Z07_018552 | MYOM1 | CP162262.1[-]54400366-54540657 | 0.300937 | 0.095063333 | 1.662500601 | 0.000824685 | 0.003453226 |
| R6Z07_014991 | SLC1A3 | CP162255.1[-]40061536-40138346 | 0.298968667 | 1.340467667 | -2.164670224 | 2.80E-05 | 0.000164571 |
| R6Z07_009992 | FAM216B | CP162249.1[+]22802514-22810952 | 0.296789667 | 1.884686 | -2.666811414 | 0.00412792 | 0.014102323 |
| R6Z07_021052 | CLCA2 | CP162240.1[+]64241285-64283569 | 0.296332 | 0.051168 | 2.533900678 | 0.001211864 | 0.004849389 |
| R6Z07_015289 | P2RX2 | CP162256.1[-]51724247-51727388 | 0.291732667 | 0.012787333 | 4.511859599 | 0.006095058 | 0.019676054 |
| R6Z07_009237 | MYB | CP162247.1[+]65893840-65928045 | 0.285095333 | 1.239507333 | -2.120250478 | 6.61E-06 | 4.35E-05 |
| R6Z07_007135 | SPOCK1 | CP162244.1[-]51088771-51678864 | 0.282205333 | 0.938923 | -1.734261596 | 0.009265321 | 0.028069842 |
| R6Z07_001905 | TMPRSS2 | CP162240.1[-]271526327-271591756 | 0.275816667 | 0.031764667 | 3.118214852 | 0.01273979 | 0.036582958 |
| R6Z07_008183 | ITGA11 | CP162246.1[-]22583353-22717461 | 0.275302 | 1.346090333 | -2.289688234 | 5.40E-10 | 6.51E-09 |
| R6Z07_016078 | STRC | CP162257.1[-]57776663-57817098 | 0.271596333 | 0.115664667 | 1.231515787 | 0.00260142 | 0.009469193 |
| R6Z07_003500 | EXTL1 | CP162241.1[-]245143424-245159538 | 0.271104333 | 0.743179667 | -1.454862857 | 0.01184444 | 0.034436189 |
| R6Z07_007207 | LOC101102310 | CP162244.1[+]55063165-55078091 | 0.268468 | 0.702506667 | -1.38776178 | 0.000731346 | 0.003109924 |
| R6Z07_001082 | SLAMF1 | CP162240.1[-]114154382-114193021 | 0.268228667 | 0.0001 | 11.38924772 | 0.005070878 | 0.016801814 |
| R6Z07_010648 | WSCD1 | CP162250.1[-]26728610-26772524 | 0.253091 | 0.708212333 | -1.484525762 | 0.015490474 | 0.042995484 |
| R6Z07_019054 | IL21R | CP162263.1[+]31351953-31387528 | 0.249138667 | 0.01948 | 3.676883365 | 0.006454236 | 0.020671668 |
| R6Z07_001903 | MX2 | CP162240.1[+]271444251-271476118 | 0.247342 | 0.91719 | -1.890713388 | 0.0008261 | 0.00345819 |
| R6Z07_010610 | RAP1GAP2 | CP162250.1[+]24739985-24950620 | 0.245615667 | 0.704653667 | -1.520511769 | 0.00571229 | 0.018632032 |
| R6Z07_007565 | BANK1 | CP162245.1[-]32557285-32877337 | 0.244964 | 1.216248 | -2.311795782 | 8.73E-07 | 6.60E-06 |
| R6Z07_011276 | PDE6G | CP162250.1[+]51983219-51984390 | 0.242394333 | 2.064859 | -3.090615393 | 0.012286521 | 0.035481834 |
| R6Z07_019661 | LDB3 | CP162264.1[+]49888954-49959246 | 0.233769333 | 0.049988 | 2.225431973 | 0.003511168 | 0.012245199 |
| R6Z07_009566 | THEM6 | CP162248.1[-]20128925-20130938 | 0.231514333 | 1.154079333 | -2.31756898 | 0.013898067 | 0.039336518 |
| R6Z07_015361 | B3GNT4 | CP162256.1[-]60329131-60333611 | 0.226943667 | 0.0001 | 11.14811851 | 0.002997334 | 0.010698375 |
| R6Z07_000951 | KCNN3 | CP162240.1[-]107330720-107493410 | 0.224620333 | 0.031639 | 2.827712624 | 0.013009623 | 0.037227537 |
| R6Z07_018620 | ST8SIA5 | CP162262.1[-]63424120-63490587 | 0.220016 | 0.0001 | 11.10339273 | 0.004884501 | 0.016291762 |
| R6Z07_000619 | CELSR2 | CP162240.1[+]87303199-87327209 | 0.219389667 | 0.528263 | -1.26776079 | 0.000111874 | 0.000581407 |
| R6Z07_018641 | MYO5B | CP162262.1[-]66424846-66761957 | 0.217523333 | 1.030131 | -2.243585744 | 7.31E-10 | 8.66E-09 |
| R6Z07_011523 | CNTN2 | CP162251.1[+]16148137-16204584 | 0.206075667 | 0.027140667 | 2.924646098 | 0.00105678 | 0.004304405 |
| R6Z07_002940 | SCN7A | CP162241.1[+]147199857-147280371 | 0.200417 | 0.529933 | -1.402805082 | 0.003783723 | 0.013053715 |
| R6Z07_003950 | ATP6V1C2 | CP162242.1[+]19864677-19956113 | 0.183421 | 0.555716 | -1.599188955 | 0.008982615 | 0.027345337 |
| R6Z07_015654 | LOC101108295 | CP162256.1[+]79337082-79348042 | 0.182107333 | 0.732255 | -2.007557119 | 0.007751646 | 0.024154354 |
| R6Z07_021171 | ADM2 | CP162242.1[+]230762083-230763158 | 0.179154333 | 1.386068333 | -2.95172354 | 0.016441031 | 0.045209085 |
| R6Z07_018193 | CFAP43 | CP162261.1[-]26348882-26446674 | 0.174813667 | 0.040911 | 2.09525727 | 0.007601148 | 0.023734487 |
| R6Z07_011490 | LMNTD1 | CP162251.1[-]13972707-13973135 | 0.174321333 | 2.623333333 | -3.911580094 | 6.02E-05 | 0.000331355 |
| R6Z07_000203 | ZMYND12 | CP162240.1[-]17486471-17520908 | 0.169131667 | 0.813925667 | -2.266750242 | 0.009536502 | 0.028695213 |
| R6Z07_008621 | R6Z07_008621 | CP162246.1[+]64842753-64846458 | 0.165617333 | 0.701466333 | -2.082520192 | 0.012848803 | 0.03683731 |
| R6Z07_018755 | WFIKKN1 | CP162263.1[+]6927751-6930279 | 0.165101 | 1.282617667 | -2.95767042 | 6.95E-07 | 5.37E-06 |
| R6Z07_012999 | ADAD2 | CP162253.1[+]12393549-12399978 | 0.163196333 | 0.0001 | 10.67239293 | 0.002277998 | 0.008413904 |
| R6Z07_011237 | LOC101110855 | CP162250.1[+]51150813-51245548 | 0.159788667 | 0.0001 | 10.64194937 | 0.009256619 | 0.02804912 |
| R6Z07_007647 | SLC34A2 | CP162245.1[+]55318358-55343246 | 0.150666 | 3.333532667 | -4.467626071 | 2.96E-24 | 1.21E-22 |
| R6Z07_018792 | TMEM204 | CP162263.1[+]7575214-7586445 | 0.149616 | 1.207823 | -3.01307268 | 0.00153228 | 0.005954432 |
| R6Z07_010257 | LOC101109890 | CP162249.1[-]85074996-85282170 | 0.148997333 | 0.596359333 | -2.000895371 | 0.000117482 | 0.000606992 |
| R6Z07_020191 | SYTL5 | CP162266.1[+]38759590-38872585 | 0.140663333 | 0.0001 | 10.4580306 | 0.002861199 | 0.010300315 |
| R6Z07_014110 | MMP7 | CP162254.1[+]26665498-26673271 | 0.127121 | 1.537084667 | -3.595922351 | 3.43E-05 | 0.000198754 |
| R6Z07_011657 | MIXL1 | CP162251.1[+]42256445-42258591 | 0.126731667 | 0.810029333 | -2.676197095 | 0.015328671 | 0.042624908 |
| R6Z07_012226 | CCDC7 | CP162252.1[-]26613746-26863941 | 0.115766333 | 0.021873333 | 2.403970758 | 0.014994763 | 0.041874162 |
| R6Z07_003826 | PHYHD1 | CP162242.1[-]7491251-7506356 | 0.099386333 | 0.615089333 | -2.629676573 | 0.009778378 | 0.029288476 |
| R6Z07_019229 | ZP3 | CP162263.1[+]40744191-40751893 | 0.098961667 | 0.644613333 | -2.703492327 | 0.003956794 | 0.013598098 |
| R6Z07_014126 | LOC101121879 | CP162254.1[+]31848748-31849857 | 0.098026333 | 0.685688 | -2.806311009 | 0.006755809 | 0.021509514 |
| R6Z07_012593 | CASS4 | CP162252.1[-]66533950-66567586 | 0.095976 | 0.515305667 | -2.424682865 | 0.000812629 | 0.003410018 |
| R6Z07_016000 | COCH | CP162257.1[+]43926894-43940296 | 0.088017 | 0.536580333 | -2.607940074 | 0.001420072 | 0.005574468 |
| R6Z07_008870 | LOC101108756 | CP162246.1[+]93219945-93338178 | 0.08084 | 0.436475333 | -2.432758898 | 0.010358665 | 0.030751458 |
| R6Z07_010790 | MYOCD | CP162250.1[+]32090063-32172572 | 0.079903 | 0.0001 | 9.642105861 | 0.011414954 | 0.033374286 |
| R6Z07_011173 | EFCAB3 | CP162250.1[+]47709209-47815820 | 0.071741667 | 0.436777 | -2.606013721 | 0.000260304 | 0.001240271 |
| R6Z07_005936 | TRIL | CP162243.1[+]77197922-77200354 | 0.06314 | 0.363513667 | -2.525383439 | 0.003457519 | 0.012097303 |
| R6Z07_011532 | KLHDC8A | CP162251.1[-]16464615-16470069 | 0.060838 | 0.705781333 | -3.536176642 | 0.001737767 | 0.006636552 |
| R6Z07_013163 | CDH8 | CP162253.1[-]31239194-31618007 | 0.049956 | 0.299454333 | -2.58360614 | 0.005770037 | 0.018795973 |
| R6Z07_002422 | CCIN | CP162241.1[-]52839860-52841626 | 0.040672667 | 0.315489333 | -2.955459741 | 0.009933528 | 0.029694152 |
| R6Z07_004486 | AFF3 | CP162242.1[+]101478790-102056926 | 0.034210333 | 0.218119333 | -2.672613585 | 0.003126461 | 0.011080514 |
| R6Z07_008634 | USP50 | CP162246.1[+]66275554-66294701 | 0.031935333 | 0.427073 | -3.741257278 | 0.007855727 | 0.024387882 |
| R6Z07_010346 | ATP4B | CP162249.1[-]99134785-99140030 | 0.025903 | 0.566465333 | -4.450796568 | 0.001209024 | 0.00484188 |
| R6Z07_009205 | TMEM200A | CP162247.1[+]61278235-61279710 | 0.024091 | 0.395996333 | -4.038920887 | 0.003560227 | 0.01239907 |
| R6Z07_004041 | TCF24 | CP162242.1[+]34307722-34310880 | 0.022626667 | 0.663435667 | -4.873860605 | 0.003921304 | 0.013494374 |
| R6Z07_007505 | UGT8 | CP162245.1[-]20841564-20987604 | 0.019397667 | 0.290858667 | -3.906363265 | 0.006067513 | 0.019608669 |
| R6Z07_018707 | SERPINB13 | CP162262.1[+]78726372-78766187 | 0.015252 | 0.188997333 | -3.631295538 | 0.012537546 | 0.036109858 |
| R6Z07_011844 | PLCH2 | CP162251.1[-]63428957-63491718 | 0.007635667 | 0.105577 | -3.789397643 | 0.012363912 | 0.035691645 |
| R6Z07_009379 | FNDC1 | CP162247.1[+]89807497-89927033 | 0.005781667 | 0.065281 | -3.497105817 | 0.017293118 | 0.047198935 |
| R6Z07_014167 | ARHGAP20 | CP162254.1[-]41097008-41303989 | 0.0001 | 0.066071333 | -9.367880649 | 0.007552585 | 0.023606353 |
| R6Z07_001443 | CD86 | CP162240.1[+]196283469-196347177 | 0.0001 | 0.258442 | -11.33562483 | 0.007552585 | 0.023606353 |
| R6Z07_020769 | KLHL4 | CP162266.1[-]121297567-121433696 | 0.0001 | 0.100228333 | -9.969074683 | 0.014652445 | 0.041095476 |
| R6Z07_018381 | LOC101111846 | CP162261.1[-]53315737-53316783 | 0.0001 | 0.232929333 | -11.18567662 | 0.012145522 | 0.035135259 |
| R6Z07_020069 | LOC121818245 | CP162266.1[+]14639465-14640442 | 0.0001 | 0.694962667 | -12.76271976 | 0.000286321 | 0.001350339 |
| R6Z07_008602 | PIERCE2 | CP162246.1[-]61397344-61404389 | 0.0001 | 0.81642 | -12.99509581 | 0.003523262 | 0.012276009 |
| R6Z07_006674 | R6Z07_006674 | CP162244.1[-]18152923-18159872 | 0.0001 | 0.745284 | -12.86357457 | 0.001394774 | 0.005486598 |
| R6Z07_020274 | R6Z07_020274 | CP162266.1[-]54064588-54088147 | 0.0001 | 0.419637333 | -12.03492732 | 0.003321206 | 0.011680041 |
| R6Z07_013089 | ZNF423 | CP162253.1[-]19867124-20209357 | 0.0001 | 0.060301 | -9.236038117 | 0.017240749 | 0.047080872 |

#### List of differentially expressed metabolites in *VTN* overexpression

| **Index** | **Compounds** | **Class I** | **Formula** | **VIP** | **P-value** | **FDR** | **Log2FC** | **Type** |
| --- | --- | --- | --- | --- | --- | --- | --- | --- |
| MEDP0513 | Palmitic amide | FA | C16H33NO | 1.765836985 | 0.021143373 | 0.642832721 | -0.96159771 | down |
| MW0061712 | 14,15-Leukotriene C4(ExC4) | GP | C47H83O13P | 1.880224292 | 0.00578405 | 0.43581556 | 1.250339352 | up |
| MW0106972 | Glu-Met | Amino acid and Its metabolites | C10H18N2O5S | 1.899601563 | 0.000795586 | 0.212155892 | -2.160282906 | down |
| MEDN0212 | Threitol | Carbohydrates and Its metabolites | C4H10O4 | 1.983303532 | 0.006273556 | 0.453003021 | -2.597674378 | down |
| MEDN0343 | Shikimic Acid | Organic acid and Its derivatives | C7H10O5 | 1.911239723 | 0.014375574 | 0.592039578 | -1.443167387 | down |
| MW0058121 | 1-Palmitoyl-2-oleoyl-sn-glycero-3-phosphoethanolamine | GP | C39H76NO8P | 1.893247815 | 0.003193328 | 0.368935837 | 1.324061288 | up |
| MW0111244 | 1-Palmitoyl-2-thiopalmitoyl phosphatidylcholine | Organic acid and Its derivatives | C40H80NO7PS | 1.814453221 | 0.004841731 | 0.425972541 | 1.538741265 | up |
| MW0116986 | Cetylpyridinium | Heterocyclic compounds | C21H38N+ | 1.70808663 | 0.036909241 | 0.815977779 | 0.83851297 | up |
| MW0140636 | (2s)-2-(2,4-Dichlorophenoxy)propanoic acid | Benzene and substituted derivatives | C9H8Cl2O3 | 1.866784588 | 0.016883792 | 0.609575236 | -0.77134957 | down |
| MW0001300 | 1,2-Diphenylhydrazine | Benzene and substituted derivatives | C12H12N2 | 1.802367253 | 0.013141617 | 0.569360547 | 0.624775189 | up |
| MW0103464 | Adenosine-5'-phosphosulfate | Nucleotide and Its metabolites | C10H14N5O10PS | 1.928201734 | 0.007780249 | 0.481541845 | 1.279870948 | up |
| MW0106105 | Carbamic acid | Organic acid and Its derivatives | CH3NO2 | 1.903513497 | 0.012880346 | 0.569360547 | -1.686387394 | down |
| MW0007258 | Glycerol | Alcohol and amines | C3H8O3 | 1.911785277 | 0.007618764 | 0.481541845 | -1.167338709 | down |
| MW0194180 | 1,3-Benzenediol, 2-[3-methyl-6-(1-methylethenyl)-2-cyclohexen-1-yl]-5-pentyl-, (1R-trans)- | Alcohol and amines | C21H30O2 | 1.964536417 | 0.007714057 | 0.481541845 | -1.632801357 | down |
| MW0106487 | Djenkolic acid | Organic acid and Its derivatives | C7H14N2O4S2 | 1.809614352 | 0.010869472 | 0.538194151 | -1.229248945 | down |
| MW0012975 | 1-Hexadecanoyl-2-octadecanoyl-sn-glycero-3-phosphocholine | GP | C42H84NO8P | 1.767089222 | 0.048471822 | 0.815977779 | 1.186546481 | up |
| MEDP1448 | MG(18:0/0:0/0:0) | GL | C21H42O4 | 1.785931156 | 0.028127454 | 0.765475859 | -0.481251495 | down |
| MW0005071 | 4-Chloro-4'-hydroxybiphenyl | Benzene and substituted derivatives | C12H9ClO | 1.918260126 | 0.000856948 | 0.212155892 | -2.954978383 | down |
| MW0126509 | Granisetron | Heterocyclic compounds | C18H24N4O | 1.460378754 | 0.03615935 | 0.815977779 | 1.279235231 | up |
| MEDP1670 | Gly-Gly-Phe | Amino acid and Its metabolites | C13H17N3O4 | 1.816144709 | 0.035171187 | 0.815977779 | 1.446218979 | up |
| MW0055241 | N-acetylsphingosine | SP | C20H39NO3 | 1.742626446 | 0.030587489 | 0.791165942 | -0.355967433 | down |
| MW0015643 | Alloxanthin | Alcohol and amines | C40H52O2 | 1.754578502 | 0.036450046 | 0.815977779 | 0.544079364 | up |
| MW0107530 | Iminoaspartic acid | Amino acid and Its metabolites | C4H5NO4 | 1.94104432 | 0.008291457 | 0.492185798 | -1.82370521 | down |
| MW0139339 | Phenyl aminosalicylate | Aldehyde,Ketones,Esters | C13H11NO3 | 1.934990804 | 0.005267453 | 0.425972541 | -1.186049933 | down |
| MW0103376 | 3'-O-Methylguanosine | Nucleotide and Its metabolites | C11H15N5O5 | 1.904430671 | 0.004887213 | 0.425972541 | -1.747049463 | down |
| MW0154943 | Pavoninin-1 | Steroids | C37H59NO9 | 1.82281753 | 0.010190862 | 0.535174658 | -0.99232514 | down |
| MW0011610 | 4-Hexen-1-ol, 5-methyl-2-(1-methylethenyl)- | Alcohol and amines | C10H18O | 1.618646815 | 0.044790048 | 0.815977779 | 0.502841732 | up |
| MWS-04-329g_p | Zebularine | Nucleotide and Its metabolites | C9H12N2O5 | 1.633155339 | 0.039135514 | 0.815977779 | -0.617725935 | down |
| MW0011905 | 1,2-Dioleoyl-sn-glycero-3-phosphoethanolamine | GP | C41H78NO8P | 1.869796104 | 0.009624994 | 0.526369312 | 1.108052719 | up |
| MW0013614 | 1-Hexadecanoyl-2-(9Z-octadecenoyl)-sn-glycero-3-phosphoserine | GP | C40H76NO10P | 1.348415647 | 0.012233138 | 0.569360547 | -1.885241853 | down |
| MW0059581 | PE-NMe(18:4(6Z,9Z,12Z,15Z)/24:1(15Z)) | GP | C48H86NO8P | 1.741225371 | 0.016699911 | 0.609575236 | 0.619164525 | up |
| MW0059315 | PE-NMe(15:0/22:4(7Z,10Z,13Z,16Z)) | GP | C43H78NO8P | 1.895064142 | 8.52447E-05 | 0.073864496 | 3.006179342 | up |
| MW0161394 | 3-Hydroxy-alpha-ionone | Aldehyde,Ketones,Esters | C13H20O2 | 1.728472711 | 0.02178894 | 0.644995924 | 1.342376938 | up |
| MW0012940 | 1-Oleoyl-2-myristoyl-sn-glycero-3-phosphocholine | GP | C40H78NO8P | 1.97198104 | 0.000813622 | 0.212155892 | 1.81374573 | up |
| MW0057016 | 1-Oleoyl-2-palmitoyl-sn-glycero-3-phosphocholine | GP | C42H82NO8P | 1.78117191 | 0.041748882 | 0.815977779 | 1.244015297 | up |
| MEDP1895 | Tyr-Phe | Amino acid and Its metabolites | C18H20N2O4 | 1.718662132 | 0.013085024 | 0.569360547 | 1.535491551 | up |
| MW0146517 | (7R,8Z)-bacteriochlorophyll b | Heterocyclic compounds | C55H72MgN4O6 | 1.859500317 | 0.049425611 | 0.815977779 | 0.768711413 | up |
| MW0127405 | Beta-Mercaptoethanol | Others | C2H6OS | 1.955000482 | 0.015773555 | 0.607457141 | -3.365241037 | down |
| MW0059552 | PE-NMe(18:3(9Z,12Z,15Z)/24:1(15Z)) | GP | C48H88NO8P | 1.915319303 | 0.019770096 | 0.629670075 | 0.650483655 | up |
| MW0143427 | 4-O-(beta-L-Araf)-cis-L-Hyp | Amino acid and Its metabolites | C10H17NO7 | 1.882022007 | 0.018786772 | 0.625812558 | -0.898454899 | down |
| MW0012773 | 1-arachidonoyl-sn-glycero-3-phospho-1D-myo-inositol | GP | C29H49O12P | 1.765252804 | 0.045355314 | 0.815977779 | -0.975836709 | down |
| MW0104070 | [2-hydroxy-5-(2-hydroxy-3-methoxy-3-oxopropyl)phenyl]oxidanesulfonic acid | Organic acid and Its derivatives | C10H12O8S | 1.89420276 | 0.003673877 | 0.397926832 | -2.630672024 | down |
| MW0052974 | Galactosylceramide (d18:1/16:0) | SP | C40H77NO8 | 1.939060144 | 0.000263239 | 0.152064314 | 1.656174555 | up |
| MEDP1886 | Pro-val | Amino acid and Its metabolites | C10H18N2O3 | 1.891532105 | 0.000724872 | 0.212155892 | -1.903808097 | down |
| MW0057243 | PC(20:1(11Z)/P-18:1(11Z)) | GP | C46H88NO7P | 1.684555261 | 0.014689961 | 0.592039578 | 1.778333058 | up |
| MW0146045 | Asp-Asn-Thr | Amino acid and Its metabolites | C12H20N4O8 | 1.76686508 | 0.016170863 | 0.609219695 | -0.764057636 | down |
|  |  |  |  |  |  |  |  |  |
| MW0057175 | PC(18:4(6Z,9Z,12Z,15Z)/P-16:0) | GP | C42H76NO7P | 1.65097276 | 0.005407614 | 0.425972541 | 2.226862997 | up |
| MW0152516 | Leu-Thr-Phe-Gln-Met | Amino acid and Its metabolites | C29H46N6O8S | 1.861412551 | 0.003189965 | 0.368935837 | 1.480079148 | up |
| MW0155222 | Phe-Ile-Asp-Leu-Asn | Amino acid and Its metabolites | C29H44N6O9 | 1.98342437 | 0.002648889 | 0.368935837 | -1.082139924 | down |
| MW0146169 | Asp-Leu-Glu-Asp | Amino acid and Its metabolites | C19H30N4O11 | 1.702725408 | 0.015350115 | 0.6045852 | -1.520691535 | down |
| MW0012998 | LPC(18:0/0:0) | GP | C26H54NO7P | 1.74057657 | 0.031871994 | 0.800495164 | 0.930065787 | up |
| MEDP1055 | DL-Glyceraldehyde 3-Phosphate | Carbohydrates and Its metabolites | C3H7O6P | 1.69432811 | 0.036959877 | 0.815977779 | -0.919492799 | down |
| MW0169991 | Uridine | Nucleotide and Its metabolites | C9H12N2O6 | 1.913438826 | 0.014249037 | 0.592039578 | -0.955692351 | down |
| MW0103351 | 2'-Deoxyuridine | Nucleotide and Its metabolites | C9H12N2O5 | 1.982246389 | 5.45588E-05 | 0.073864496 | -0.540783771 | down |
| MW0114952 | N-Acetyl-D-glucosamine | Carbohydrates and Its metabolites | C8H15NO6 | 1.82932326 | 0.047333666 | 0.815977779 | 0.734348242 | up |
| MW0003876 | 5-Bromo-4-hydroxy-[1,1'-biphenyl]-3-carboxylic acid | Benzene and substituted derivatives | C13H9BrO3 | 1.780352509 | 0.0314388 | 0.800495164 | 0.394812179 | up |
| MW0126762 | Thiamine phosphoric acid ester | Organic acid and Its derivatives | C12H17N4O4PS | 1.966821394 | 0.002967626 | 0.368935837 | -0.902179791 | down |
| MW0114065 | Chlorogenic Acid | Organic acid and Its derivatives | C16H18O9 | 1.93698761 | 0.002277796 | 0.368935837 | -1.195814158 | down |
| MW0052535 | Enoxolone | Terpenoids | C30H46O4 | 1.775222714 | 0.007046967 | 0.481541845 | 0.969261923 | up |
| MW0005090 | 4-Hydroxybenzenesulfonic acid | Organic acid and Its derivatives | C6H6O4S | 1.689126167 | 0.04469414 | 0.815977779 | 0.764698715 | up |
| MW0169017 | Daidzein | Flavonoids | C15H10O4 | 1.658251252 | 0.033563835 | 0.815977779 | -0.41865112 | down |
| MW0103684 | Uridine-5'-diphosphate-glucose | Nucleotide and Its metabolites | C15H24N2O17P2 | 1.919882748 | 0.003004537 | 0.368935837 | -0.547405123 | down |
| MW0103710 | Uridine-diphosphate-N-acetylglucosamine | Nucleotide and Its metabolites | C17H27N3O17P2 | 1.549112827 | 0.048349918 | 0.815977779 | -0.70612485 | down |
| MEDP1238 | Coniferyl acetate | Aldehyde,Ketones,Esters | C12H14O4 | 1.832358015 | 0.019050549 | 0.625812558 | 0.483021717 | up |
| MW0146565 | Benz(l)aceanthrylene | Benzene and substituted derivatives | C20H12 | 1.701909954 | 0.021958892 | 0.644995924 | -0.729923545 | down |
| MEDN1485 | Glycerophospho-N-Arachidonoyl Ethanolamine | GP | C25H44NO7P | 1.844016645 | 0.038072715 | 0.815977779 | -2.319530852 | down |
| MW0013543 | 1-hexadecanoyl-2-(9Z,12Z-octadecadienoyl)-sn-glycero-3-phosphoethanolamine | GP | C39H74NO8P | 1.859585232 | 0.019983759 | 0.629670075 | -1.118373525 | down |
| MW0142555 | 2-phenyl-N-(5-propan-2-yl-2-thiazolyl)acetamide | Benzene and substituted derivatives | C14H16N2OS | 1.968720868 | 0.002088441 | 0.368935837 | -0.529569589 | down |
| MW0154827 | p-(3,4-Dihydro-6-methoxy-2-naphthyl)phenol | Benzene and substituted derivatives | C17H16O2 | 1.346466899 | 0.027187675 | 0.75993937 | -1.512261888 | down |
| MW0142653 | 3,3-Difluoro-17-methyl-5alpha-androstan-17beta-ol | Alcohol and amines | C20H32F2O | 1.792087242 | 0.010801144 | 0.538194151 | -0.617150332 | down |
| MW0154748 | Ophiobolin F | Terpenoids | C25H42O | 1.75796013 | 0.040143408 | 0.815977779 | 1.3253464 | up |
| MW0139261 | Pectolinarigenin | Flavonoids | C17H14O6 | 1.654013141 | 0.039071342 | 0.815977779 | -0.469946284 | down |
| MW0054414 | L-Palmitoylcarnitine | FA | C23H45NO4 | 1.830803868 | 0.03375068 | 0.815977779 | 0.770551357 | up |
| FDATN01468 | Trilithium citrate tetrahydrate | Others | C6H13Li3O11 | 1.800685797 | 0.02826916 | 0.765475859 | -0.936435007 | down |
| MW0105434 | Acrylic acid | Organic acid and Its derivatives | C3H4O2 | 1.881889435 | 0.005157951 | 0.425972541 | -0.434626508 | down |
| MW0053431 | Ganoderic acid Mi | Organic acid and Its derivatives | C33H52O6 | 1.747222533 | 0.041362654 | 0.815977779 | 0.575192156 | up |
| MW0063649 | 5-[7,13-Dihydroxy-13-[5-(1-hydroxytridecyl)oxolan-2-yl]tridecyl]-3-(2-oxopropyl)oxolan-2-one | FA | C37H68O7 | 1.744333302 | 0.026229887 | 0.745186778 | 0.494209256 | up |
| MW0000104 | 7-Ethyl-10-(4-N-aminopentanoic acid)-1-piperidino)carbonyloxycamptothecin | Alkaloids | C33H38N4O8 | 1.613659791 | 0.048702673 | 0.815977779 | -0.544830514 | down |
| MW0003077 | Vanillylmandelic acid | Benzene and substituted derivatives | C9H10O5 | 1.822083256 | 0.01803222 | 0.625812558 | -0.528725166 | down |
| MW0103500 | Clofarabine | Nucleotide and Its metabolites | C10H11ClFN5O3 | 1.759298541 | 0.041259528 | 0.815977779 | -0.959257476 | down |
| MW0148183 | Decahydro-2-naphthoic acid | Organic acid and Its derivatives | C11H18O2 | 1.869427703 | 0.012240871 | 0.569360547 | -0.613918497 | down |
| MW0103454 | Adpbetas | Nucleotide and Its metabolites | C10H15N5O9P2S | 1.81642514 | 0.008520239 | 0.492185798 | 0.570901083 | up |
| MW0157173 | (12-Methyl-4-methylidene-14,19-dioxa-17-azaheptacyclo[10.7.2.22,5.02,7.08,18.08,21.013,17]tricosan-3-yl) acetate | Terpenoids | C24H33NO4 | 1.780550461 | 0.0287544 | 0.766636533 | 1.045242278 | up |
| MW0014075 | 3-Hydroxy-6,8-dimethoxy-7(11)-eremophilen-12,8-olide | Heterocyclic compounds | C17H26O5 | 1.919575313 | 0.020691747 | 0.64033567 | 0.77292979 | up |
| MEDN0161 | Guanosine 3',5'-Cyclic Monophosphate | Nucleotide and Its metabolites | C10H11N5O7P- | 1.754631818 | 0.042324834 | 0.815977779 | 0.278702808 | up |
| MW0012846 | [2-hydroxy-3-[(9Z,12Z)-octadeca-9,12-dienoyl]oxypropyl] 2-(trimethylazaniumyl)ethyl phosphate | GP | C26H50NO7P | 1.863571196 | 0.009719456 | 0.526369312 | 0.410856158 | up |
| MW0194167 | Uridine-5'-diphospho-N-acetylgalactosamine disodium salt | Nucleotide and Its metabolites | C17H27N3O17P2 | 1.972338031 | 0.001435083 | 0.310874823 | -1.01666059 | down |
| MW0166716 | 3-hydroxy-4-methyl-L-kynurenine | Others | C11H14N2O4 | 1.809609594 | 0.045056526 | 0.815977779 | 1.589611532 | up |
| MW0143482 | 5-(3-Hydroxy-4-phenylbut-1-en-1-yl)-1-[6-(2H-tetrazol-5-yl)hexyl]pyrrolidin-2-one | Benzene and substituted derivatives | C21H29N5O2 | 1.669865595 | 0.037275029 | 0.815977779 | 0.438368058 | up |
| MW0141858 | 2-(2-Carboxy-4-methylthiazol-5-yl)ethyl phosphate | Organic acid and Its derivatives | C7H10NO6PS | 1.730269402 | 0.043646259 | 0.815977779 | 0.816318881 | up |
| MW0053732 | 2,3-Dihydroxypropyl 2-[(octadec-9-enoyl)amino]ethyl hydrogen phosphate | Aldehyde,Ketones,Esters | C23H46NO7P | 1.746248845 | 0.018628738 | 0.625812558 | -0.661299277 | down |
| MW0158578 | Tyr-Phe-Thr-Lys | Amino acid and Its metabolites | C28H39N5O7 | 1.87231526 | 0.005191699 | 0.425972541 | 1.065167267 | up |
| MW0061675 | Dipalmitoyl phosphatidylinositol | GP | C41H79O13P | 1.672998718 | 0.034156101 | 0.815977779 | 1.032873844 | up |
| MW0139445 | Psoromic acid | Organic acid and Its derivatives | C18H14O8 | 1.798540138 | 0.037772995 | 0.815977779 | 0.339559481 | up |
| MW0159465 | Zinc bacteriochlorophyll a | Heterocyclic compounds | C55H74N4O6Zn | 1.909778811 | 0.029309937 | 0.769607903 | 1.52935237 | up |
| MW0142256 | 2-Benzylmalate | Benzene and substituted derivatives | C11H12O5 | 1.840122334 | 0.043610685 | 0.815977779 | 0.818198496 | up |
| MW0062273 | 2-glyceryl-Prostaglandin H2 | Hormones and hormone related compounds | C23H38O7 | 1.733398965 | 0.025148375 | 0.726368896 | 0.433144749 | up |
| MW0057412 | Phosphatidylcholine(20:5/15:0) | GP | C43H76NO8P | 1.282952635 | 0.046446311 | 0.815977779 | -1.465527906 | down |
| MW0055663 | 1-octadecanoyl-2-(9Z-octadecenoyl)-sn-glycero-3-phosphate | GP | C39H75O8P | 1.61576084 | 0.039718271 | 0.815977779 | 0.723746566 | up |
| MW0156741 | Ser-Glu-Phe-Arg | Amino acid and Its metabolites | C23H35N7O8 | 1.862538398 | 0.043907063 | 0.815977779 | 0.416797393 | up |
| MW0145679 | Asn-Arg-Phe-Lys | Amino acid and Its metabolites | C25H41N9O6 | 1.839063579 | 0.042897166 | 0.815977779 | 0.643908214 | up |
| MW0145474 | Arg-Phe-Val-Asp | Amino acid and Its metabolites | C24H37N7O7 | 1.862320847 | 0.019139103 | 0.625812558 | 1.036504429 | up |
| MW0108678 | Nagaba | Amino acid and Its metabolites | C24H39NO3 | 1.268130928 | 0.031853788 | 0.815977779 | 0.969868553 | up |

#### Intersecting genes

#### ADM Group Genes Expression KEGG Enrichment of RECs

| **ID** | **Description** | **RichFactor** | **pvalue** | **qvalue** | **geneID** | **Count** | **Group** |
| --- | --- | --- | --- | --- | --- | --- | --- |
| bta05225 | Hepatocellular carcinoma | 0.0852272727272727 | 1.84271050796555e-07 | 3.37506977248428e-05 | CDKN1A/GADD45A/ACTG1/ACTB/GADD45B/GADD45G/GSTO1/MGST3/WNT7B/WNT10A/GSTA1/CCND1/WNT3A/MGST1/NQO1 | 15 | High vs Low group |
| bta05202 | Transcriptional misregulation in cancer | 0.0735294117647059 | 1.23511576538301e-06 | 9.63167380907161e-05 | CDKN1A/GADD45A/IGFBP3/GADD45B/DDIT3/DDX5/GADD45G/ID2/NFKBIZ/DUSP6/CCND2/BCL6/BIRC3/PPARG/HPGD | 15 | High vs Low group |
| bta04382 | Cornified envelope formation | 0.0721153846153846 | 1.57760174458932e-06 | 9.63167380907161e-05 | CLDN4/COL17A1/CLDN1/KRT4/S100A4/CLDN3/KRT14/ST14/KRT17/KRT8/S100A9/KRT19/KRT42/KRT6A/S100A8 | 15 | High vs Low group |
| bta04210 | Apoptosis | 0.0805369127516778 | 5.87337095719864e-06 | 0.000246940283754145 | FOS/GADD45A/JUN/ACTG1/ACTB/GADD45B/DDIT3/GADD45G/NFKBIA/MCL1/CTSC/BIRC3 | 12 | High vs Low group |
| bta05224 | Breast cancer | 0.0794701986754967 | 6.74118590708155e-06 | 0.000246940283754145 | CDKN1A/FOS/GADD45A/JUN/GADD45B/HES1/GADD45G/WNT7B/DLL1/WNT10A/CCND1/WNT3A | 12 | High vs Low group |
| bta04068 | FoxO signaling pathway | 0.0814814814814815 | 1.30009556141662e-05 | 0.000396871276642969 | CDKN1A/GADD45A/GADD45B/SGK1/PLK2/GADD45G/GABARAPL1/CCND2/BCL6/SIRT1/CCND1 | 11 | High vs Low group |
| bta04657 | IL-17 signaling pathway | 0.0882352941176471 | 4.33327998664287e-05 | 0.001133820628084 | FOSB/FOS/JUND/JUN/NFKBIA/TNFAIP3/S100A9/CXCL3/S100A8 | 9 | High vs Low group |
| bta05216 | Thyroid cancer | 0.142857142857143 | 5.7489589667578e-05 | 0.00131620902659981 | CDKN1A/GADD45A/GADD45B/GADD45G/CCND1/PPARG | 6 | High vs Low group |
| bta00982 | Drug metabolism - cytochrome P450 | 0.111111111111111 | 7.23615427244379e-05 | 0.00131758205479067 | AOX1/GSTO1/MGST3/MAOA/ADH5/GSTA1/MGST1 | 7 | High vs Low group |
| bta05217 | Basal cell carcinoma | 0.111111111111111 | 7.23615427244379e-05 | 0.00131758205479067 | CDKN1A/GADD45A/GADD45B/GADD45G/WNT7B/WNT10A/WNT3A | 7 | High vs Low group |
| bta04218 | Cellular senescence | 0.0670731707317073 | 7.91306463940373e-05 | 0.00131758205479067 | CDKN1A/GADD45A/IGFBP3/GADD45B/GADD45G/ZFP36L1/BOLA/CCND2/RASSF5/SIRT1/CCND1 | 11 | High vs Low group |
| bta00480 | Glutathione metabolism | 0.107692307692308 | 8.85826265907122e-05 | 0.00132450969787385 | ODC1/GSTO1/GCLM/MGST3/PRDX6/GSTA1/MGST1 | 7 | High vs Low group |
| bta04115 | p53 signaling pathway | 0.0909090909090909 | 9.40097400502419e-05 | 0.00132450969787385 | CDKN1A/GADD45A/IGFBP3/GADD45B/GADD45G/CCND2/SIAH1/CCND1 | 8 | High vs Low group |
| bta01200 | Carbon metabolism | 0.0769230769230769 | 0.000126654347518656 | 0.00165698168934182 | PSPH/ACADS/PSAT1/PHGDH/ACAT1/ADH5/SHMT2/ACAT2/PCCA | 9 | High vs Low group |
| bta05418 | Fluid shear stress and atherosclerosis | 0.0662251655629139 | 0.000186318061272823 | 0.00223033484184993 | DUSP1/FOS/JUN/ACTG1/ACTB/GSTO1/MGST3/GSTA1/MGST1/NQO1 | 10 | High vs Low group |
| bta00280 | Valine, leucine and isoleucine degradation | 0.115384615384615 | 0.000194833848253557 | 0.00223033484184993 | AOX1/HMGCS2/ACADS/ACAT1/ACAT2/PCCA | 6 | High vs Low group |
| bta04390 | Hippo signaling pathway | 0.0632911392405063 | 0.000269346143879274 | 0.00290193368637732 | ACTG1/ACTB/ID2/PARD6B/CCND2/WNT7B/WNT10A/BIRC3/CCND1/WNT3A | 10 | High vs Low group |
| bta00740 | Riboflavin metabolism | 0.333333333333333 | 0.000339083080380375 | 0.00327083090964801 | BLVRB/RFK/ACP5 | 3 | High vs Low group |
| bta04668 | TNF signaling pathway | 0.0671641791044776 | 0.000352744421862914 | 0.00327083090964801 | FOS/JUNB/JUN/NFKBIA/IRF1/TNFAIP3/BCL3/BIRC3/CXCL3 | 9 | High vs Low group |
| bta05134 | Legionellosis | 0.103448275862069 | 0.00035715969703053 | 0.00327083090964801 | NFKBIA/HSPA1A/CLK4/ARF2/CXCL3/PYCARD | 6 | High vs Low group |
| bta05210 | Colorectal cancer | 0.0760869565217391 | 0.00076959649064476 | 0.00671227014396933 | CDKN1A/FOS/GADD45A/JUN/GADD45B/GADD45G/CCND1 | 7 | High vs Low group |
| bta00650 | Butanoate metabolism | 0.148148148148148 | 0.000914622470645099 | 0.0076145602819257 | HMGCS2/ACADS/ACAT1/ACAT2 | 4 | High vs Low group |
| bta05167 | Kaposi sarcoma-associated herpesvirus infection | 0.0497737556561086 | 0.0010309324071251 | 0.00820971344804427 | CDKN1A/ZFP36/FOS/JUN/UBB/UBC/NFKBIA/RCAN1/BOLA/CCND1/CXCL3 | 11 | High vs Low group |
| bta00260 | Glycine, serine and threonine metabolism | 0.104166666666667 | 0.00108598979151092 | 0.00828781682995178 | PSPH/PSAT1/MAOA/PHGDH/SHMT2 | 5 | High vs Low group |
| bta05222 | Small cell lung cancer | 0.0707070707070707 | 0.00118840844465977 | 0.00870665555245471 | CDKN1A/GADD45A/GADD45B/GADD45G/NFKBIA/BIRC3/CCND1 | 7 | High vs Low group |
| bta05223 | Non-small cell lung cancer | 0.0810810810810811 | 0.0013173707796498 | 0.00928026379186498 | CDKN1A/GADD45A/GADD45B/GADD45G/RASSF5/CCND1 | 6 | High vs Low group |
| bta00330 | Arginine and proline metabolism | 0.0980392156862745 | 0.00143208937686959 | 0.0097147583460159 | SAT1/ODC1/MAOA/P4HA1/OAT | 5 | High vs Low group |
| bta05169 | Epstein-Barr virus infection | 0.0466101694915254 | 0.00174735111019664 | 0.0113152039627608 | CDKN1A/GADD45A/JUN/GADD45B/HES1/GADD45G/NFKBIA/BOLA/CCND2/TNFAIP3/CCND1 | 11 | High vs Low group |
| bta05220 | Chronic myeloid leukemia | 0.0759493670886076 | 0.0018481114395182 | 0.0113152039627608 | CDKN1A/GADD45A/GADD45B/GADD45G/NFKBIA/CCND1 | 6 | High vs Low group |
| bta00270 | Cysteine and methionine metabolism | 0.0925925925925926 | 0.00185335237321081 | 0.0113152039627608 | GCLM/TST/PSAT1/PHGDH/CDO1 | 5 | High vs Low group |
| bta04915 | Estrogen signaling pathway | 0.0575539568345324 | 0.0020324219670847 | 0.0120081977002627 | FOS/JUN/HBEGF/HSPA1A/KRT14/KRT17/KRT19/KRT42 | 8 | High vs Low group |
| bta00630 | Glyoxylate and dicarboxylate metabolism | 0.117647058823529 | 0.00221058613999032 | 0.0124113139576266 | ACAT1/SHMT2/ACAT2/PCCA | 4 | High vs Low group |
| bta01524 | Platinum drug resistance | 0.0731707317073171 | 0.00223617639408963 | 0.0124113139576266 | CDKN1A/GSTO1/MGST3/BIRC3/GSTA1/MGST1 | 6 | High vs Low group |
| bta03320 | PPAR signaling pathway | 0.0714285714285714 | 0.0025270667345419 | 0.0136133006752412 | UBC/HMGCS2/PLIN2/FABP5/ANGPTL4/PPARG | 6 | High vs Low group |
| bta04064 | NF-kappa B signaling pathway | 0.0614035087719298 | 0.00267677369579645 | 0.014007778137401 | GADD45A/GADD45B/GADD45G/NFKBIA/TNFAIP3/BIRC3/CXCL3 | 7 | High vs Low group |
| bta04928 | Parathyroid hormone synthesis, secretion and action | 0.0608695652173913 | 0.00281170940078662 | 0.0143051881794407 | EGR1/CDKN1A/FOS/JUND/SGK1/HBEGF/NR4A2 | 7 | High vs Low group |
| bta05213 | Endometrial cancer | 0.0833333333333333 | 0.00295799056134646 | 0.0146426844288559 | CDKN1A/GADD45A/GADD45B/GADD45G/CCND1 | 5 | High vs Low group |
| bta04380 | Osteoclast differentiation | 0.0526315789473684 | 0.00353703085269212 | 0.0168499897311964 | FOSB/FOS/JUNB/JUND/JUN/NFKBIA/ACP5/PPARG | 8 | High vs Low group |
| bta05207 | Chemical carcinogenesis - receptor activation | 0.045045045045045 | 0.0035878857444875 | 0.0168499897311964 | FOS/KLF4/JUN/GSTO1/MGST3/BCL6/DLL1/GSTA1/CCND1/MGST1 | 10 | High vs Low group |
| bta05226 | Gastric cancer | 0.051948051948052 | 0.00382986464483363 | 0.0175367486368698 | CDKN1A/GADD45A/GADD45B/GADD45G/WNT7B/WNT10A/CCND1/WNT3A | 8 | High vs Low group |
| bta00900 | Terpenoid backbone biosynthesis | 0.142857142857143 | 0.00464527532987435 | 0.0203332891646453 | HMGCS2/ACAT1/ACAT2 | 3 | High vs Low group |
| bta01522 | Endocrine resistance | 0.0631578947368421 | 0.00466263354982383 | 0.0203332891646453 | CDKN1A/FOS/JUN/HBEGF/DLL1/CCND1 | 6 | High vs Low group |
| bta00980 | Metabolism of xenobiotics by cytochrome P450 | 0.0735294117647059 | 0.00508381119170211 | 0.0216544222119013 | GSTO1/MGST3/ADH5/GSTA1/MGST1 | 5 | High vs Low group |
| bta05031 | Amphetamine addiction | 0.072463768115942 | 0.00540974431102787 | 0.0225190313425562 | FOSB/FOS/JUN/MAOA/SIRT1 | 5 | High vs Low group |
| bta05166 | Human T-cell leukemia virus 1 infection | 0.0420168067226891 | 0.00583763039511167 | 0.0237601798537878 | EGR1/CDKN1A/ZFP36/FOS/JUN/ETS2/NFKBIA/BOLA/CCND2/CCND1 | 10 | High vs Low group |
| bta00071 | Fatty acid degradation | 0.0888888888888889 | 0.00617703827413448 | 0.0245950723043341 | ACADS/ACAT1/ADH5/ACAT2 | 4 | High vs Low group |
| bta05205 | Proteoglycans in cancer | 0.0439024390243902 | 0.00664516176266954 | 0.0254906846607318 | CDKN1A/ACTG1/ACTB/DDX5/HBEGF/WNT7B/WNT10A/CCND1/WNT3A | 9 | High vs Low group |
| bta00380 | Tryptophan metabolism | 0.0869565217391304 | 0.0066803173593642 | 0.0254906846607318 | AOX1/MAOA/ACAT1/ACAT2 | 4 | High vs Low group |
| bta00750 | Vitamin B6 metabolism | 0.25 | 0.00702301571155407 | 0.0262514443353471 | AOX1/PSAT1 | 2 | High vs Low group |
| bta05218 | Melanoma | 0.0675675675675676 | 0.00726365150355822 | 0.0266079023498764 | CDKN1A/GADD45A/GADD45B/GADD45G/CCND1 | 5 | High vs Low group |
| bta04625 | C-type lectin receptor signaling pathway | 0.0566037735849057 | 0.00790137086748511 | 0.0283764402671292 | JUN/NFKBIA/IRF1/LSP1/BCL3/PYCARD | 6 | High vs Low group |
| bta04141 | Protein processing in endoplasmic reticulum | 0.0454545454545455 | 0.00843676954201049 | 0.0296062753352487 | PPP1R15A/HSPA5/DDIT3/DNAJB1/HSPA1A/HERPUD1/CRYAB/HSPH1 | 8 | High vs Low group |
| bta05212 | Pancreatic cancer | 0.0649350649350649 | 0.008567103236378 | 0.0296062753352487 | CDKN1A/GADD45A/GADD45B/GADD45G/CCND1 | 5 | High vs Low group |
| bta05214 | Glioma | 0.0641025641025641 | 0.00903533906728496 | 0.0306461792925455 | CDKN1A/GADD45A/GADD45B/GADD45G/CCND1 | 5 | High vs Low group |
| bta04310 | Wnt signaling pathway | 0.0446927374301676 | 0.00929507275538446 | 0.0307626924880588 | JUN/CCND2/WNT7B/SIAH1/WNT10A/SIRT1/CCND1/WNT3A | 8 | High vs Low group |
| bta04137 | Mitophagy - animal | 0.0545454545454545 | 0.00940560482968235 | 0.0307626924880588 | JUN/UBB/UBC/CITED2/GABARAPL1/SIAH1 | 6 | High vs Low group |
| bta04530 | Tight junction | 0.0444444444444444 | 0.00959518199631263 | 0.0308321637554644 | CLDN4/JUN/ACTG1/ACTB/CLDN1/PARD6B/CLDN3/CCND1 | 8 | High vs Low group |
| bta04550 | Signaling pathways regulating pluripotency of stem cells | 0.0482758620689655 | 0.00985861625593208 | 0.0311324723871539 | KLF4/ID2/ID3/WNT7B/WNT10A/WNT3A/LIFR | 7 | High vs Low group |
| bta04010 | MAPK signaling pathway | 0.0366666666666667 | 0.0104996108745726 | 0.0325946885312335 | DUSP1/FOS/GADD45A/JUND/JUN/GADD45B/NR4A1/DDIT3/GADD45G/DUSP6/HSPA1A | 11 | High vs Low group |
| bta05132 | Salmonella infection | 0.037593984962406 | 0.0122741478775743 | 0.0374684514157532 | FOS/JUN/ACTG1/ACTB/NFKBIA/TUBB4B/AHNAK2/ARF2/BIRC3/PYCARD | 10 | High vs Low group |
| bta04670 | Leukocyte transendothelial migration | 0.0508474576271186 | 0.0130094483017598 | 0.0390620190596413 | CLDN4/ACTG1/ACTB/CLDN1/CLDN3/RASSF5 | 6 | High vs Low group |
| bta05165 | Human papillomavirus infection | 0.0338983050847458 | 0.0137081124612608 | 0.0404959519229097 | CDKN1A/HES1/IRF1/ATP6V1B1/PTGER4/BOLA/PARD6B/CCND2/WNT7B/WNT10A/CCND1/WNT3A | 12 | High vs Low group |
| bta04921 | Oxytocin signaling pathway | 0.0448717948717949 | 0.014327271452805 | 0.0410023886971722 | CDKN1A/FOS/JUN/ACTG1/ACTB/RCAN1/CCND1 | 7 | High vs Low group |
| bta05135 | Yersinia infection | 0.0448717948717949 | 0.014327271452805 | 0.0410023886971722 | FOS/JUN/ACTG1/ACTB/NFKBIA/BAIAP2/PYCARD | 7 | High vs Low group |
| bta00130 | Ubiquinone and other terpenoid-quinone biosynthesis | 0.166666666666667 | 0.0158565148822976 | 0.0446807059031544 | COQ2/NQO1 | 2 | High vs Low group |
| bta05162 | Measles | 0.0432098765432099 | 0.0172985298754568 | 0.0480054896065309 | FOS/JUN/NFKBIA/HSPA1A/CCND2/TNFAIP3/CCND1 | 7 | High vs Low group |
| bta05417 | Lipid and atherosclerosis | 0.0371900826446281 | 0.0182555865661241 | 0.0499052955617533 | FOS/JUN/HSPA5/DDIT3/NFKBIA/HSPA1A/CXCL3/PPARG/PYCARD | 9 | High vs Low group |
| bta04510 | Focal adhesion | 0.0392156862745098 | 0.019177615766121 | 0.0516548783793353 | JUN/ACTG1/ACTB/ZYX/CCND2/BIRC3/EMP1/CCND1 | 8 | High vs Low group |
| bta05204 | Chemical carcinogenesis - DNA adducts | 0.0606060606060606 | 0.0229454692069323 | 0.060907881647692 | GSTO1/MGST3/GSTA1/MGST1 | 4 | High vs Low group |
| bta00350 | Tyrosine metabolism | 0.0789473684210526 | 0.0240584163227378 | 0.0629498412053591 | AOX1/MAOA/ADH5 | 3 | High vs Low group |
| bta05323 | Rheumatoid arthritis | 0.0480769230769231 | 0.0280988969752523 | 0.0724864058368257 | FOS/JUN/ATP6V1B1/ACP5/CXCL3 | 5 | High vs Low group |
| bta01230 | Biosynthesis of amino acids | 0.0540540540540541 | 0.0331548535397816 | 0.0832398506130124 | PSPH/PSAT1/PHGDH/SHMT2 | 4 | High vs Low group |
| bta05219 | Bladder cancer | 0.0697674418604651 | 0.033176342758692 | 0.0832398506130124 | CDKN1A/HBEGF/CCND1 | 3 | High vs Low group |
| bta00910 | Nitrogen metabolism | 0.111111111111111 | 0.0344739994957155 | 0.0853268266323542 | CA13/CA1 | 2 | High vs Low group |
| bta00620 | Pyruvate metabolism | 0.0666666666666667 | 0.0372589624848804 | 0.090990308384129 | ACAT1/ADH5/ACAT2 | 3 | High vs Low group |
| bta03018 | RNA degradation | 0.0512820512820513 | 0.0391095132045818 | 0.0930287805549861 | TOB1/BTG1/BTG2/DCPS | 4 | High vs Low group |
| bta05133 | Pertussis | 0.0512820512820513 | 0.0391095132045818 | 0.0930287805549861 | FOS/JUN/IRF1/PYCARD | 4 | High vs Low group |
| bta00983 | Drug metabolism - other enzymes | 0.0506329113924051 | 0.0406875692391917 | 0.0943322724532891 | GSTO1/MGST3/GSTA1/MGST1 | 4 | High vs Low group |
| bta05140 | Leishmaniasis | 0.0506329113924051 | 0.0406875692391917 | 0.0943322724532891 | FOS/JUN/NFKBIA/MARCKSL1 | 4 | High vs Low group |
| bta01240 | Biosynthesis of cofactors | 0.0387096774193548 | 0.0423043787315204 | 0.0968547618326913 | GCLM/PSAT1/RFK/COQ2/SHMT2/NQO1 | 6 | High vs Low group |
| bta05030 | Cocaine addiction | 0.0625 | 0.0438412604730552 | 0.0991342342080779 | FOSB/JUN/MAOA | 3 | High vs Low group |
| bta04934 | Cushing syndrome | 0.0379746835443038 | 0.0457170989298855 | 0.102115214554558 | CDKN1A/NR4A1/WNT7B/WNT10A/CCND1/WNT3A | 6 | High vs Low group |
| bta05416 | Viral myocarditis | 0.0481927710843374 | 0.0473576914044096 | 0.104505241653358 | ACTG1/ACTB/BOLA/CCND1 | 4 | High vs Low group |
| bta03272 | Virion - Hepatitis viruses | 0.06 | 0.04852982244147 | 0.105816906075386 | CLDN4/CLDN1/CLDN3 | 3 | High vs Low group |
| ID | Description | RichFactor | pvalue | qvalue | geneID | Count |  |
| bta05014 | Amyotrophic lateral sclerosis | 0.370098039215686 | 1.4118058421814e-23 | 2.40061558894155e-21 | ND4L/ATP8/CAT/SDHC/HSPA5/TUBB4B/TUBA1B/UQCRC2/NDUFS7/FUS/SDHD/TUBA4A/NDUFS2/NDUFV1/KIF5B/NDUFB3/NDUFAB1/GPX8/VDAC1/KLC3/TNFRSF1A/TUBA1A/UQCRC1/CHCHD10/CYC1/UQCR10/ATP5F1B/ATP5F1C/ATP5F1A/COX7A2L/GPX1/ATP5MC3/COX6A1/ATP5F1D/PSMA7/ND5/COX5B/ACTG1/UQCRH/ACTB/ND3/COX4I1/ND1/ND4/HNRNPA1/PFN1/COX1/ND2/COX2/ATP6/COX3/CYTB/DDIT3/NDUFA9/NDUFB9/ATP5PO/PSMD7/NDUFA1/NDUFA13/COX7A1/NRG1/MATR3/UQCRFS1/PSMC3/ATXN2/ATF4/NDUFC2/NDUFS5/ALYREF/NDUFA10/ATP5PF/COX7B/NDUFS1/UQCR11/PSMD12/ULK1/COX6B1/MAP1LC3A/NDUFA8/CYCS/PSMC5/SRSF7/PSMB2/RAC1/UQCRB/UQCRQ/NDUFS8/CCS/NDUFV2/SDHA/RANBP2/RAB5A/MAP2K6/NDUFB7/CASP3/MAPK13/NDUFA4/NXT1/PSMD8/NDUFS3/PPP3R1/MAP2K3/SRSF3/TUBB6/PSMD11/NUP98/TUBA1C/NUP85/PSMB5/BID/ATXN2L/PIK3R4/DCTN4/SEC13/NUP50/SIGMAR1/PSMC1/ND6/PSMB7/PSMA2/ADRM1/NUP62/DERL1/PSMD6/WIPI2/PSMD1/DAXX/PSMA5/PSMC4/PSMD2/BCL2L1/HNRNPA3/EIF2S1/NUP93/TP53/TUBB/ANXA11/SETX/NUP88/SEH1L/MAP1LC3B/PSMA3/VAPB/ANXA7/RAB8A/PSMD3/PSMD14/COX8B/RB1CC1/DCTN6/ACTR10 | 151 | Adult vs Newborn |
| bta05012 | Parkinson disease | 0.405940594059406 | 2.26923861641241e-23 | 2.40061558894155e-21 | MAOA/ND4L/ATP8/NFE2L2/TXN2/SDHC/HSPA5/TUBB4B/TUBA1B/UQCRC2/DUSP1/NDUFS7/SDHD/TUBA4A/NDUFS2/NDUFV1/ITPR2/KIF5B/GNAI2/NDUFB3/NDUFAB1/VDAC1/KLC3/TUBA1A/SLC39A6/UQCRC1/CALM2/CYC1/UQCR10/ATP5F1B/ATP5F1C/ATP5F1A/COX7A2L/PARK7/ATP5MC3/COX6A1/ATP5F1D/PSMA7/ND5/COX5B/UQCRH/ND3/COX4I1/ND1/ND4/RPS27A/CALM1/COX1/UBB/ND2/COX2/ATP6/COX3/CYTB/DDIT3/NDUFA9/NDUFB9/SLC39A10/ATP5PO/PSMD7/CAMK2D/NDUFA1/NDUFA13/COX7A1/UQCRFS1/PSMC3/ATF4/NDUFC2/NDUFS5/NDUFA10/ATP5PF/COX7B/NDUFS1/UBE2L3/UQCR11/PSMD12/COX6B1/NDUFA8/CYCS/PSMC5/SLC25A5/PSMB2/UQCRB/UQCRQ/NDUFS8/UBE2G1/NDUFV2/SDHA/NDUFB7/PPIF/CASP3/NDUFA4/PSMD8/SLC25A4/TRAP1/NDUFS3/TUBB6/PSMD11/TUBA1C/GNAI1/PSMB5/PSMC1/ND6/PSMB7/PSMA2/ADRM1/PSMD6/PSMD1/DAXX/PSMA5/PSMC4/PSMD2/BCL2L1/EIF2S1/TP53/TUBB/GNAI3/PSMA3/PSMD3/PSMD14/KEAP1/COX8B/SLC39A7 | 123 | Adult vs Newborn |
| bta05016 | Huntington disease | 0.36283185840708 | 1.52964670697754e-18 | 1.07880346702627e-16 | PPARG/ND4L/ATP8/SDHC/TUBB4B/TUBA1B/UQCRC2/NDUFS7/SDHD/TUBA4A/NDUFS2/NDUFV1/KIF5B/NDUFB3/NDUFAB1/GPX8/VDAC1/KLC3/TUBA1A/UQCRC1/AP2A2/CYC1/CLTB/UQCR10/ATP5F1B/ATP5F1C/ATP5F1A/COX7A2L/GPX1/ATP5MC3/COX6A1/ATP5F1D/PSMA7/ND5/COX5B/UQCRH/ND3/COX4I1/ND1/ND4/COX1/ND2/COX2/ATP6/COX3/CYTB/NDUFA9/NDUFB9/ATP5PO/PSMD7/NDUFA1/NDUFA13/COX7A1/UQCRFS1/HDAC1/PSMC3/NDUFC2/NDUFS5/NDUFA10/ATP5PF/COX7B/NDUFS1/UQCR11/PSMD12/ULK1/COX6B1/NDUFA8/CLTC/CYCS/PSMC5/SLC25A5/SOD2/PSMB2/UQCRB/UQCRQ/POLR2F/NDUFS8/NDUFV2/SDHA/NDUFB7/PPIF/CASP3/POLR2K/NDUFA4/PSMD8/SLC25A4/NDUFS3/TUBB6/PSMD11/HDAC2/TUBA1C/POLR2J/PSMB5/PIK3R4/DCTN4/CREBBP/PSMC1/ND6/POLR2D/PSMB7/POLR2E/EP300/PSMA2/ADRM1/PSMD6/WIPI2/PSMD1/POLR2A/MAP2K7/PSMA5/PSMC4/PSMD2/TP53/TUBB/POLR2C/PSMA3/PSMD3/PSMD14/COX8B/RB1CC1/DCTN6/ACTR10/POLR2B | 123 | Adult vs Newborn |
| bta05020 | Prion disease | 0.374172185430464 | 2.78712615797109e-18 | 1.47424304671629e-16 | ND4L/ATP8/CAV1/SDHC/HSPA5/TUBB4B/TUBA1B/UQCRC2/NDUFS7/SDHD/TUBA4A/NDUFS2/NDUFV1/ITPR2/KIF5B/NDUFB3/NDUFAB1/VDAC1/KLC3/LAMC1/PIK3R2/TUBA1A/UQCRC1/CYC1/HSPA8/UQCR10/ATP5F1B/ATP5F1C/ATP5F1A/COX7A2L/ATP5MC3/COX6A1/ATP5F1D/PSMA7/ND5/COX5B/UQCRH/ND3/COX4I1/ND1/ND4/COX1/ND2/COX2/ATP6/COX3/CYTB/DDIT3/NDUFA9/NDUFB9/ATP5PO/PSMD7/NDUFA1/NDUFA13/COX7A1/UQCRFS1/PSMC3/ATF4/NDUFC2/STIP1/NDUFS5/NDUFA10/ATP5PF/COX7B/NDUFS1/UQCR11/PSMD12/COX6B1/NDUFA8/CYCS/PSMC5/SLC25A5/PSMB2/RAC1/UQCRB/UQCRQ/NDUFS8/RAC2/NDUFV2/SDHA/NDUFB7/PPIF/CASP3/MAPK13/NDUFA4/PSMD8/SLC25A4/NDUFS3/PPP3R1/TUBB6/PSMD11/CYBA/TUBA1C/PSMB5/CSNK2A2/PSMC1/ND6/PSMB7/PSMA2/ADRM1/PSMD6/PSMD1/GSK3B/PSMA5/PSMC4/PSMD2/EIF2S1/TUBB/PSMA3/PSMD3/PSMD14/COX8B/EGR1 | 113 | Adult vs Newborn |
| bta05208 | Chemical carcinogenesis - reactive oxygen species | 0.386363636363636 | 9.75673992044736e-18 | 4.12864152423141e-16 | ND4L/ATP8/MGST1/NFE2L2/GSTA1/NFKBIA/GSTM3/CAT/SDHC/MGST3/EPHX2/UQCRC2/NDUFS7/SDHD/NDUFS2/NDUFV1/NDUFB3/NDUFAB1/VDAC1/PIK3R2/UQCRC1/GSTO1/FOS/CYC1/UQCR10/ATP5F1B/ATP5F1C/ATP5F1A/COX7A2L/ATP5MC3/COX6A1/ATP5F1D/ND5/COX5B/UQCRH/ND3/COX4I1/ND1/ND4/COX1/ND2/COX2/ATP6/COX3/CYTB/NQO1/NDUFA9/ABL1/NDUFB9/ATP5PO/NDUFA1/NDUFA13/COX7A1/JUN/UQCRFS1/PRKD2/KRAS/NDUFC2/AKR1A1/NDUFS5/NDUFA10/ATP5PF/COX7B/NDUFS1/UQCR11/COX6B1/NDUFA8/SLC25A5/SLC26A2/SOD2/RAC1/EPHX1/UQCRB/UQCRQ/NDUFS8/NDUFV2/SDHA/HRAS/NDUFB7/PPIF/PTK2/MGST2/MAPK13/NDUFA4/GRB2/NRAS/SLC25A4/NDUFS3/CYBA/GSTT1/ARAF/VEGFA/ND6/ACP1/MAP2K2/MAP2K7/MAP2K1/PTPN11/KEAP1/COX8B/RELA/NFKB1 | 102 | Adult vs Newborn |
| bta05010 | Alzheimer disease | 0.333333333333333 | 2.37764734863885e-17 | 8.38433538730542e-16 | ND4L/ATP8/ADAM10/SDHC/TUBB4B/TUBA1B/UQCRC2/NDUFS7/SDHD/TUBA4A/NDUFS2/NDUFV1/WNT11/ITPR2/KIF5B/NDUFB3/NDUFAB1/WNT7B/VDAC1/KLC3/TNFRSF1A/PIK3R2/TUBA1A/SLC39A6/UQCRC1/EIF2AK2/CALM2/CYC1/UQCR10/ATP5F1B/ATP5F1C/ATP5F1A/COX7A2L/ATP5MC3/COX6A1/ATP5F1D/PSMA7/ND5/COX5B/UQCRH/ND3/COX4I1/ND1/ND4/GAPDH/CALM1/COX1/ND2/COX2/ATP6/COX3/CYTB/DDIT3/NDUFA9/NDUFB9/SLC39A10/ATP5PO/PSMD7/NDUFA1/NDUFA13/COX7A1/UQCRFS1/PSMC3/ATF4/KRAS/NDUFC2/CSF1/NDUFS5/NDUFA10/ATP5PF/COX7B/NDUFS1/UQCR11/PSMD12/ULK1/COX6B1/NDUFA8/CYCS/PSMC5/SLC25A5/PSMB2/UQCRB/LRP5/UQCRQ/NDUFS8/NDUFV2/SDHA/HRAS/NDUFB7/PPIF/CASP3/NDUFA4/APP/PSMD8/AXIN1/NRAS/SLC25A4/NDUFS3/PPP3R1/WNT6/TUBB6/PSMD11/TUBA1C/APC/CDK5/PSMB5/ARAF/BID/PIK3R4/CSNK2A2/PSMC1/ND6/PSMB7/MAP2K2/PSMA2/WNT10A/ADRM1/PSMD6/WIPI2/PSMD1/GSK3B/MAP2K7/PSMA5/PSMC4/PSMD2/ATP2A2/MAP2K1/EIF2S1/TUBB/PPID/NAE1/PSMA3/PSMD3/APH1A/PSMD14/COX8B/RB1CC1/RELA/SLC39A7/NFKB1/FADD | 141 | Adult vs Newborn |
| bta00190 | Oxidative phosphorylation | 0.432926829268293 | 1.0357109249895e-15 | 3.13049467553219e-14 | ND4L/ATP8/PPA2/SDHC/PPA1/UQCRC2/NDUFS7/SDHD/NDUFS2/NDUFV1/NDUFB3/ATP6V1B1/NDUFAB1/UQCRC1/ATP5MF/CYC1/UQCR10/ATP5F1B/ATP5F1C/ATP5F1A/COX7A2L/ATP5MC3/COX6A1/ATP5F1D/ND5/COX5B/UQCRH/ND3/COX4I1/ND1/ND4/COX1/ND2/COX2/ATP6/COX3/CYTB/NDUFA9/NDUFB9/ATP5PO/NDUFA1/NDUFA13/COX7A1/ATP6V1G1/UQCRFS1/ATP5MG/NDUFC2/ATP5ME/NDUFS5/NDUFA10/ATP5PF/COX7B/NDUFS1/UQCR11/COX6B1/NDUFA8/ATP6V1F/CYCS/UQCRB/UQCRQ/NDUFS8/NDUFV2/SDHA/NDUFB7/NDUFA4/NDUFS3/ATP6V0D1/ND6/ATP6V1H/ATP6V1E1/COX8B | 71 | Adult vs Newborn |
| bta05415 | Diabetic cardiomyopathy | 0.369098712446352 | 8.4812882257737e-14 | 2.24307754392173e-12 | ND4L/ATP8/SDHC/UQCRC2/NDUFS7/SDHD/NDUFS2/NDUFV1/NDUFB3/NDUFAB1/VDAC1/PIK3R2/PPP1CC/UQCRC1/CYC1/UQCR10/ATP5F1B/ATP5F1C/ATP5F1A/COX7A2L/ATP5MC3/COX6A1/ATP5F1D/ND5/COX5B/UQCRH/ND3/COX4I1/ND1/ND4/GAPDH/COX1/ND2/COX2/ATP6/COX3/CYTB/NDUFA9/NDUFB9/ATP5PO/CAMK2D/NDUFA1/NDUFA13/COX7A1/UQCRFS1/PDK3/NDUFC2/NDUFS5/NDUFA10/ATP5PF/COX7B/NDUFS1/SLC2A1/UQCR11/COX6B1/NDUFA8/PARP1/MPC2/SLC25A5/RAC1/UQCRB/UQCRQ/PDHA1/NDUFS8/RAC2/NDUFV2/SDHA/NDUFB7/PPIF/MAPK13/NDUFA4/SLC25A4/PDHB/NDUFS3/PTPA/CYBA/CPT2/SMAD2/PDK4/PRKCZ/ND6/GSK3B/ATP2A2/COX8B/RELA/NFKB1 | 86 | Adult vs Newborn |
| bta01200 | Carbon metabolism | 0.452991452991453 | 5.05645754451582e-13 | 1.18871107186863e-11 | ACADS/ME1/ACAT1/SHMT1/SHMT2/GLYCTK/ADH5/PSPH/IDH3B/ESD/HIBCH/PCCA/CAT/SDHC/MMUT/IDH1/TKT/IDH3G/DLD/PSAT1/ACSS2/ECHS1/SDHD/SUCLG1/IDH2/SUCLG2/GCSH/MDH2/GAPDH/PCCB/FH/PKM/FBP2/PFKL/TPI1/TKFC/ACO2/PGLS/PDHA1/SDHA/ACAT2/PGP/PDHB/OGDH/HK1/DLAT/ME2/ALDH6A1/PRPS1/GPI/RPE/PGD/IDH3A | 53 | Adult vs Newborn |
| bta04714 | Thermogenesis | 0.35 | 5.85944877649285e-13 | 1.23973600428954e-11 | PPARG/ND4L/ATP8/SMARCD2/SDHC/UQCRC2/SIRT6/NDUFS7/SDHD/SMARCA2/NDUFS2/NDUFV1/NDUFB3/NDUFAB1/UQCRC1/ATP5MF/CYC1/UQCR10/ATP5F1B/ATP5F1C/ATP5F1A/COX7A2L/ATP5MC3/COX6A1/ATP5F1D/ND5/COX5B/ACTG1/UQCRH/ACTB/ND3/COX4I1/ND1/ND4/COX1/ND2/COX2/ATP6/COX3/CYTB/NDUFA9/NDUFB9/ATP5PO/NDUFA1/NDUFA13/COX7A1/ACSL3/UQCRFS1/ATP5MG/KRAS/NDUFC2/ATP5ME/NDUFS5/NDUFA10/ATP5PF/COX7B/NDUFS1/SMARCB1/UQCR11/SMARCC1/COX6B1/NDUFA8/UQCRB/UQCRQ/SMARCE1/NDUFS8/NDUFV2/SDHA/ARID1A/HRAS/NDUFB7/NDUFAF3/MAPK13/NDUFA4/GRB2/NRAS/CPT1A/NDUFS3/MAP2K3/SMARCA4/KDM1A/CPT2/COA6/ND6/SMARCC2/PNPLA2/RHEB/COX19/RPS6KB1/COA1/COX8B | 91 | Adult vs Newborn |
| bta03082 | ATP-dependent chromatin remodeling | 0.439024390243902 | 1.44874579163469e-12 | 2.7865828145318e-11 | SMARCD2/BAZ2B/MORF4L2/SMARCA2/YY1/SMARCA1/ACTG1/ACTB/BAZ1A/HDAC1/MBD3/SMARCB1/SMARCC1/BPTF/RSF1/CHD3/MBD2/PBRM1/SMARCE1/EPC1/ARID1A/POLE3/PHF6/EPC2/MTA1/MTA3/SMARCA4/HDAC2/CHD4/RBBP7/CDK2AP1/BRD9/UCHL5/CHRAC1/RUVBL2/YEATS4/BAZ2A/RBBP4/CDK2AP2/MRGBP/TRRAP/EP400/MCRS1/BAZ1B/RUVBL1/SMARCC2/BRD8/BCL7C/PHF10/INO80C/KAT5/INO80B/VPS72/INO80 | 54 | Adult vs Newborn |
| bta05132 | Salmonella infection | 0.330827067669173 | 4.63739949315551e-11 | 8.17646752740577e-10 | ARPC5L/NFKBIA/LY96/AHNAK2/TXN2/TUBB4B/TUBA1B/MYC/DYNLL1/TUBA4A/KIF5B/ARPC5/KLC3/TNFRSF1A/PAK1/TUBA1A/FOS/HSP90B1/ARF1/ARPC3/MYL12A/ACTG1/ACTB/GAPDH/HSP90AA1/PFN1/HSP90AB1/ANXA2/CDC42/JUN/PYCARD/CYCS/DYNLT3/BRK1/WASL/RAC1/MYL12B/AHNAK/RAB5A/HRAS/CD14/MAP2K6/ARPC1A/CASP3/MAPK13/SNX9/EXOC5/BIRC2/KPNA3/IL18/MAP2K3/CYFIP1/TUBB6/ROCK2/ARPC1B/TUBA1C/DYNC1H1/ARF2/RIPK1/PKN1/ACTR3/EXOC7/MYL9/DYNC1LI1/DYNLRB1/ACBD3/DCTN4/FYCO1/DYNC1LI2/M6PR/GCC2/MYD88/MAP2K2/ACTR2/CSE1L/RAB7A/MAP2K7/MAP2K1/FLNA/TUBB/IRAK4/RRAS/DCTN6/RELA/ACTR10/NFKB1/FADD/DNM2 | 88 | Adult vs Newborn |
| bta03050 | Proteasome | 0.571428571428571 | 1.85219688739885e-10 | 3.0145066750378e-09 | PSMB8/PSME1/PSMA7/PSMD7/PSMC3/PSMD12/PSMC5/PSMB2/PSMF1/PSMD8/PSME2/PSMD11/PSMB5/PSMC1/PSMB7/PSMB10/PSMA2/ADRM1/PSME3/PSMD6/PSMD1/PSMA5/PSMC4/PSMD2/PSME4/PSMA3/PSMD3/PSMD14 | 28 | Adult vs Newborn |
| bta04141 | Protein processing in endoplasmic reticulum | 0.357954545454545 | 7.90945475090969e-10 | 1.19533865032545e-08 | NFE2L2/HSPA5/PDIA6/DNAJA1/OSTC/CKAP4/DDOST/PDIA3/HSP90B1/HSPA8/ERP29/CALR/HSP90AA1/HSP90AB1/DDIT3/PDIA4/PPP1R15A/RAD23A/ATF4/DERL2/MBTPS1/LMAN2/KRTCAP2/RPN2/CRYAB/BCAP31/SEC62/CUL1/UBE2G1/RRBP1/HSPH1/CANX/DNAJB11/P4HB/HERPUD1/SEL1L/DNAJC1/DNAJC5/UBE2D3/LMAN1/SEC63/HSPA4L/STT3A/RNF5/UBE4B/SEC13/SEC31A/BAG1/DERL1/UBE2D2/STT3B/TMEM258/DNAJC3/MAP2K7/DNAJC10/RAD23B/EIF2S1/MAN1A1/ERLEC1/MAN1A2/HSPBP1/NSFL1C/SEC61A1 | 63 | Adult vs Newborn |
| bta04932 | Non-alcoholic fatty liver disease | 0.353932584269663 | 1.33671452977807e-09 | 1.88547102095012e-08 | PPARG/SDHC/UQCRC2/NDUFS7/SDHD/NDUFS2/NDUFV1/NDUFB3/NDUFAB1/TNFRSF1A/PIK3R2/UQCRC1/FOS/CYC1/UQCR10/COX7A2L/COX6A1/COX5B/UQCRH/COX4I1/COX1/COX2/COX3/CYTB/DDIT3/NDUFA9/NDUFB9/NDUFA1/NDUFA13/COX7A1/CDC42/JUN/UQCRFS1/ATF4/NDUFC2/NDUFS5/NDUFA10/COX7B/NDUFS1/UQCR11/COX6B1/NDUFA8/CYCS/RAC1/UQCRB/UQCRQ/RXRA/NDUFS8/NDUFV2/SDHA/NDUFB7/CASP3/MAPK13/NDUFA4/NDUFS3/BID/GSK3A/GSK3B/ADIPOR1/EIF2S1/COX8B/RELA/NFKB1 | 63 | Adult vs Newborn |
| bta03018 | RNA degradation | 0.461538461538462 | 1.46517439062987e-09 | 1.93750034550397e-08 | TOB2/EXOSC6/BTG1/PABPC1/PFKL/LSM4/DHX36/DCPS/LSM5/LSM8/LSM3/XRN2/NUDT16/LSM2/BTG3/LSM7/EXOSC3/HSPD1/XRN1/TOB1/CNOT10/HSPA9/CNOT2/LSM1/CNOT6L/PABPC4/CNOT4/EXOSC7/EXOSC4/PATL1/CNOT7/EXOSC8/MPHOSPH6/CNOT3/PAN3/DIS3L | 36 | Adult vs Newborn |
| bta00020 | Citrate cycle (TCA cycle) | 0.645161290322581 | 3.95352522081178e-09 | 4.92048649772859e-08 | IDH3B/SDHC/IDH1/IDH3G/DLD/SDHD/SUCLG1/IDH2/SUCLG2/MDH2/FH/ACO2/PDHA1/SDHA/ACLY/PDHB/PCK2/OGDH/DLAT/IDH3A | 20 | Adult vs Newborn |
| bta03420 | Nucleotide excision repair | 0.491525423728814 | 9.67166545430797e-09 | 1.13684488673445e-07 | PCNA/RAD23A/POLR2F/POLE3/POLR2K/DDB1/RPA3/LIG1/XPC/POLR2J/POLD3/RFC5/ERCC2/CETN2/POLR2D/CUL4B/POLR2E/POLR2A/RFC1/ERCC5/RFC2/RPA2/RAD23B/POLR2C/POLD2/GTF2H1/GTF2H5/RFC3/POLR2B | 29 | Adult vs Newborn |
| bta05100 | Bacterial invasion of epithelial cells | 0.447368421052632 | 1.12764271114436e-08 | 1.25571293595577e-07 | ARPC5L/CAV1/ARPC5/SEPTIN9/PIK3R2/ITGB1/CLTB/ARPC3/ACTG1/ACTB/CDC42/CDH1/SEPTIN2/CLTC/CTTN/WASL/RAC1/ARPC1A/PTK2/ARPC1B/ARHGAP10/CD2AP/ACTR3/WASF2/PXN/CRKL/ACTR2/VCL/DOCK1/BCAR1/SHC1/ILK/CRK/DNM2 | 34 | Adult vs Newborn |
| bta03013 | Nucleocytoplasmic transport | 0.368 | 5.80233975337554e-08 | 6.1382646864657e-07 | PNN/RAN/EEF1A1/SAP18/SUMO2/UBE2I/ALYREF/XPO1/RNPS1/IPO5/RBM8A/RANBP2/NXT1/EIF4A3/MAGOH/KPNA3/THOC2/SRRM1/IPO7/NUP98/SUMO3/NUP85/DDX39B/UPF2/UPF1/SEC13/NUP50/CSE1L/THOC1/NUP62/AHCTF1/NUP93/NUP88/ACIN1/SEH1L/NMD3/DDX19A/CASC3/TMEM33/THOC5/IPO9/KPNB1/TNPO1/IPO8/THOC3/SNUPN | 46 | Adult vs Newborn |
| bta04110 | Cell cycle | 0.335365853658537 | 1.22022497838834e-07 | 1.229399602286e-06 | GADD45G/CDKN1A/CCND3/ANAPC11/GADD45B/CCND1/MYC/CCND2/YWHAQ/PCNA/ABL1/ATRX/NIPBL/HDAC1/MDM2/MCM2/YWHAH/YWHAZ/ANAPC13/RB1/MCM4/CDK2/PDS5B/CUL1/YWHAB/MCM3/PPP2CA/HDAC2/YWHAG/E2F4/SMC1A/TFDP1/MCM7/SMAD2/MCM5/SMC3/ESCO1/PPP2R5E/CDK4/BUB3/STAG2/CREBBP/WAPL/EP300/GSK3B/SMAD4/ANAPC2/TP53/SFN/CDC16/PPP2CB/ANAPC4/CDKN1C/PPP2R5C/CDC27 | 55 | Adult vs Newborn |
| bta03030 | DNA replication | 0.555555555555556 | 1.44232908422048e-07 | 1.38712031544649e-06 | PCNA/MCM2/MCM4/MCM3/RNASEH2A/POLE3/SSBP1/RPA3/LIG1/RNASEH2B/MCM7/POLD3/MCM5/RFC5/RFC1/RFC2/RPA2/POLD2/RNASEH1/RFC3 | 20 | Adult vs Newborn |
| bta00280 | Valine, leucine and isoleucine degradation | 0.480769230769231 | 1.75724485818841e-07 | 1.61650442332206e-06 | HMGCS2/ACADS/ALDH2/ACAT1/HMGCL/HADHB/ACADSB/AOX1/HIBCH/PCCA/BCKDHA/MMUT/DLD/ACAA2/ECHS1/HADH/PCCB/IVD/HMGCS1/ALDH9A1/MCCC1/ACAT2/ACADM/BCKDHB/ALDH6A1 | 25 | Adult vs Newborn |
| bta05205 | Proteoglycans in cancer | 0.31219512195122 | 2.36880200298582e-07 | 2.08828597631644e-06 | DCN/IGF2/CDKN1A/ANK3/CAV1/CCND1/THBS1/MYC/RDX/WNT11/ITPR2/WNT7B/PAK1/PIK3R2/PPP1CC/DDX5/EIF4B/ITGB1/CD63/CD44/ACTG1/EZR/ACTB/CAMK2D/CDC42/KRAS/MDM2/CTTN/TFAP4/PPP1R12A/SLC9A1/RAC1/PTPN6/HRAS/PTK2/CASP3/MAPK13/GRB2/NRAS/WNT6/ROCK2/ERBB2/NUDT16L1/SMAD2/ITGA2/ARAF/VEGFA/PDCD4/ITGB5/PXN/MAP2K2/CTSV/ARHGEF1/WNT10A/IQGAP1/MAP2K1/GPC1/TP53/FLNA/SDC1/PTPN11/RRAS/ROCK1/RPS6KB1 | 64 | Adult vs Newborn |
| bta03015 | mRNA surveillance pathway | 0.369369369369369 | 2.68447825552995e-07 | 2.27191633415377e-06 | FUS/PPP1CC/PNN/PABPC1/SAP18/ALYREF/PCF11/RNPS1/PABPN1/GSPT1/RBM8A/NXT1/EIF4A3/PPP2CA/MAGOH/SRRM1/FIP1L1/ETF1/PAPOLA/DDX39B/UPF2/CPSF6/UPF1/PPP2R5E/PABPC4/CSTF2T/PPP2R3A/WDR33/DAZAP1/RNMT/RNGTT/SMG1/SSU72/WDR82/ACIN1/PELO/DDX19A/PPP2CB/PPP2R2A/CASC3/PPP2R5C | 41 | Adult vs Newborn |
| bta05220 | Chronic myeloid leukemia | 0.40506329113924 | 5.00532256303383e-07 | 4.0731572274081e-06 | GADD45G/CDKN1A/NFKBIA/GADD45B/CCND1/MYC/PIK3R2/ABL1/HDAC1/KRAS/MDM2/RB1/MECOM/HRAS/GRB2/NRAS/CTBP2/HDAC2/ARAF/CDK4/CTBP1/CRKL/MAP2K2/SMAD4/BCL2L1/MAP2K1/TP53/PTPN11/RELA/SHC1/CRK/NFKB1 | 32 | Adult vs Newborn |
| bta05418 | Fluid shear stress and atherosclerosis | 0.33112582781457 | 7.00313001984829e-07 | 5.4878328810507e-06 | IL1R2/MGST1/PDGFA/NFE2L2/GSTA1/CAV1/TXN2/GSTM3/MGST3/DUSP1/MEF2A/ACVR2A/TNFRSF1A/PIK3R2/GSTO1/FOS/CALM2/HSP90B1/ACTG1/ACTB/CALM1/HSP90AA1/HSP90AB1/NQO1/JUN/SUMO2/RAC1/RAC2/MAP2K6/PTK2/MGST2/MAPK13/BMPR1A/IL1R1/ASS1/CYBA/SUMO3/GSTT1/VEGFA/PRKCZ/CTSV/MAP2K7/GPC1/TP53/SDC1/GSTP1/KEAP1/BMPR2/RELA/NFKB1 | 50 | Adult vs Newborn |
| bta04520 | Adherens junction | 0.376344086021505 | 1.19109181845438e-06 | 9.00035547027558e-06 | BAIAP2/AFDN/MYL12A/ACTG1/ACTB/CDC42/ACTN4/CDH1/PTPRF/PARD3/WASL/FER/RAC1/MYL12B/PTPN6/RAC2/RAP1B/ROCK2/ERBB2/CCM2/PDCD10/LMO7/MYL9/NECTIN2/CSNK2A2/CREBBP/WASF2/ACP1/EP300/VCL/YES1/SMAD4/IQGAP1/RAP1A/ROCK1 | 35 | Adult vs Newborn |
| bta00640 | Propanoate metabolism | 0.548387096774194 | 1.61606334191572e-06 | 1.17905165780421e-05 | ACADS/HIBCH/PCCA/BCKDHA/MMUT/ECHDC1/DLD/ACSS2/ECHS1/LDHB/SUCLG1/SUCLG2/LDHA/PCCB/MLYCD/BCKDHB/ALDH6A1 | 17 | Adult vs Newborn |
| bta03430 | Mismatch repair | 0.608695652173913 | 2.54353893228932e-06 | 1.78755174330668e-05 | PCNA/SSBP1/RPA3/LIG1/POLD3/RFC5/MSH2/RFC1/RFC2/RPA2/MSH6/MSH3/POLD2/RFC3 | 14 | Adult vs Newborn |
| bta01232 | Nucleotide metabolism | 0.375 | 2.61907456917322e-06 | 1.78755174330668e-05 | AK1/NT5E/NT5C/ADK/IMPDH1/ENTPD3/XDH/CDA/DUT/AK2/NME2/APRT/NT5C3B/IMPDH2/CMPK1/NUDT16/DCTPP1/DTYMK/TYMS/ADSS1/ADSS2/RRM1/NT5C2/AK3/DGUOK/HPRT1/ADSL/NME7/GMPS/NME1/HDDC2/GMPR2/UCK1 | 33 | Adult vs Newborn |
| bta00620 | Pyruvate metabolism | 0.466666666666667 | 3.11705685277999e-06 | 2.06094877437098e-05 | ME1/ALDH2/ACAT1/ADH5/DLD/ACSS2/LDHB/LDHA/MDH2/FH/PKM/AKR1A1/ALDH9A1/PDHA1/ACAT2/PDHB/PCK2/ACYP1/DLAT/ME2/HAGH | 21 | Adult vs Newborn |
| bta04810 | Regulation of actin cytoskeleton | 0.28755364806867 | 3.34624585087635e-06 | 2.14543992352838e-05 | ARPC5L/BAIAP2/PDGFA/RDX/TMSB4/ARPC5/PAK1/PIK3R2/PPP1CC/ITGB1/ARPC3/CFL1/MYL12A/TMSB4X/ACTG1/EZR/ACTB/PFN1/PAK4/CDC42/KRAS/ARHGEF4/ACTN4/SSH3/PPP1R12A/SLC9A1/BRK1/WASL/RAC1/MYL12B/RAC2/HRAS/ITGB4/IQGAP2/ENAH/ARPC1A/PTK2/NRAS/CYFIP1/ROCK2/ARPC1B/APC/ACTR3/ITGA2/MYL9/ARAF/FGFR2/ITGB5/WASF2/PXN/CRKL/MAP2K2/ACTR2/VCL/GNG12/ARHGEF1/ITGA3/IQGAP1/MAP2K1/PIP4K2C/DOCK1/BCAR1/ARHGAP35/RRAS/ROCK1/CRK/LIMK2 | 67 | Adult vs Newborn |
| bta05225 | Hepatocellular carcinoma | 0.306818181818182 | 3.66981230305483e-06 | 2.27680323071809e-05 | IGF2/GADD45G/MGST1/CDKN1A/NFE2L2/GSTA1/GADD45B/SMARCD2/GSTM3/CCND1/MGST3/MYC/SMARCA2/WNT11/WNT7B/PIK3R2/GSTO1/ACTG1/ACTB/NQO1/KRAS/SMARCB1/SMARCC1/RB1/PBRM1/LRP5/SMARCE1/ARID1A/HRAS/MGST2/AXIN1/GRB2/NRAS/WNT6/SMARCA4/APC/SMAD2/TXNRD1/GSTT1/ARAF/CDK4/MAP2K2/WNT10A/GSK3B/SMAD4/BCL2L1/MAP2K1/TP53/SMARCC2/PHF10/RPS6KB1/GSTP1/KEAP1/SHC1 | 54 | Adult vs Newborn |
| bta05211 | Renal cell carcinoma | 0.391891891891892 | 3.76635360305355e-06 | 2.27680323071809e-05 | CDKN1A/PAK1/PIK3R2/EGLN3/PAK4/FH/CDC42/JUN/KRAS/SLC2A1/RAC1/HRAS/GRB2/NRAS/RAP1B/EGLN1/ARAF/VEGFA/CREBBP/CRKL/MAP2K2/EP300/EGLN2/CUL2/MAP2K1/PRCC/PTPN11/RAP1A/CRK | 29 | Adult vs Newborn |
| bta04919 | Thyroid hormone signaling pathway | 0.336065573770492 | 4.53345703720623e-06 | 2.66440018853348e-05 | RCAN1/ATP1B1/CCND1/MYC/ATP1A1/PIK3R2/ACTG1/ACTB/HDAC1/KRAS/MDM2/NCOR1/PFKL/SLC2A1/SLC9A1/RXRA/HRAS/NRAS/PLCD1/HDAC2/STAT1/THRA/MED27/KAT2A/MED4/CREBBP/MED30/MED13/MAP2K2/EP300/MED13L/NCOA1/GSK3B/ATP2A2/MAP2K1/TP53/MED17/RHEB/MED1/HDAC3/RXRB | 41 | Adult vs Newborn |
| bta04144 | Endocytosis | 0.281124497991968 | 4.88493249560428e-06 | 2.74749390071158e-05 | BOLA/ARPC5L/CAV1/SH3GL1/KIF5B/ARPC5/AP2A2/HSPA8/CLTB/ARF1/ARPC3/SMAP1/SPART/CDC42/SNF8/MDM2/PARD6B/EHD2/BIN1/CLTC/PARD3/WASL/RAB5A/PRKCI/HRAS/GRK2/ARPC1A/SNX6/RAB11B/SNX5/SMAP2/ACAP2/CHMP2A/VPS29/VPS36/ARPC1B/ARF2/PDCD6IP/VTA1/ACTR3/SMAD2/FGFR2/SNX1/VPS25/LDLRAP1/CHMP4B/PRKCZ/VPS26A/ACTR2/RABEP1/RAB7A/RAB10/CHMP3/EPN2/EEA1/VPS4B/ARF4/EHD1/ARF3/ARFGEF2/RAB8A/VPS26B/TSG101/ARFGEF1/SNX4/CHMP4A/CHMP6/VPS37A/HGS/DNM2 | 70 | Adult vs Newborn |
| bta04510 | Focal adhesion | 0.294117647058824 | 4.93455372217353e-06 | 2.74749390071158e-05 | PDGFA/CCND3/CAV1/LAMA3/CCND1/COL4A5/THBS1/CCND2/LAMC1/PAK1/PIK3R2/PPP1CC/ITGB1/MYL12A/ACTG1/ACTB/PAK4/VASP/CDC42/JUN/ACTN4/EMP2/ARHGAP5/PPP1R12A/VEGFB/LAMB3/RAC1/MYL12B/RAC2/HRAS/ITGB4/PTK2/ZYX/PARVA/GRB2/BIRC2/RAP1B/ROCK2/ERBB2/ITGA2/MYL9/VEGFA/EMP1/TLN1/ITGB5/PXN/CRKL/VCL/ITGA3/GSK3B/MAP2K1/FLNA/DOCK1/BCAR1/RAP1A/ARHGAP35/ROCK1/SHC1/ILK/CRK | 60 | Adult vs Newborn |
| bta04210 | Apoptosis | 0.315436241610738 | 6.76597580521959e-06 | 3.67061035586812e-05 | GADD45G/CTSC/NFKBIA/GADD45B/TUBA1B/CTSH/TUBA4A/ITPR2/TNFRSF1A/PIK3R2/TUBA1A/FOS/ACTG1/ACTB/DDIT3/JUN/ATF4/KRAS/AIFM1/MCL1/CTSF/LMNB1/PARP1/CYCS/HRAS/CASP3/NRAS/BIRC2/ENDOG/PTPN13/TUBA1C/LMNB2/RIPK1/BID/LMNA/CASP6/MAP2K2/CTSV/CTSB/DAXX/BCL2L1/MAP2K1/EIF2S1/TP53/RELA/NFKB1/FADD | 47 | Adult vs Newborn |
| bta03060 | Protein export | 0.516129032258065 | 9.32603563860393e-06 | 4.9329820088405e-05 | HSPA5/SPCS3/SEC11A/SEC11C/SEC62/SRPRA/SRP14/SEC63/GET3/SGTA/SRP72/BAG6/SRP19/GET4/SRP68/SEC61A1 | 16 | Adult vs Newborn |
| bta04530 | Tight junction | 0.294444444444444 | 1.6733933917547e-05 | 8.63548322831054e-05 | CLDN1/ARPC5L/RAB13/MICALL2/CLDN4/CCND1/AFDN/TUBA1B/RDX/YBX3/TUBA4A/ARPC5/TUBA1A/CLDN3/ITGB1/ARPC3/MYL6/MYL12A/ACTG1/EZR/ACTB/PCNA/VASP/CDC42/JUN/ACTN4/PARD6B/CTTN/PARD3/RAC1/MYL12B/RAP2C/HSPA4/PRKCI/ARPC1A/PPP2CA/ROCK2/ERBB2/ARPC1B/TUBA1C/ACTR3/MYL9/CDK4/PRKCZ/ACTR2/MAP2K7/LLGL1/RAP1A/PPP2CB/PPP2R2A/PATJ/ROCK1/RAB8A | 53 | Adult vs Newborn |
| bta04137 | Mitophagy - animal | 0.327272727272727 | 3.26465270812626e-05 | 0.000164459948454481 | SAMM50/CITED2/TAX1BP1/GABARAP/RPS27A/UBB/JUN/GABARAPL1/ATF4/KRAS/PHB2/ULK1/MAP1LC3A/TBC1D15/RAB5A/HRAS/BCL2L13/CALCOCO2/HUWE1/NRAS/BNIP3L/FKBP8/CSNK2A2/ARIH1/TOMM7/RAB7A/SIAH1/BCL2L1/EIF2S1/MTX2/TP53/MAP1LC3B/MARCHF5/RRAS/USP15/RELA | 36 | Adult vs Newborn |
| bta05230 | Central carbon metabolism in cancer | 0.371428571428571 | 3.58701146119614e-05 | 0.000176496769571707 | IDH1/MYC/SIRT6/LDHB/SLC1A5/IDH2/PIK3R2/LDHA/SIRT3/PKM/KRAS/PDK1/PFKL/SLC2A1/PDHA1/HRAS/NRAS/PDHB/GLS/ERBB2/FGFR2/HK1/SLC7A5/MAP2K2/MAP2K1/TP53 | 26 | Adult vs Newborn |
| bta00630 | Glyoxylate and dicarboxylate metabolism | 0.470588235294118 | 4.11525623344986e-05 | 0.000197886723187421 | ACAT1/SHMT1/SHMT2/GLYCTK/PCCA/CAT/MMUT/DLD/ACSS2/GCSH/GLUL/MDH2/PCCB/ACO2/ACAT2/PGP | 16 | Adult vs Newborn |
| bta05210 | Colorectal cancer | 0.33695652173913 | 6.05326308061358e-05 | 0.000284609562386744 | GADD45G/CDKN1A/GADD45B/CCND1/MYC/PIK3R2/FOS/JUN/KRAS/CYCS/RAC1/RAC2/HRAS/CASP3/AXIN1/GRB2/NRAS/RALB/APC/SMAD2/ARAF/MSH2/MAP2K2/GSK3B/SMAD4/MSH6/MAP2K1/TP53/MSH3/APPL1/RPS6KB1 | 31 | Adult vs Newborn |
| bta05169 | Epstein-Barr virus infection | 0.266949152542373 | 7.9436254779657e-05 | 0.000365370416721077 | BOLA/GADD45G/OAS1Y/CDKN1A/ISG15/CCND3/HES1/NFKBIA/GADD45B/CCND1/MYC/CCND2/ENTPD3/NCOR2/PIK3R2/EIF2AK2/PDIA3/B2M/CALR/CD44/PSMD7/JUN/HDAC1/PSMC3/IRF7/MDM2/IRF9/JAK1/PSMD12/CYCS/RB1/PSMC5/CDK2/RAC1/MAP2K6/CASP3/MAPK13/PSMD8/MAP2K3/PSMD11/HDAC2/STAT1/RIPK1/BID/CDK4/MYD88/PSMC1/IRF3/ADRM1/PSMD6/PSMD1/MAP2K7/PSMC4/PSMD2/TAPBP/TP53/SNW1/IRAK4/PSMD3/PSMD14/RELA/NFKB1/FADD | 63 | Adult vs Newborn |
| bta05163 | Human cytomegalovirus infection | 0.262948207171315 | 8.889594949116e-05 | 0.000400181094013957 | BOLA/CDKN1A/NFKBIA/CCND1/MYC/ITPR2/GNAI2/TNFRSF1A/PIK3R2/CALM2/PDIA3/B2M/CALR/GNG5/CALM1/PTGER4/GNB5/ATF4/KRAS/MDM2/GNB2/JAK1/CYCS/RB1/AKAP13/RAC1/RAC2/HRAS/MAP2K6/PTK2/CASP3/MAPK13/IL1R1/GRB2/NRAS/PPP3R1/ROCK2/GNAI1/GNB1/RIPK1/VEGFA/BID/CX3CL1/CDK4/GNA11/IRF3/PXN/CRKL/MAP2K2/EIF4EBP1/GNG12/ARHGEF1/GSK3B/TAPBP/MAP2K1/TP53/GNAI3/BCAR1/RHEB/ROCK1/RPS6KB1/RELA/CRK/NFKB1/FADD/IL10RB | 66 | Adult vs Newborn |
| bta05219 | Bladder cancer | 0.418604651162791 | 9.42978005201116e-05 | 0.000415654778608387 | CDKN1A/CCND1/THBS1/MYC/KRAS/MDM2/CDH1/RB1/HRAS/NRAS/ERBB2/ARAF/VEGFA/CDK4/MAP2K2/MAP2K1/TP53/RASSF1 | 18 | Adult vs Newborn |
| bta04140 | Autophagy - animal | 0.283236994219653 | 0.000101682416532304 | 0.000439058339913923 | DDIT4/PIK3R2/TAX1BP1/GABARAP/HMGB1/RPS27A/UBB/ATG7/GABARAPL1/KRAS/ULK1/MAP1LC3A/RRAGB/HRAS/CALCOCO2/ATG4D/PPP2CA/NRAS/RRAGA/BIRC6/PIK3R4/VMP1/STX7/RRAGC/ATG3/VAMP8/MAP2K2/CTSV/RAB7A/WIPI2/WDR45B/CTSB/SUPT20H/ATG4B/BCL2L1/MAP2K1/EIF2S1/ATG16L1/GORASP2/MAP1LC3B/PPP2CB/MTMR3/ATG5/RHEB/RRAS/RAB8A/EIF2AK4/RPS6KB1/RB1CC1 | 49 | Adult vs Newborn |
| bta05135 | Yersinia infection | 0.288461538461538 | 0.000120721873595122 | 0.000510844138791991 | ARPC5L/BAIAP2/NFKBIA/ARPC5/PIK3R2/FOS/ITGB1/ARPC3/ACTG1/ACTB/CDC42/JUN/PYCARD/WASL/RAC1/RAC2/MAP2K6/ARPC1A/PTK2/MAPK13/IL18/MAP2K3/ROCK2/ARPC1B/PKN1/ACTR3/WASF2/MYD88/IRF3/PXN/CRKL/MAP2K2/ACTR2/ARHGEF1/GSK3B/PKN2/MAP2K7/MAP2K1/DOCK1/BCAR1/IRAK4/ROCK1/RELA/CRK/NFKB1 | 45 | Adult vs Newborn |
| bta04120 | Ubiquitin mediated proteolysis | 0.285714285714286 | 0.000130822445843027 | 0.000542730889875097 | ANAPC11/CDC34/RPS27A/UBB/UBE2S/UBE2I/MDM2/UBE2L3/ANAPC13/FBXW7/UBE2Q1/RNF7/CUL1/UBE2G1/UBA2/HUWE1/DDB1/UBE2M/BIRC2/UBE2D3/BIRC6/UBE2E1/HERC2/SAE1/UBE4B/UBE2H/CUL4B/UBE2E3/UBE2N/CUL2/UBE2D2/SIAH1/TRIP12/PRPF19/ANAPC2/UBE2Z/UBE2F/CDC16/UBE2K/ANAPC4/UBR5/KEAP1/CDC27/FBXW8/CUL5/RCHY1 | 46 | Adult vs Newborn |
| bta03250 | Viral life cycle - HIV-1 | 0.346666666666667 | 0.000137167907567487 | 0.000558112336458805 | MX1/MLLT3/RAN/PSIP1/SMARCB1/XPO1/APOBEC3H/RANBP2/AFF4/MLLT1/CDK9/FURIN/PDCD6IP/CPSF6/KAT2A/CHMP4B/CREBBP/EP300/VPS4B/TSG101/NELFB/CCNT2/CHMP4A/CHMP6/NELFE/HGS | 26 | Adult vs Newborn |
| bta05165 | Human papillomavirus infection | 0.242937853107345 | 0.000177260085716539 | 0.000707632119742292 | BOLA/CDKN1A/ISG15/CCND3/HES1/LFNG/MX1/LAMA3/CCND1/COL4A5/THBS1/CCND2/WNT11/ATP6V1B1/WNT7B/LAMC1/TNFRSF1A/PIK3R2/EIF2AK2/ITGB1/PTGER4/ATP6V1G1/CDC42/PKM/HDAC1/JAG1/KRAS/MDM2/IRF9/PARD6B/JAK1/TUBG1/ATP6V1F/RB1/CDK2/BCAP31/LAMB3/PARD3/PRKCI/HRAS/ITGB4/PTK2/CASP3/PPP2CA/AXIN1/GRB2/NRAS/IRF1/WNT6/HDAC2/CHD4/STAT1/APC/ATP6V0D1/ITGA2/VEGFA/PPP2R5E/CDK4/ITGB5/PPP2R3A/CREBBP/PRKCZ/PSMC1/IRF3/PXN/MAP2K2/EP300/EIF4EBP1/ITGA3/WNT10A/GSK3B/MAP2K1/ATP6V1H/LLGL1/TP53/PPP2CB/PPP2R2A/NFX1/RHEB/PATJ/PPP2R5C/RPS6KB1/ATP6V1E1/RELA/NFKB1/FADD | 86 | Adult vs Newborn |
| bta05166 | Human T-cell leukemia virus 1 infection | 0.260504201680672 | 0.000190363569083661 | 0.000745868954889197 | BOLA/IL1R2/CDKN1A/CCND3/NFKBIA/ETS2/ANAPC11/CCND1/MYC/CCND2/VDAC1/TNFRSF1A/PIK3R2/TSPO/FOS/B2M/RAN/CALR/JUN/ATF4/KRAS/JAK1/SLC2A1/XPO1/LTBR/ANAPC13/RB1/CDK2/SLC25A5/HRAS/IL1R1/NRAS/SLC25A4/CANX/VAC14/PPP3R1/RANBP1/SMAD2/KAT2A/TLN1/CDK4/BUB3/TRRAP/CREBBP/MAP3K3/MAP2K2/EP300/SMAD4/ANAPC2/BCL2L1/MAP2K1/TP53/CDC16/FDPS/ANAPC4/NFYB/GPS2/CDC27/KAT5/RELA/NFKB1/EGR1 | 62 | Adult vs Newborn |
| bta05212 | Pancreatic cancer | 0.337662337662338 | 0.000223146806655548 | 0.000858421208378279 | GADD45G/CDKN1A/GADD45B/CCND1/PIK3R2/CDC42/KRAS/JAK1/RB1/RAC1/RAC2/RALB/ERBB2/STAT1/SMAD2/ARAF/VEGFA/CDK4/RALBP1/SMAD4/BCL2L1/MAP2K1/TP53/RPS6KB1/RELA/NFKB1 | 26 | Adult vs Newborn |
| bta00480 | Glutathione metabolism | 0.353846153846154 | 0.000232028359065724 | 0.00087664849947764 | GSS/MGST1/GSTA1/GSTK1/GSTM3/IDH1/MGST3/ODC1/IDH2/GPX8/PRDX6/GSTO1/GPX1/LAP3/GGT6/GCLM/MGST2/GSTT1/RRM1/PGD/TXNDC12/LANCL1/GSTP1 | 23 | Adult vs Newborn |
| bta05216 | Thyroid cancer | 0.404761904761905 | 0.000239954749142643 | 0.000880382028178236 | PPARG/GADD45G/CDKN1A/GADD45B/CCND1/MYC/KRAS/CDH1/TFG/RXRA/HRAS/NRAS/TPM3/MAP2K2/MAP2K1/TP53/RXRB | 17 | Adult vs Newborn |
| bta03008 | Ribosome biogenesis in eukaryotes | 0.3 | 0.000241338555983188 | 0.000880382028178236 | NOP56/RAN/XPO1/TCOF1/REXO2/MPHOSPH10/XRN2/POP7/NOP58/GNL3/NXT1/GNL3L/NOB1/UTP14A/DKC1/FBL/NHP2/WDR75/XRN1/BMS1/RIOK2/UTP18/WDR43/SBDS/HEATR1/GTPBP4/NVL/CSNK2A2/LSG1/GNL2/NMD3/RIOK1/RPP25L/TBL3/NAT10/UTP6 | 36 | Adult vs Newborn |
| bta05170 | Human immunodeficiency virus 1 infection | 0.25609756097561 | 0.000282595681355663 | 0.00100572427786342 | BOLA/NFKBIA/ITPR2/GNAI2/TNFRSF1A/PAK1/PIK3R2/FOS/CALM2/PDIA3/B2M/CALR/CFL1/GNG5/CALM1/PAK4/GNB5/JUN/KRAS/GNB2/CYCS/APOBEC3H/RNF7/CUL1/RAC1/RAC2/HRAS/MAP2K6/PTK2/CASP3/MAPK13/DDB1/NRAS/PPP3R1/MAP2K3/GNAI1/GNB1/RIPK1/BID/GNA11/MYD88/IRF3/PXN/CRKL/AP1M2/CUL4B/MAP2K2/GNG12/MAP2K7/TAPBP/BCL2L1/MAP2K1/GNAI3/IRAK4/AP1B1/AP1M1/RPS6KB1/RELA/CUL5/CRK/NFKB1/LIMK2/FADD | 63 | Adult vs Newborn |
| bta00920 | Sulfur metabolism | 0.7 | 0.000285205392229924 | 0.00100572427786342 | TST/ETHE1/SUOX/SQOR/MPST/SELENBP1/BPNT1 | 7 | Adult vs Newborn |
| bta04670 | Leukocyte transendothelial migration | 0.296610169491525 | 0.000371530372610103 | 0.00128865582216792 | CLDN1/CLDN4/AFDN/GNAI2/PIK3R2/CLDN3/ITGB1/MYL12A/ACTG1/EZR/ACTB/VASP/CDC42/RASSF5/ACTN4/ARHGAP5/RAC1/MYL12B/RAC2/PTK2/MAPK13/RAP1B/ROCK2/RAPGEF3/CYBA/GNAI1/MYL9/PXN/VCL/GNAI3/PTPN11/BCAR1/RAP1A/ARHGAP35/ROCK1 | 35 | Adult vs Newborn |
| bta05160 | Hepatitis C | 0.269230769230769 | 0.000382937422503827 | 0.00130679833486026 | CLDN1/OAS1Y/CDKN1A/CLDN4/NFKBIA/MX1/CCND1/MYC/TNFRSF1A/PIK3R2/CLDN3/YWHAQ/EIF2AK2/CD81/IRF7/KRAS/IRF9/JAK1/YWHAH/YWHAZ/CYCS/RB1/CDK2/YWHAB/RXRA/HRAS/CASP3/PPP2CA/GRB2/NRAS/YWHAG/STAT1/RIPK1/ARAF/BID/CDK4/IRF3/MAP2K2/PSME3/GSK3B/MAP2K1/EIF2S1/TP53/PPP2CB/PPP2R2A/EIF2AK4/RELA/NFKB1/FADD | 49 | Adult vs Newborn |
| bta04218 | Cellular senescence | 0.274390243902439 | 0.000416137202221007 | 0.00139755351121842 | IGFBP3/BOLA/GADD45G/CDKN1A/CCND3/GADD45B/CCND1/MYC/CCND2/ITPR2/VDAC1/PIK3R2/PPP1CC/CALM2/CALM1/RASSF5/KRAS/MDM2/RB1/CDK2/SLC25A5/HRAS/MAP2K6/MAPK13/RAD50/NRAS/SLC25A4/PPP3R1/SIRT1/MAP2K3/E2F4/SMAD2/CDK4/RBBP4/MAP2K2/EIF4EBP1/NBN/MAP2K1/TRPM7/TP53/PPID/RHEB/RRAS/RELA/NFKB1 | 45 | Adult vs Newborn |
| bta04722 | Neurotrophin signaling pathway | 0.288 | 0.00057296046025833 | 0.00189416204789349 | NFKBIA/NTRK2/NTF3/BEX3/PIK3R2/CALM2/CALM1/ABL1/CAMK2D/CDC42/JUN/ATF4/KRAS/ARHGDIA/MAGED1/RAC1/HRAS/MAPK13/GRB2/NRAS/RAP1B/CRKL/MAP3K3/MAP2K2/KIDINS220/GSK3B/MAP2K7/MAP2K1/TP53/PTPN11/IRAK4/RAP1A/RELA/SHC1/CRK/NFKB1 | 36 | Adult vs Newborn |
| bta00071 | Fatty acid degradation | 0.377777777777778 | 0.00062658749701173 | 0.00203958035464547 | ACADS/ALDH2/ACAT1/ADH5/HADHB/ACADSB/ACAA2/ECHS1/HADH/ACSL3/ALDH9A1/GCDH/ACADVL/ACAT2/CPT1A/CPT2/ACADM | 17 | Adult vs Newborn |
| bta00010 | Glycolysis / Gluconeogenesis | 0.338461538461538 | 0.000638603759192497 | 0.00204719865387069 | ALDH2/PGM1/ADH5/DLD/ACSS2/LDHB/LDHA/GAPDH/PKM/GALM/FBP2/AKR1A1/PFKL/TPI1/ALDH9A1/PDHA1/PDHB/PCK2/PGM2/HK1/DLAT/GPI | 22 | Adult vs Newborn |
| bta00270 | Cysteine and methionine metabolism | 0.351851851851852 | 0.000857878977758367 | 0.00270909150871063 | AHCYL2/CDO1/GSS/TST/CBS/PSAT1/LDHB/DNMT3A/MPST/LDHA/MDH2/AHCY/DNMT1/GCLM/APIP/AHCYL1/ENOPH1/MRI1/AMD1 | 19 | Adult vs Newborn |
| bta05017 | Spinocerebellar ataxia | 0.271523178807947 | 0.000922316787020707 | 0.00286974727850096 | ITPR2/VDAC1/PIK3R2/NOP56/PSMA7/PSMD7/PSMC3/ATXN2/PUM1/ATXN10/PSMD12/ULK1/CYCS/PSMC5/SLC25A5/PSMB2/PPIF/PSMD8/SLC25A4/AFG3L2/PSMD11/CIC/PSMB5/ATXN2L/PIK3R4/PSMC1/PSMB7/PSMA2/ADRM1/PSMD6/WIPI2/PSMD1/PSMA5/PSMC4/PSMD2/ATP2A2/PSMA3/PSMD3/PSMD14/RB1CC1/KAT5 | 41 | Adult vs Newborn |
| bta04146 | Peroxisome | 0.30952380952381 | 0.00101101035276552 | 0.00310012327850297 | HMGCL/PHYH/MVK/GSTK1/CAT/IDH1/EPHX2/ECH1/PRDX5/PEX11G/XDH/AMACR/IDH2/CRAT/ACSL3/PRDX1/SOD2/MLYCD/CROT/NUDT12/PEX6/PEX19/GNPAT/AGPS/PEX2/PAOX | 26 | Adult vs Newborn |
| bta00900 | Terpenoid backbone biosynthesis | 0.476190476190476 | 0.00105011592577326 | 0.00317403460271317 | HMGCS2/ACAT1/PCYOX1/MVK/IDI1/HMGCS1/GGPS1/ACAT2/HMGCR/FDPS | 10 | Adult vs Newborn |
| bta04666 | Fc gamma R-mediated phagocytosis | 0.295918367346939 | 0.00118706073220755 | 0.0035374233828572 | MARCKSL1/ARPC5L/PLPP3/ARPC5/PAK1/PIK3R2/ARPC3/CFL1/VASP/CDC42/BIN1/SPHK2/RAC1/RAC2/MYO10/ARPC1A/MARCKS/PLPP2/ARPC1B/ACTR3/WASF2/CRKL/ACTR2/MAP2K1/DOCK1/RPS6KB1/CRK/LIMK2/DNM2 | 29 | Adult vs Newborn |
| bta05213 | Endometrial cancer | 0.333333333333333 | 0.00137305986475368 | 0.00403486890081127 | GADD45G/CDKN1A/GADD45B/CCND1/MYC/PIK3R2/KRAS/CDH1/HRAS/AXIN1/GRB2/NRAS/ERBB2/APC/ARAF/MAP2K2/GSK3B/MAP2K1/TP53/ILK | 20 | Adult vs Newborn |
| bta05222 | Small cell lung cancer | 0.292929292929293 | 0.00141379418558249 | 0.00409765870659091 | GADD45G/CDKN1A/NFKBIA/GADD45B/LAMA3/CCND1/COL4A5/MYC/LAMC1/PIK3R2/ITGB1/CYCS/RB1/CDK2/LAMB3/RXRA/PTK2/CASP3/BIRC2/TRAF4/CKS2/ITGA2/CDK4/ITGA3/BCL2L1/TP53/RELA/NFKB1/RXRB | 29 | Adult vs Newborn |
| bta04012 | ErbB signaling pathway | 0.302325581395349 | 0.00148239234393373 | 0.00423841907725006 | CDKN1A/MYC/PAK1/PIK3R2/PAK4/ABL1/CAMK2D/NRG1/JUN/KRAS/HRAS/PTK2/GRB2/NRAS/ERBB2/NCK1/ARAF/CRKL/MAP2K2/EIF4EBP1/GSK3B/MAP2K7/MAP2K1/RPS6KB1/SHC1/CRK | 26 | Adult vs Newborn |
| bta01524 | Platinum drug resistance | 0.304878048780488 | 0.00158983270511184 | 0.00448500173652603 | MGST1/CDKN1A/GSTA1/GSTM3/MGST3/REV3L/PIK3R2/GSTO1/MDM2/CYCS/CASP3/MGST2/BIRC2/ERBB2/GSTT1/BID/MSH2/SLC31A1/BCL2L1/MSH6/TP53/MSH3/TOP2B/GSTP1/FADD | 25 | Adult vs Newborn |
| bta03020 | RNA polymerase | 0.393939393939394 | 0.00172150528499333 | 0.00479255626431661 | POLR2F/CRCP/POLR2K/POLR3K/POLR1D/POLR2J/POLR1C/POLR2D/POLR3D/POLR2E/POLR2A/POLR2C/POLR2B | 13 | Adult vs Newborn |
| bta01230 | Biosynthesis of amino acids | 0.310810810810811 | 0.00181391808751567 | 0.00498424518920915 | SHMT1/SHMT2/PSPH/IDH3B/IDH1/CBS/TKT/IDH3G/PSAT1/IDH2/GLUL/GAPDH/PKM/PFKL/TPI1/ACO2/PYCR3/ASS1/PRPS1/PYCR2/PYCR1/RPE/IDH3A | 23 | Adult vs Newborn |
| bta03083 | Polycomb repressive complex | 0.301204819277108 | 0.00191796905173605 | 0.00520258811604516 | AUTS2/YY1/RING1/HDAC1/CBX4/SUZ12/PHC2/TEX10/HDAC2/UBE2D3/RBBP7/RYBP/TFDP1/KDM2A/MTF2/MGA/RBBP4/WDR5/CREBBP/EP300/AEBP2/UBE2D2/DCAF7/ASXL1/EED | 25 | Adult vs Newborn |
| bta04360 | Axon guidance | 0.25414364640884 | 0.00208877197903707 | 0.00559417945085211 | NGEF/GNAI2/SEMA3F/PAK1/PIK3R2/EFNA5/ITGB1/CFL1/MYL12A/PAK4/ABL1/CAMK2D/CDC42/KRAS/PARD6B/SSH3/PARD3/RAC1/MYL12B/RAC2/HRAS/SEMA4A/ENAH/PTK2/EFNA1/NRAS/EPHB3/PPP3R1/ROCK2/NCK1/GNAI1/CDK5/MYL9/BOC/PRKCZ/PLXNB2/GSK3B/GNAI3/EPHA1/PTPN11/RRAS/ROCK1/BMPR2/RND1/ILK/LIMK2 | 46 | Adult vs Newborn |
| bta04010 | MAPK signaling pathway | 0.233333333333333 | 0.00214485912527053 | 0.00567258794972865 | IGF2/GADD45G/STMN1/PDGFA/GADD45B/NTRK2/NTF3/MYC/DUSP1/TNFRSF1A/PAK1/EFNA5/JUND/FOS/HSPA8/HSPB1/DDIT3/CDC42/JUN/NR4A1/DUSP7/ATF4/KRAS/CSF1/VEGFB/RAC1/MECOM/MKNK2/RAC2/HRAS/CD14/MAP2K6/CASP3/MAPK13/EFNA1/IL1R1/GRB2/NRAS/RAP1B/PPP3R1/MAP2K3/ERBB2/PPM1A/ARAF/FGFR2/RPS6KA4/VEGFA/MAP3K20/TAOK3/MYD88/CRKL/MAP3K3/MAP2K2/GNG12/DAXX/MAPKAPK3/MAP2K7/MAP2K1/DUSP6/TP53/FLNA/IRAK4/RAP1A/LAMTOR3/PPM1B/RRAS/PPP5C/RELA/CRK/NFKB1 | 70 | Adult vs Newborn |
| bta05167 | Kaposi sarcoma-associated herpesvirus infection | 0.244343891402715 | 0.0023334667974765 | 0.00595209773139158 | BOLA/RCAN1/CDKN1A/NFKBIA/CCND1/MYC/ITPR2/TNFRSF1A/PIK3R2/EIF2AK2/FOS/CALM2/GNG5/RPS27A/CALM1/UBB/GNB5/JUN/IRF7/KRAS/GNB2/IRF9/JAK1/MAP1LC3A/CYCS/RB1/RAC1/HRAS/MAP2K6/CASP3/MAPK13/NRAS/PPP3R1/STAT1/GNB1/JAK2/VEGFA/BID/CDK4/CREBBP/IRF3/ATG3/MAP2K2/EP300/GNG12/GSK3B/MAP2K7/MAP2K1/TP53/MAP1LC3B/IFNGR1/RELA/NFKB1/FADD | 54 | Adult vs Newborn |
| bta04071 | Sphingolipid signaling pathway | 0.272 | 0.00234392575176089 | 0.00595209773139158 | ASAH1/GNAI2/TNFRSF1A/PIK3R2/DEGS1/KRAS/S1PR5/SGMS1/SPHK2/RAC1/RAC2/HRAS/SMPD2/MAPK13/PPP2CA/NRAS/ROCK2/GNAI1/BID/PPP2R5E/PPP2R3A/PRKCZ/MAP2K2/CERS2/SPTLC2/MAP2K1/TP53/GNAI3/PPP2CB/PPP2R2A/ROCK1/PPP2R5C/RELA/NFKB1 | 34 | Adult vs Newborn |
| bta04390 | Hippo signaling pathway | 0.259493670886076 | 0.00235343246170998 | 0.00595209773139158 | ID2/CCND3/CCN2/CCND1/MYC/CCND2/WNT11/WNT7B/PPP1CC/YWHAQ/ACTG1/ACTB/CDH1/PARD6B/YWHAH/YWHAZ/PARD3/YWHAB/PRKCI/MOB1B/BMPR1A/PPP2CA/AXIN1/BIRC2/WNT6/YWHAG/APC/SMAD2/LATS1/FRMD6/PRKCZ/WNT10A/GSK3B/SMAD4/LLGL1/PPP2CB/PPP2R2A/PATJ/CSNK1D/RASSF1/BMPR2 | 41 | Adult vs Newborn |
| bta01210 | 2-Oxocarboxylic acid metabolism | 0.382352941176471 | 0.00236307163664203 | 0.00595209773139158 | IDH3B/BCKDHA/IDH1/IDH3G/DLD/IDH2/ACO2/PDHA1/PDHB/OGDH/BCKDHB/DLAT/IDH3A | 13 | Adult vs Newborn |
| bta00330 | Arginine and proline metabolism | 0.333333333333333 | 0.00306329807234804 | 0.0076250515485072 | MAOA/PRODH/ALDH2/CKMT1A/P4HA2/ODC1/SAT1/AZIN2/LAP3/ALDH9A1/CNDP2/PYCR3/GATM/AMD1/CKB/PYCR2/PYCR1 | 17 | Adult vs Newborn |
| bta04066 | HIF-1 signaling pathway | 0.271929824561404 | 0.00357950209365305 | 0.00880636377997875 | CDKN1A/LDHB/PIK3R2/EGLN3/LDHA/GAPDH/CAMK2D/PDK1/PFKL/SLC2A1/LTBR/PDHA1/MKNK2/EIF4E2/EIF4E/PDHB/EGLN1/ERBB2/HK1/VEGFA/CREBBP/MAP2K2/EP300/EIF4EBP1/EGLN2/CUL2/MAP2K1/IFNGR1/RPS6KB1/RELA/NFKB1 | 31 | Adult vs Newborn |
| bta04015 | Rap1 signaling pathway | 0.240740740740741 | 0.0038591565618408 | 0.00938524463315187 | PDGFA/AFDN/THBS1/GNAI2/PIK3R2/EFNA5/CALM2/ITGB1/ACTG1/ACTB/CALM1/PFN1/VASP/CDC42/EVL/PRKD2/RASSF5/KRAS/CSF1/CDH1/PARD6B/SIPA1L3/VEGFB/PARD3/RAC1/RAC2/PRKCI/HRAS/MAP2K6/ENAH/MAPK13/EFNA1/NRAS/RAP1B/SIPA1L1/RALB/MAP2K3/RAPGEF3/GNAI1/FGFR2/VEGFA/TLN1/PRKCZ/CRKL/MAP2K2/MAP2K1/GNAI3/BCAR1/RAP1A/SIPA1L2/RRAS/CRK | 52 | Adult vs Newborn |
| bta05223 | Non-small cell lung cancer | 0.297297297297297 | 0.00415880342209288 | 0.00999903693589316 | GADD45G/CDKN1A/GADD45B/CCND1/KIF5B/PIK3R2/RASSF5/KRAS/RB1/RXRA/HRAS/EML4/GRB2/NRAS/ERBB2/ARAF/CDK4/MAP2K2/MAP2K1/TP53/RASSF1/RXRB | 22 | Adult vs Newborn |
| bta05417 | Lipid and atherosclerosis | 0.235537190082645 | 0.0042627145158169 | 0.0101337151706588 | PPARG/NFE2L2/NFKBIA/LY96/HSPA5/TNFRSF1A/PIK3R2/FOS/CALM2/HSP90B1/HSPA8/CALM1/HSP90AA1/HSP90AB1/DDIT3/CAMK2D/CDC42/JUN/IRF7/ATF4/KRAS/PYCARD/CYCS/SOD2/RAC1/RXRA/HSPA4/HRAS/CD14/MAP2K6/PTK2/CASP3/MAPK13/NRAS/IL18/RAP1B/PPP3R1/MAP2K3/ROCK2/CYBA/HSPD1/JAK2/BID/CASP6/MYD88/IRF3/ARHGEF1/GSK3B/MAP2K7/BCL2L1/EIF2S1/TP53/IRAK4/RAP1A/RELA/NFKB1/RXRB | 57 | Adult vs Newborn |
| bta04115 | p53 signaling pathway | 0.284090909090909 | 0.00455946969285549 | 0.010643176650341 | IGFBP3/GADD45G/CDKN1A/CCND3/GADD45B/CCND1/THBS1/CCND2/AIFM2/SIVA1/MDM2/CYCS/CDK2/ZNF385A/EI24/CASP3/SESN1/BID/CDK4/CCNG1/SIAH1/BCL2L1/TP53/SFN/RCHY1 | 25 | Adult vs Newborn |
| bta00983 | Drug metabolism - other enzymes | 0.291139240506329 | 0.0045776249821989 | 0.010643176650341 | UGT1A1/MGST1/NAT1/GSTA1/GSTM3/MGST3/IMPDH1/XDH/CDA/DUT/GSTO1/NME2/IMPDH2/CMPK1/MGST2/GSTT1/RRM1/HPRT1/NME7/GMPS/NME1/GSTP1/UCK1 | 23 | Adult vs Newborn |
| bta00310 | Lysine degradation | 0.303030303030303 | 0.00483527261734942 | 0.0111200205501972 | ALDH2/ACAT1/DLD/ECHS1/HADH/ALDH9A1/GCDH/MECOM/PRDM2/ACAT2/COLGALT1/KMT2C/PLOD1/KMT2E/NSD1/KMT5A/KMT5B/SETD2/EHMT2/KMT2A | 20 | Adult vs Newborn |
| bta05215 | Prostate cancer | 0.271028037383178 | 0.00498768913399807 | 0.0113472044814218 | IL1R2/CDKN1A/PDGFA/NFKBIA/CCND1/PIK3R2/HSP90B1/HSP90AA1/HSP90AB1/ATF4/KRAS/MDM2/RB1/CDK2/HRAS/GRB2/NRAS/ERBB2/ARAF/FGFR2/CREBBP/MAP2K2/EP300/GSK3B/MAP2K1/TP53/GSTP1/RELA/NFKB1 | 29 | Adult vs Newborn |
| bta01240 | Biosynthesis of cofactors | 0.251612903225806 | 0.00524289191617652 | 0.0118009101360748 | ALDH2/AK1/UGT1A1/SHMT1/SHMT2/GSS/ALAS1/COQ2/DLD/PSAT1/RFK/AK2/NME2/NQO1/FPGS/ALAD/AKR1A1/COQ5/DHFR/NFS1/GCLM/CMPK1/PPCDC/UGP2/UGDH/COQ4/VKORC1/COQ7/ADSS1/PDXK/UROD/LIAS/ADSS2/EPRS1/AK3/MOCS2/ADSL/NME7/NME1 | 39 | Adult vs Newborn |
| bta00030 | Pentose phosphate pathway | 0.379310344827586 | 0.0054108803705295 | 0.0120508249803482 | GLYCTK/PGM1/TKT/FBP2/PFKL/PGLS/PGM2/PRPS1/GPI/RPE/PGD | 11 | Adult vs Newborn |
| bta04136 | Autophagy - other | 0.363636363636364 | 0.00554218739387687 | 0.0122146893220313 | GABARAP/ATG7/GABARAPL1/ATG4D/PPP2CA/PIK3R4/ATG3/WIPI2/ATG4B/ATG16L1/PPP2CB/ATG5 | 12 | Adult vs Newborn |
| bta05235 | PD-L1 expression and PD-1 checkpoint pathway in cancer | 0.273684210526316 | 0.00661837023995991 | 0.0144361629759299 | NFKBIA/PIK3R2/FOS/JUN/KRAS/JAK1/PTPN6/HRAS/MAP2K6/MAPK13/EML4/NRAS/PPP3R1/MAP2K3/STAT1/JAK2/CSNK2A2/MYD88/MAP3K3/MAP2K2/MAP2K1/PTPN11/IFNGR1/RPS6KB1/RELA/NFKB1 | 26 | Adult vs Newborn |
| bta00240 | Pyrimidine metabolism | 0.305084745762712 | 0.00676552700142916 | 0.014459021023788 | NT5E/NT5C/ENTPD3/CDA/DUT/NME2/NT5C3B/CMPK1/DCTPP1/DTYMK/TYMS/RRM1/NT5C2/NME7/NME1/HDDC2/NUDT2/UCK1 | 18 | Adult vs Newborn |
| bta01212 | Fatty acid metabolism | 0.305084745762712 | 0.00676552700142916 | 0.014459021023788 | ACADS/ACAT1/CBR4/HADHB/ACADSB/ACAA2/ECHS1/HADH/ACSL3/ELOVL6/ACADVL/ACAT2/FASN/CPT1A/CPT2/ACADM/MECR/HACD2 | 18 | Adult vs Newborn |
| bta01521 | EGFR tyrosine kinase inhibitor resistance | 0.280487804878049 | 0.00750351976126768 | 0.0158758681264716 | PDGFA/PIK3R2/GAS6/NRG1/KRAS/JAK1/HRAS/EIF4E2/GRB2/NRAS/EIF4E/ERBB2/JAK2/ARAF/FGFR2/VEGFA/MAP2K2/EIF4EBP1/GSK3B/BCL2L1/MAP2K1/RPS6KB1/SHC1 | 23 | Adult vs Newborn |
| bta00513 | Various types of N-glycan biosynthesis | 0.319148936170213 | 0.00823609300057566 | 0.0169727294666753 | OSTC/DDOST/ALG14/KRTCAP2/RPN2/MGAT4B/MGAT1/HEXB/STT3A/B4GALT1/STT3B/TMEM258/MAN1A1/B4GALT2/MAN1A2 | 15 | Adult vs Newborn |
| bta05221 | Acute myeloid leukemia | 0.289855072463768 | 0.00830278126849425 | 0.0169727294666753 | ZBTB16/CCND1/MYC/PIK3R2/KRAS/PPARD/HRAS/CD14/GRB2/NRAS/PIM1/ARAF/MAP2K2/EIF4EBP1/MAP2K1/DUSP6/JUP/RPS6KB1/RELA/NFKB1 | 20 | Adult vs Newborn |
| bta04148 | Efferocytosis | 0.242603550295858 | 0.0083280869961089 | 0.0169727294666753 | PPARG/ADAM10/SLC16A1/THBS1/DNMT3A/ODC1/CALR/PTGER4/GAS6/CAMK2D/UQCRFS1/DUSP7/PPARD/MFGE8/SLC2A1/SIRPA/SPHK2/RAC1/RXRA/PTPN6/SGK1/RAB5A/LIPA/PTK2/CASP3/MAPK13/CPT1A/SIRT1/JAK2/CX3CL1/ITGB5/CRKL/MAP2K2/BSG/RAB7A/ATP2A2/MAP2K1/DOCK1/PTPN11/BCAR1/CRK | 41 | Adult vs Newborn |
| bta00130 | Ubiquinone and other terpenoid-quinone biosynthesis | 0.5 | 0.00834281428511203 | 0.0169727294666753 | COQ2/NQO1/COQ5/COQ4/VKORC1/COQ7 | 6 | Adult vs Newborn |
| bta04150 | mTOR signaling pathway | 0.242424242424242 | 0.00919252842438378 | 0.0185232903589087 | CASTOR1/DDIT4/WNT11/ATP6V1B1/WNT7B/TNFRSF1A/PIK3R2/CAB39L/EIF4B/ATP6V1G1/KRAS/ULK1/ATP6V1F/RRAGB/LRP5/SGK1/HRAS/EIF4E2/GRB2/NRAS/EIF4E/RRAGA/WNT6/SLC7A5/RRAGC/SEC13/CAB39/MAP2K2/EIF4EBP1/WNT10A/GSK3B/MAP2K1/ATP6V1H/NPRL2/SEH1L/MAPKAP1/LAMTOR3/RHEB/RPS6KB1/ATP6V1E1 | 40 | Adult vs Newborn |
| bta00510 | N-Glycan biosynthesis | 0.298245614035088 | 0.0106448420125019 | 0.0212474006406443 | OSTC/DDOST/DPM3/ALG14/KRTCAP2/RPN2/MGAT4B/MGAT1/ALG5/STT3A/B4GALT1/STT3B/TMEM258/MAN1A1/B4GALT2/MAN1A2/DPM2 | 17 | Adult vs Newborn |
| bta03410 | Base excision repair | 0.318181818181818 | 0.0108177657792843 | 0.0213907616491506 | HMGB1/PCNA/PARP1/APEX1/POLE3/LIG1/POLD3/RFC5/TDG/RFC1/RFC2/XRCC1/POLD2/RFC3 | 14 | Adult vs Newborn |
| bta00230 | Purine metabolism | 0.24812030075188 | 0.011970034852855 | 0.023450068278985 | AK1/PGM1/NT5E/NT5C/ADK/IMPDH1/ENTPD3/XDH/AK2/NME2/APRT/HDDC3/IMPDH2/NUDT16/PGM2/ADSS1/PRPS1/ATIC/ADSS2/RRM1/NT5C2/AK3/PPAT/DGUOK/GART/HPRT1/ADSL/NME7/GMPS/NME1/HDDC2/NUDT2/GMPR2 | 33 | Adult vs Newborn |
| bta04211 | Longevity regulating pathway | 0.266666666666667 | 0.0123397397047005 | 0.0239525608946867 | PPARG/CAT/PIK3R2/ATF4/KRAS/ULK1/SOD2/HRAS/EIF4E2/NRAS/EIF4E/SIRT1/SESN1/EIF4EBP1/ADIPOR1/EHMT2/TP53/ATG5/APPL1/RHEB/RPS6KB1/RB1CC1/RELA/NFKB1 | 24 | Adult vs Newborn |
| bta00785 | Lipoic acid metabolism | 0.375 | 0.0125383285846239 | 0.0241167851244919 | BCKDHA/DLD/GCSH/PDHA1/PDHB/OGDH/BCKDHB/DLAT/LIAS | 9 | Adult vs Newborn |
| bta05134 | Legionellosis | 0.293103448275862 | 0.0127541691127375 | 0.0243109340128994 | NFKBIA/HSPA8/ARF1/EEF1A1/PYCARD/CYCS/CD14/BCL2L13/CASP3/IL18/HSPD1/ARF2/CLK1/MYD88/CLK4/RELA/NFKB1 | 17 | Adult vs Newborn |
| bta05203 | Viral carcinogenesis | 0.224 | 0.0132066571035705 | 0.0249486661449029 | BOLA/CDKN1A/CCND3/NFKBIA/CCND1/CCND2/PIK3R2/YWHAQ/EIF2AK2/CDC42/JUN/PKM/HDAC1/IRF7/ATF4/KRAS/ACTN4/MDM2/IRF9/JAK1/YWHAH/LTBR/YWHAZ/RB1/CDK2/RAC1/YWHAB/HRAS/CASP3/DDB1/GRB2/NRAS/VAC14/RANBP1/HDAC2/CHD4/YWHAG/ATP6V0D1/KAT2A/GTF2A2/CDK4/GTF2A1/CREBBP/PSMC1/IRF3/PXN/EP300/SND1/GTF2E2/TP53/SNW1/HDAC7/GTF2H1/RELA/HDAC3/NFKB1 | 56 | Adult vs Newborn |
| bta04260 | Cardiac muscle contraction | 0.257142857142857 | 0.0136042913996325 | 0.0254037342964107 | ATP1B1/UQCRC2/ATP1A1/TPM1/UQCRC1/CYC1/UQCR10/COX7A2L/COX6A1/COX5B/UQCRH/COX4I1/COX1/COX2/COX3/CYTB/COX7A1/UQCRFS1/COX7B/UQCR11/COX6B1/SLC9A1/UQCRB/UQCRQ/TPM3/ATP2A2/COX8B | 27 | Adult vs Newborn |
| bta04310 | Wnt signaling pathway | 0.23463687150838 | 0.0136876837029914 | 0.0254037342964107 | SFRP1/CTNNBIP1/CCND3/DAAM1/CCND1/MYC/CCND2/WNT11/WNT7B/CACYBP/CAMK2D/JUN/PPARD/CUL1/RAC1/LRP5/RAC2/TBL1XR1/TLE4/AXIN1/CTBP2/PPP3R1/SIRT1/WNT6/LGR4/ROCK2/APC/CHD8/ZNRF3/CTBP1/CSNK2A2/CREBBP/INVS/EP300/WNT10A/SIAH1/GSK3B/SMAD4/RUVBL1/TP53/CCAR2/PRICKLE3 | 42 | Adult vs Newborn |
| bta04370 | VEGF signaling pathway | 0.288135593220339 | 0.0151781433006481 | 0.0279250050657232 | PIK3R2/HSPB1/CDC42/KRAS/SPHK2/RAC1/RAC2/HRAS/PTK2/MAPK13/NRAS/PPP3R1/VEGFA/PXN/MAP2K2/MAPKAPK3/MAP2K1 | 17 | Adult vs Newborn |
| bta04122 | Sulfur relay system | 0.5 | 0.0161554748049792 | 0.0294336857057822 | TST/MPST/NFS1/CTU2/MOCS2 | 5 | Adult vs Newborn |
| bta04152 | AMPK signaling pathway | 0.246031746031746 | 0.0163699389986274 | 0.0294336857057822 | PPARG/CCND1/TBC1D1/CIDEA/PIK3R2/CAB39L/FBP2/PFKL/ULK1/RAB2A/MLYCD/FASN/PPP2CA/RAB11B/ELAVL1/CPT1A/SIRT1/PCK2/PPP2R5E/PPP2R3A/HMGCR/CAB39/EIF4EBP1/RAB10/ADIPOR1/PPP2CB/PPP2R2A/RHEB/RAB8A/PPP2R5C/RPS6KB1 | 31 | Adult vs Newborn |
| bta05133 | Pertussis | 0.269230769230769 | 0.0165850116316334 | 0.0294336857057822 | LY96/GNAI2/FOS/CALM2/ITGB1/CFL1/CALM1/JUN/PYCARD/CD14/CASP3/MAPK13/IRF1/GNAI1/MYD88/IRF3/C1R/GNAI3/IRAK4/RELA/NFKB1 | 21 | Adult vs Newborn |
| bta05214 | Glioma | 0.269230769230769 | 0.0165850116316334 | 0.0294336857057822 | GADD45G/CDKN1A/PDGFA/GADD45B/CCND1/PIK3R2/CALM2/CALM1/CAMK2D/KRAS/MDM2/RB1/HRAS/GRB2/NRAS/ARAF/CDK4/MAP2K2/MAP2K1/TP53/SHC1 | 21 | Adult vs Newborn |
| bta04142 | Lysosome | 0.24113475177305 | 0.0166937321913392 | 0.0294336857057822 | LAPTM4B/CTSC/ACP5/CTSH/ASAH1/CLTB/CD63/HYAL2/PSAP/CTSF/LAPTM4A/GGA2/CLTC/DNASE2/MAN2B1/LIPA/NPC2/FUCA1/LGMN/ATP6V0D1/GNPTG/HEXB/M6PR/AP1M2/SCARB2/CTSV/CTSB/ATP6V1H/AP3D1/AP3B1/CD164/AP1B1/AP1M1/AP4M1 | 34 | Adult vs Newborn |
| bta04151 | PI3K-Akt signaling pathway | 0.209183673469388 | 0.0188332483153013 | 0.0329315607775169 | IGF2/CDKN1A/PDGFA/CCND3/NTRK2/NTF3/PRLR/LAMA3/CCND1/DDIT4/COL4A5/THBS1/MYC/CCND2/LAMC1/PIK3R2/EFNA5/YWHAQ/HSP90B1/EIF4B/ITGB1/GNG5/HSP90AA1/HSP90AB1/GNB5/NR4A1/ATF4/KRAS/CSF1/MDM2/GNB2/MCL1/SGK3/JAK1/YWHAH/YWHAZ/CDK2/VEGFB/LAMB3/RAC1/YWHAB/RXRA/SGK1/HRAS/ITGB4/PTK2/EFNA1/EIF4E2/PPP2CA/GRB2/NRAS/EIF4E/PCK2/IL4R/ERBB2/YWHAG/GNB1/PKN1/JAK2/ITGA2/FGFR2/VEGFA/PPP2R5E/CDK4/ITGB5/PPP2R3A/MAP2K2/EIF4EBP1/GNG12/ITGA3/GSK3B/PKN2/BCL2L1/MAP2K1/TP53/PPP2CB/PPP2R2A/RHEB/PPP2R5C/RPS6KB1/RELA/NFKB1 | 82 | Adult vs Newborn |
| bta05161 | Hepatitis B | 0.229508196721311 | 0.0196996317897394 | 0.0341641586690045 | CDKN1A/NFKBIA/MYC/PIK3R2/YWHAQ/FOS/PCNA/JUN/IRF7/ATF4/KRAS/JAK1/YWHAZ/CYCS/RB1/CDK2/YWHAB/HRAS/MAP2K6/CASP3/MAPK13/DDB1/GRB2/NRAS/MAP2K3/STAT1/JAK2/ARAF/BID/CREBBP/MYD88/IRF3/MAP2K2/EP300/MAP2K7/SMAD4/MAP2K1/TP53/IRAK4/RELA/NFKB1/FADD | 42 | Adult vs Newborn |
| bta04723 | Retrograde endocannabinoid signaling | 0.233766233766234 | 0.0224328643381949 | 0.0385802718142997 | ND4L/NDUFS7/NDUFS2/NDUFV1/ITPR2/GNAI2/NDUFB3/NDUFAB1/ND5/GNG5/ND3/ND1/ND4/ND2/NDUFA9/NDUFB9/NDUFA1/NDUFA13/GNB5/NDUFC2/NDUFS5/GNB2/NDUFA10/NDUFS1/NDUFA8/NDUFS8/NDUFV2/NDUFB7/MAPK13/NDUFA4/NDUFS3/GNAI1/GNB1/ND6/GNG12/GNAI3 | 36 | Adult vs Newborn |
| bta04915 | Estrogen signaling pathway | 0.237410071942446 | 0.0226107264662911 | 0.0385802718142997 | KRT17/KRT42/FKBP5/KRT19/KRT14/ITPR2/GNAI2/PIK3R2/FOS/CALM2/HSP90B1/HSPA8/FKBP4/CALM1/HSP90AA1/KRT15/HSP90AB1/JUN/KRT18/ATF4/KRAS/HRAS/GRB2/NRAS/GNAI1/EBAG9/MAP2K2/NCOA1/MAP2K1/GNAI3/KRT10/POMC/SHC1 | 33 | Adult vs Newborn |
| bta01522 | Endocrine resistance | 0.252631578947368 | 0.0236394340318672 | 0.0400128525507815 | CDKN1A/CCND1/PIK3R2/FOS/JUN/JAG1/KRAS/MDM2/NCOR1/RB1/HRAS/PTK2/MAPK13/GRB2/NRAS/ERBB2/ARAF/CDK4/MAP2K2/MAP2K1/TP53/MED1/RPS6KB1/SHC1 | 24 | Adult vs Newborn |
| bta04935 | Growth hormone synthesis, secretion and action | 0.241666666666667 | 0.0248977926554376 | 0.0418083235066245 | IGFBP3/ITPR2/GNAI2/PIK3R2/JUNB/FOS/ATF4/KRAS/HRAS/MAP2K6/PTK2/MAPK13/GRB2/NRAS/MAP2K3/STAT1/GNAI1/JAK2/CREBBP/GNA11/CRKL/MAP2K2/EP300/GSK3B/MAP2K1/GNAI3/BCAR1/SHC1/CRK | 29 | Adult vs Newborn |
| bta04145 | Phagosome | 0.228915662650602 | 0.026545585502596 | 0.0442243073851787 | BOLA/TUBB4B/TUBA1B/THBS1/TUBA4A/ATP6V1B1/TUBA1A/ITGB1/CALR/ACTG1/ACTB/ATP6V1G1/ATP6V1F/RAC1/RAB5A/CD14/CANX/TUBB6/CYBA/TUBA1C/DYNC1H1/ATP6V0D1/ITGA2/DYNC1LI1/ITGB5/STX7/DYNC1LI2/M6PR/C1R/CTSV/PLA2R1/RAB7A/EEA1/ATP6V1H/TUBB/ATP6V1E1/SEC61A1/HGS | 38 | Adult vs Newborn |
| bta00650 | Butanoate metabolism | 0.333333333333333 | 0.0280008644698684 | 0.0458523862296383 | HMGCS2/BDH1/ACADS/ACAT1/HMGCL/ECHS1/HADH/HMGCS1/ACAT2 | 9 | Adult vs Newborn |
| bta04979 | Cholesterol metabolism | 0.285714285714286 | 0.0280544945275387 | 0.0458523862296383 | VDAC1/TSPO/PLTP/ANGPTL4/VAPA/MYLIP/OSBPL5/CYP27A1/LIPA/NPC2/NCEH1/LDLRAP1/VAPB/LCAT | 14 | Adult vs Newborn |
| bta04382 | Cornified envelope formation | 0.221153846153846 | 0.0283385542637799 | 0.0458523862296383 | CLDN1/KRT4/KRT17/KRT42/KRT6A/KRT7/KRT19/CLDN4/S100A9/KRT14/LAMA3/EPHX2/COL4A5/S100A13/DSG3/CLDN3/S100A16/S100A11/S100A4/S100A2/KRT15/COL17A1/KRT18/S100A14/SLC27A1/ST14/PCSK6/LAMB3/DSG2/ITGB4/PPP2CA/KRT8/BLMH/FURIN/PPP2R5E/PPP2R3A/PKP1/DST/SPTLC2/UGCG/PPP2CB/PPP2R2A/KRT10/S100A8/JUP/PPP2R5C | 46 | Adult vs Newborn |
| bta03271 | Virion - Rotavirus | 1 | 0.0283896988372064 | 0.0458523862296383 | ITGB1/ITGA2 | 2 | Adult vs Newborn |
| bta04933 | AGE-RAGE signaling pathway in diabetic complications | 0.245098039215686 | 0.0302118773780615 | 0.0484257364672277 | F3/CCND1/COL4A5/PIK3R2/CDC42/JUN/KRAS/VEGFB/RAC1/HRAS/CASP3/MAPK13/NRAS/PLCD1/STAT1/JAK2/SMAD2/PIM1/VEGFA/CDK4/PRKCZ/SMAD4/RELA/NFKB1/EGR1 | 25 | Adult vs Newborn |
| bta05145 | Toxoplasmosis | 0.239316239316239 | 0.0306063735493504 | 0.0486892052506485 | NFKBIA/LY96/LAMA3/GNAI2/LAMC1/TNFRSF1A/HSPA8/ITGB1/JAK1/CYCS/LAMB3/MAP2K6/PPIF/CASP3/MAPK13/BIRC2/MAP2K3/STAT1/GNAI1/JAK2/MYD88/BCL2L1/GNAI3/IRAK4/IFNGR1/RELA/NFKB1/IL10RB | 28 | Adult vs Newborn |
| bta05202 | Transcriptional misregulation in cancer | 0.220588235294118 | 0.0310708720155967 | 0.0490592716035737 | IGFBP3/PPARG/IL1R2/GADD45G/ZBTB16/CDKN1A/PDGFA/ID2/GADD45B/TSPAN7/MYC/AFF1/CCND2/MLLT3/FUS/DDX5/DDIT3/HDAC1/MDM2/NCOR1/HPGD/JMJD1C/RXRA/CD14/PTK2/KLF3/BIRC2/MLLT1/ASPSCR1/CDK9/HDAC2/BCL6/EWSR1/BCL2L1/DUSP6/PRCC/TP53/KMT2A/LDB1/JUP/ATF1/RELA/CCNT2/NFKB1/RXRB | 45 | Adult vs Newborn |
| bta04660 | T cell receptor signaling pathway | 0.234375 | 0.0337045873849599 | 0.0521502321334972 | NFKBIA/PAK1/PIK3R2/FOS/PAK4/CDC42/JUN/KRAS/PTPN6/HRAS/MAPK13/PPP2CA/GRB2/NRAS/PPP3R1/NCK1/BCL10/PPP2R5E/CDK4/PPP2R3A/MAP2K2/GSK3B/MAP2K7/MAP2K1/PTPN11/PPP2CB/PPP2R2A/PPP2R5C/RELA/NFKB1 | 30 | Adult vs Newborn |
| bta00520 | Amino sugar and nucleotide sugar metabolism | 0.292682926829268 | 0.0337679239411674 | 0.0521502321334972 | PGM1/UGP2/UGDH/UAP1/AMDHD2/PGM2/GNPDA2/HK1/HEXB/GNPDA1/GALK1/GPI | 12 | Adult vs Newborn |
| bta00670 | One carbon pool by folate | 0.292682926829268 | 0.0337679239411674 | 0.0521502321334972 | AHCYL2/SHMT1/SHMT2/CBS/DLD/GCSH/AHCY/DHFR/AHCYL1/TYMS/ATIC/GART | 12 | Adult vs Newborn |
| bta04720 | Long-term potentiation | 0.257142857142857 | 0.0390027299216368 | 0.0597982358066285 | ITPR2/PPP1CC/CALM2/CALM1/CAMK2D/ATF4/KRAS/HRAS/NRAS/RAP1B/PPP3R1/RAPGEF3/ARAF/CREBBP/MAP2K2/EP300/MAP2K1/RAP1A | 18 | Adult vs Newborn |
| bta04550 | Signaling pathways regulating pluripotency of stem cells | 0.227586206896552 | 0.0395812983465999 | 0.0602487010046693 | ID3/ID2/MYC/KLF4/ACVR2A/WNT11/WNT7B/PIK3R2/SMARCAD1/KRAS/JAK1/ZFHX3/HRAS/RIF1/MAPK13/BMPR1A/AXIN1/GRB2/NRAS/KAT6A/WNT6/APC/JAK2/SMAD2/FGFR2/SMAD5/LIFR/MAP2K2/WNT10A/GSK3B/SMAD4/MAP2K1/BMPR2 | 33 | Adult vs Newborn |
| bta04068 | FoxO signaling pathway | 0.22962962962963 | 0.0403609232913927 | 0.0609965833200747 | GADD45G/CDKN1A/GADD45B/CAT/CCND1/CCND2/PIK3R2/GABARAP/GABARAPL1/KRAS/MDM2/SGK3/PLK2/CDK2/SOD2/SGK1/HRAS/MAPK13/GRB2/DEPP1/NRAS/SIRT1/PCK2/PRMT1/ARAF/CREBBP/BCL6/MAP2K2/EP300/SMAD4/MAP2K1 | 31 | Adult vs Newborn |
| bta04917 | Prolactin signaling pathway | 0.247058823529412 | 0.0409699953029573 | 0.0614779324814813 | PRLR/CCND1/CCND2/ELF5/PIK3R2/FOS/KRAS/CISH/HRAS/MAPK13/GRB2/NRAS/IRF1/STAT1/JAK2/MAP2K2/GSK3B/MAP2K1/RELA/SHC1/NFKB1 | 21 | Adult vs Newborn |
| bta04330 | Notch signaling pathway | 0.262295081967213 | 0.0421734880362844 | 0.06283818454628 | HES1/LFNG/NCOR2/HDAC1/JAG1/TLE4/CTBP2/HDAC2/KAT2A/CTBP1/SPEN/CREBBP/EP300/DTX2/SNW1/APH1A | 16 | Adult vs Newborn |
| bta05164 | Influenza A | 0.217171717171717 | 0.0433110739306915 | 0.0640818981234376 | OAS1Y/CCND3/NFKBIA/MX1/VDAC1/TNFRSF1A/PIK3R2/EIF2AK2/ACTG1/ACTB/IRF7/IRF9/PYCARD/JAK1/XPO1/PABPN1/CYCS/SLC25A5/CALCOCO2/CASP3/NXT1/SLC25A4/RAB11B/IL18/STAT1/NUP98/JAK2/BID/CDK4/CREBBP/MYD88/IRF3/MAP2K2/EP300/DNAJC3/MAP2K1/EIF2S1/IRAK4/FDPS/IFNGR1/RELA/NFKB1/FADD | 43 | Adult vs Newborn |
| bta05162 | Measles | 0.222222222222222 | 0.0450296166067521 | 0.0661619366809735 | OAS1Y/CCND3/NFKBIA/MX1/CCND1/CCND2/PIK3R2/EIF2AK2/FOS/HSPA8/RACK1/JUN/IRF7/IRF9/JAK1/EIF3H/CYCS/CDK2/CASP3/STAT1/BID/CDK4/CSNK2A2/MYD88/IRF3/GSK3B/CD46/BCL2L1/EIF2S1/TP53/IRAK4/EIF2AK4/RELA/NFKB1/FADD/RCHY1 | 36 | Adult vs Newborn |
| bta00740 | Riboflavin metabolism | 0.444444444444444 | 0.0497150001302965 | 0.0725423958344073 | BLVRB/ACP5/RFK/ACP1 | 4 | Adult vs Newborn |

**Cross-Species Reference Similarity Mapping**

| **cluster** | **correlation_with_CONTROL** | **correlation_with_VTN** | **correlation_with_COMBINED** | **correlation_diff** |
| --- | --- | --- | --- | --- |
| cg-like SC | -0.298952932 | 0.298952931820282 | 0.0740329631639655 | 0.597905863640563 |
| GC2 | -0.276789309 | 0.276789309442967 | -0.113298789 | 0.553578618885936 |
| GC1 | -0.227224079 | 0.227224078902639 | 0.12391401183392 | 0.454448157805277 |
| GC3 | -0.170276628 | 0.170276628035215 | -0.343275395 | 0.340553256070433 |
| SC2 | -0.012638512 | 0.0126385115503434 | 0.0910260958549665 | 0.0252770231006862 |
| SC1 | 0.0628074288777899 | -0.062807429 | 0.0498256607385774 | -0.125614858 |
| BC3 | 0.113615616628817 | -0.113615617 | -0.08230984 | -0.227231233 |
| BC1 | 0.198869009954624 | -0.19886901 | 3.91811097881659e-05 | -0.39773802 |
| BC2 | 0.592529929350446 | -0.592529929 | 0.18600145721277 | -1.185059859 |
